# Synthesis of 1,5-Substituted Pyrrolidin-2-ones from Donor–Acceptor Cyclopropanes and Anilines/Benzylamines

**DOI:** 10.3390/molecules27238468

**Published:** 2022-12-02

**Authors:** Maksim A. Boichenko, Andrey Yu. Plodukhin, Vitaly V. Shorokhov, Danyla S. Lebedev, Anastasya V. Filippova, Sergey S. Zhokhov, Elena A. Tarasenko, Victor B. Rybakov, Igor V. Trushkov, Olga A. Ivanova

**Affiliations:** 1 Department of Chemistry, M. V. Lomonosov Moscow State University, Leninskie Gory 1-3, 119991 Moscow, Russia; 2 N. D. Zelinsky Institute of Organic Chemistry, Leninsky Pr. 47, 119334 Moscow, Russia

**Keywords:** donor–acceptor cyclopropanes, primary amines, pyrrolidin-2-ones, benz[*g*]indolizidines

## Abstract

We developed a straightforward synthetic route to pharmacologically important 1,5-substituted pyrrolidin-2-ones from donor–acceptor cyclopropanes bearing an ester group as one of the acceptor substituents. This method includes a Lewis acid-catalyzed opening of the donor–acceptor cyclopropane with primary amines (anilines, benzylamines, etc.) to γ-amino esters, followed by in situ lactamization and dealkoxycarbonylation. The reaction has a broad scope of applicability; a variety of substituted anilines, benzylamines, and other primary amines as well as a wide range of donor–acceptor cyclopropanes bearing (hetero)aromatic or alkenyl donor groups and various acceptor substituents can be involved in this transformation. In this process, donor–acceptor cyclopropanes react as 1,4-*C*,*C*-dielectrophiles, and amines react as 1,1-dinucleophiles. The resulting di- and trisubstituted pyrrolidin-2-ones can be also used in subsequent chemistry to obtain various nitrogen-containing polycyclic compounds of interest to medicinal chemistry and pharmacology, such as benz[*g*]indolizidine derivatives.

## 1. Introduction

**Figure 1:**
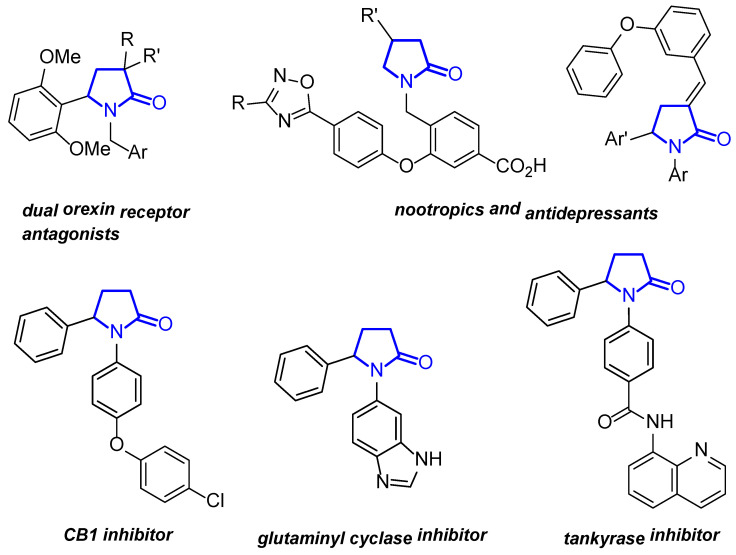
Bioactive molecules containing 1,5-diarylpyrrolidin-2-one or 5-aryl-1-benzylpyrrolidin-2-one frameworks.

The γ-lactam skeleton is a component of many biologically active molecules, both natural and synthetic, including approved drugs [1,2]. In particular, 1,5-diarylpyrrolidin-2-ones or 5-aryl-1-benzylpyrrolidones have great potential in pharmacology and medicinal chemistry (Figure 1). Among 1,5-diarylpyrrolidin-2-ones, there are selective and effective inhibitors of histone deacetylases 5 and 6 [3,4,5], cannabinoid receptor 1 (CB1) [6,7], cyclin-dependent kinase CDK_2_ [8], tankyrase [9], etc. They are also capable of inhibiting glutaminyl cyclase [10] and the glucagon receptor [11]. In addition, 5-aryl-1-benzylpyrrolidones have been shown to antagonize the dual orexin receptor at the submicromolar level [12,13] and calcitonin gene-related peptide type I receptors at the subnanomolar level [14]. Therefore, the synthesis of these promising azaheterocycles is an urgent problem in synthetic organic and pharmaceutical chemistry.

Although many methods for the γ-lactam synthesis are known [15,16,17,18], the development of new and simple strategies that also make it possible to introduce desired substituents into the resulting products remains an urgent task. Our interest in this problem is related to the possibility of solving it using the donor–acceptor (DA) cyclopropane [19,20,21,22,23,24,25,26,27,28,29,30,31,32,33] reactivity, which has been the subject of our studies in recent years [27,28,34,35,36,37,38,39].

**Scheme 1:**
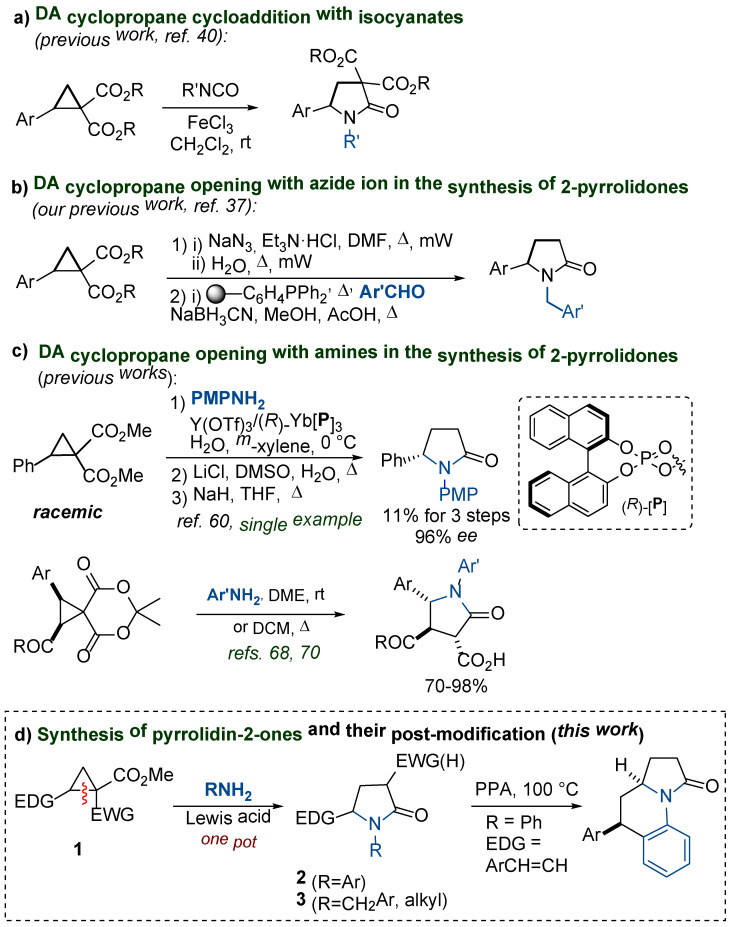
Synthesis of pyrrolidin-2-ones from DA cyclopropanes. (**a**–**c**) The reported methods for the synthesis of 1,5-substitutted pyrrolidine-2-ones from DA cyclopropanes. (**d**) Transformations reported in this work.

In general, two types of transformations of DA cyclopropanes can be used for the synthesis of 1,5-substituted pyrrolidin-2-ones. The first one is the (3 + 2)-cycloaddition of 2-aryl- or 2-alkenylcyclopropane-1,1-diesters with the appropriate isocyanates [40,41]. This process directly afforded the corresponding pyrrolidones (Scheme 1a); however, the resulting products contain two acceptor substituents at the C(3) atom; these groups must be removed to obtain the aforementioned bioactive compounds.

Alternatively, these DA cyclopropanes can undergo a small ring opening with *N*-nucleophiles followed by cyclization producing the target γ-lactams. For example, we have recently developed a method for the synthesis of 1,5-substituted pyrrolidin-2-ones, the key step of which is the opening of the DA cyclopropane ring with an azide ion (Scheme 1b) [37,38,39]. This method includes isolation and purification of the intermediate azides; a simpler and general approach to the synthesis of 1,5-disubstituted pyrrolidin-2-ones **2**, **3** can be developed based on the reaction of DA cyclopropanes **1** with the corresponding primary amines, such as anilines, benzylamines, etc.

The reactions of DA cyclopropanes with primary amines affording both acyclic and various cyclic products, depending on the structure of the reagents and reaction conditions, have been well studied [29,42,43,44,45,46,47,48,49,50,51,52,53,54,55,56,57,58,59,60,61,62,63,64,65,66,67,68,69,70]. However, only a few examples of the use of this reactivity for the synthesis of 1,5-functionalized pyrrolidin-2-ones have been described [59,60,61,62,63,64,65,66,67,68,69,70]. Usually, these examples were reported as postmodifications of the primary acyclic products [59,60,61,62,63,64] that provides not the principal advantage over other stepwise transformations. The one-step formation of the requisite pyrrolidones was achieved either on specific substrates [65,66,67,68,69,70] (Scheme 1c), i.e., has limited application, or proceeded under harsh conditions, giving pyrrolidones in moderate yields [62,64].

In this paper, we demonstrate that the transformation of DA cyclopropanes to 1,5-substituted pyrrolidones can be implemented as a *one-pot* process via a Lewis acid-initiated, three-membered ring opening with anilines, benzylamines, and other primary amines, followed by lactamization, as well as further modifications of the obtained pyrrolidones to polycyclic molecules such as benz[*g*]indolizidine derivatives (Scheme 1d).

## 2. Results and Discussion

We started our investigation with the study of the reaction of model cyclopropane **1a** with aniline, leading to the acyclic product **4a** (Table 1). To catalyze the reaction, we tested several available Lewis acids, which are commonly used for initiating reactions of DA cyclopropanes with *N*-nucleophiles. The reaction was carried out in dichloroethane (DCE) at room temperature for 1 h for all tested initiators. We found that Al(OTf)_3_ did not induce the target transformation (Table 1, entry 1). Conversely, in the presence of Fe(OTf)_3_, Sc(OTf)_3_, or Zn(OTf)_2_, cyclopropane **1a** reacted with aniline affording acyclic product **4a** in reasonable to good yields (Table 1, entries 2–5). The best results were achieved using 20 mol% Ni(ClO_4_)_2_∙6H_2_O or Y(OTf)_3_; with these catalysts, compound **4a** was obtained in more than a 90% yield (Table 1, entries 6, 9). The decrease in nickel perchlorate loading led to a decrease in the product yield (Table 1, entries 7–9). The yield also decreased with increasing reaction time or when the reaction was carried out with heating; in both cases, the formation of byproducts was detected. When Brønsted acid, TfOH, was used, no reaction occurred at all presumably due to its neutralization with an excess of amine (Table 1, entry 10). With all the studied Lewis acids, only the acyclic product **4a** was formed; its cyclization to pyrrolidin-2-one did not occur at room temperature.

**Scheme 2:**
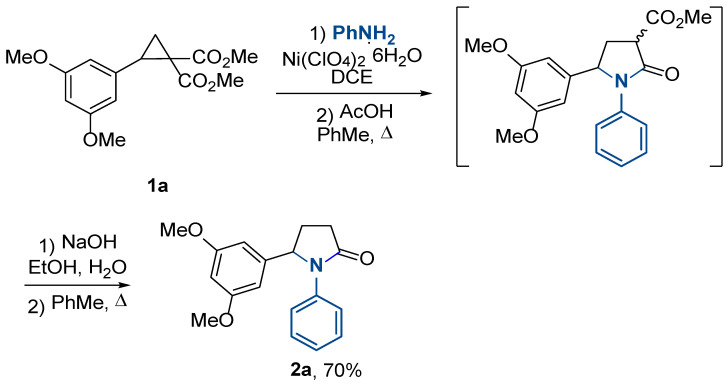
One-pot synthesis of pyrrolidone **2a**.

Then, the lactamization of γ-aminoester **4a** was investigated. We found that the cyclization of compound **4a** proceeded under the reflux of its toluene solution with acetic acid. Moreover, we showed that the crude reaction mixture obtained by a nickel perchlorate-induced reaction of cyclopropane **1a** with aniline, when refluxing with 2 equiv. acetic acid in toluene efficiently produced the corresponding pyrrolidin-2-one in a one-vessel operation. This compound was obtained as a mixture of two diastereomers due to the presence of an ester group at the C(3) atom of the pyrrolidone ring. To obtain the target bioactive 1,5-diarylpyrrolidin-2-ones, this group must be removed by one of the known dealkoxycarbonylation methods. To further simplify the synthetic sequence and increase the practicality of this strategy, we realized this transformation in *one pot* using alkaline saponification of the ester group followed by thermolysis (Scheme 2). As a result, pyrrolidone **2a** was synthesized by a four-step procedure, requiring chromatographic purification only at the last stage, with an overall yield of 70%.

**Scheme 3:**
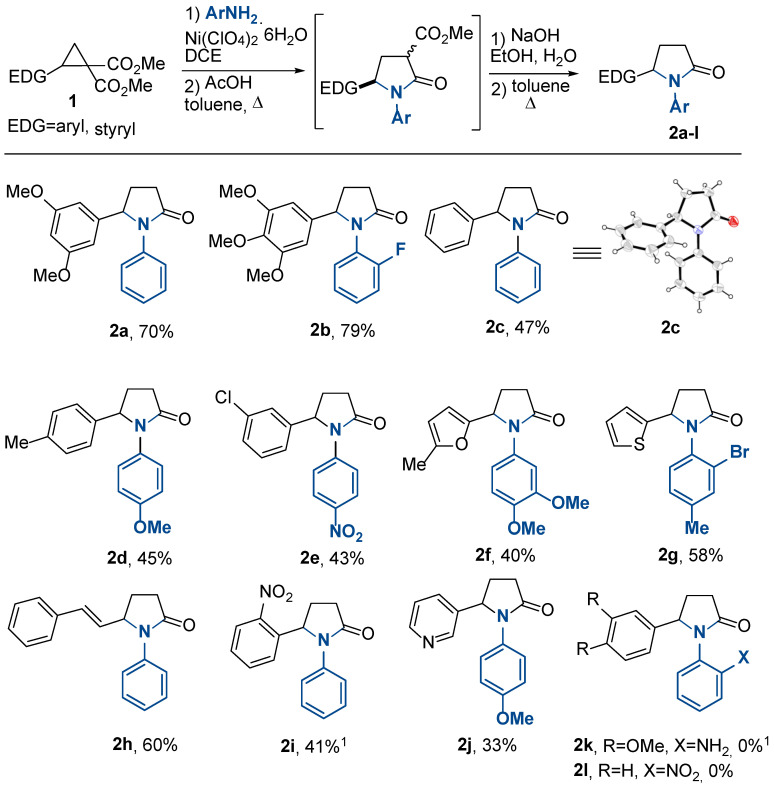
Synthesis of *N*-aryl and *N*-benzyl-substituted γ-lactams **2**, **3**. ^1^ Y(OTf)_3_ was used.

With the optimized conditions in hand, we investigated the reaction scope using diversly substituted DA cyclopropanes and a range of anilines (Scheme 3). We found that this *one pot* transformation was efficient for a series of DA cyclopropanes where the electron-rich het(aryl) group or styryl group was the donor substituent. A broad variety of substituents on the aromatic moiety of both DA cyclopropanes and anilines, such as halogen, alkyl, and alkoxy, were well-tolerated in these transformations. The yields of the obtained pyrrolidones **2** varied considerably from moderate to good; however, it should be taken into account that these yields were given for four-step procedures. This is also reflected in the complex dependence of the obtained yields on the structure of the starting compounds. For example, DA cyclopropanes bearing electron-abundant aromatic substituents are typically more reactive than DA cyclopropanes with less electron-rich donors; i.e., the conversion time is shorter. However, their side reactions also proceed faster, and that can provide lower yields of the target products. For multistage processes, the overall effect of the substituent on the reaction yields is even more complex and cannot be followed by any simple model.

For example, the moderate yield of pyrrolidone **2f** obtained from highly reactive furyl-substituted DA cyclopropane presumably resulted from the well-known tendency of the furan ring to undergo various acid-induced transformations [71,72]. In contrast, compound **2b** was formed in a 79% yield. Other reactive DA cyclopropanes, thienyl- and styryl-derived, produced the corresponding pyrrolidones **2g**,**h** in about a 60% yield. Less reactive 2-phenyl- and 2-(*p*-tolyl)cyclopropane-1,1-diesters produced the corresponding pyrrolidones **2c**,**d** in 47 and 45% yields. The structure of the compound **2c** was unambiguously proven by single-crystal X-ray data [73]. Cyclopropane-1,1-diesters containing the 2-nitrophenyl or 3-pyridyl groups at the C(2) atom of the small ring afforded the expected pyrrolidones **2i**,**j** in low yields. However, the yield of **2i** was improved by replacing the nickel perchlorate with 20 mol% Y(OTf)_3_. Anilines containing both electron-withdrawing and electron-donating substituents, including fluorine or bromine in the *ortho* position, reacted well. The exceptions were 4-nitroaniline, for which the first step occurred only when the reaction mixture was refluxed, and 2-nitroaniline and 1,2-phenylenediamine, which did not afford the desired products **2k**,**l** at all. With these anilines, the process was stopped after the formation of the open-chain products **4b**,**c** (see below); cyclization products were not detected in the reaction mixtures even in trace amounts.

To demonstrate the efficiency of this *one pot* process, we scaled up the synthesis of compound **2b** using 1.00 g (3.08 mmol) of 3,4,5-trimethoxyphenyl-substituted cyclopropane **1b** and 472 mg (3.08 mmol) of 2-fluoroaniline. With this loading, the yield of compound **2b** was 841 mg (79%).

It is worth noting that, despite the potential ability of anilines to serve as ambident nucleophiles, in the studied reactions, they attacked the three-membered ring exclusively with the nitrogen atom, providing no isomeric products via the Friedel–Crafts alkylation of the electron-rich aromatic ring.

**Scheme 4:**
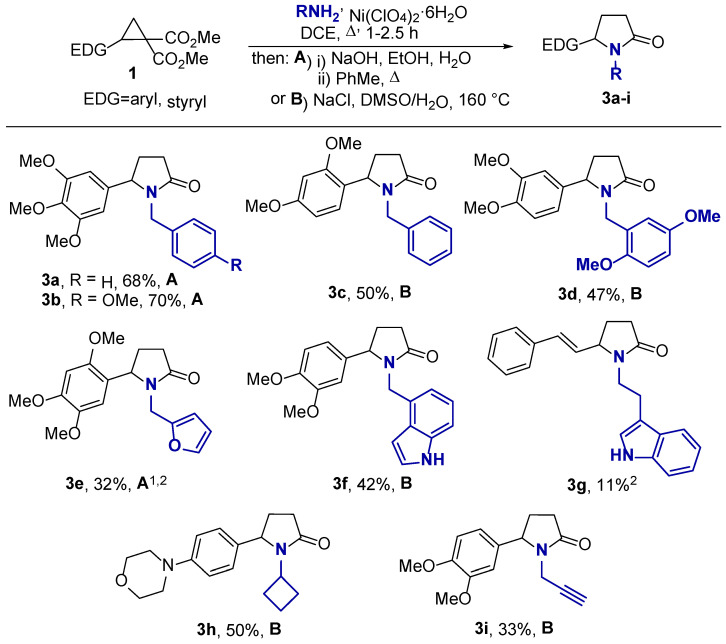
Synthesis of γ-lactams **3**. ^1^ The reaction was performed at room temperature. ^2^ The reaction included a lactamization step under reflux in presence of AcOH in toluene.

Benzylamines are known to be more nucleophilic than the corresponding anilines. The increased reactivity of benzylamines allowed us to synthesize pyrrolidones **3** in good yields by their direct reaction with DA cyclopropanes **1** by refluxing a dichloroethane solution in the presence of Ni(ClO_4_)_2_·6H_2_O without an additional lactamization step. Next, we used two methods of dealkoxycarbonylation of 3-substituted pyrrolidones. The first one included alkaline hydrolysis followed by decarboxylation according to the method developed for pyrrolidones **2** (method **A**, Scheme 4). Alternatively, dealkoxycarbonylation using a NaCl-promoted Krapcho reaction in wet DMSO at 160 °C under microwave (MW) irradiation provided pyrrolidones **3** in reasonable yields (method **B**, Scheme 4). For example, benzylamine and alkoxy-substituted benzylamines produced compounds **3a**–**d** in up to 70% yields. In the reactions of DA cyclopropane with furfurylamine and (1*H*-indol-3-yl)methylamine, the corresponding pyrrolidones **3e** and **3f** were obtained in 32% and 42% yields. Given that these yields corresponded to a four-step sequence realized as a *one pot* procedure, these yields can be considered reasonable.

Moreover, we applied the developed approach to the synthesis of 1-alkyl-5-arylpyrrolidones from DA cyclopropanes and some aliphatic amines (Scheme 4). Cyclobutylamine and propargylamine were found to participate quite efficiently in this transformation, affording the corresponding pyrrolidones **3h**,**i** in yields close to those of **3c**–**f**, although these substrates required a long reaction time (see Section 3). In contrast, tryptamine gave rise to the corresponding **3g** product in only an 11% yield. A significant tarring of the reaction mixture was detected in this reaction. When simple primary aliphatic amines, such as methylamine or ethylamine, were reacted with cyclopropane **1a** under the same reaction conditions, only unidentified byproducts were formed.

**Scheme 5:**
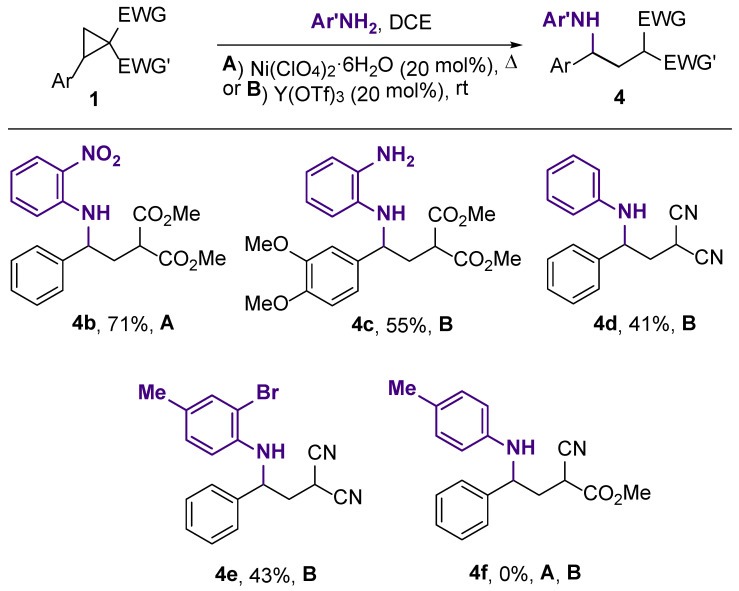
Synthesis of acyclic products **4b**–**f**.

It was pointed out that above that, *o*-nitroaniline and 1,2-phenylenediamine did not produce the target products **2** under standard conditions. We tested their reactivity toward DA cyclopropanes **1** in the presence of the same catalysts in more detail (Scheme 5). We found that the full consumption of 2-phenylcyclopropane-1,1-dicarboxylate **1k** in its reaction with *o*-nitroaniline catalyzed by Ni(ClO_4_)_2_·6H_2_O required 2 h of refluxing the solution in dichloroethane. Under these conditions, the expected acyclic product **4b** was obtained in a 71% yield. The reaction of 3,4-dimethoxyphenyl-substituted cyclopropane **1j** with 1,2-phenylenediamine under the same conditions produced the acyclic product **4c** only in a low yield. Heating this reaction mixture at 100 °C in chlorobenzene resulted in a complex mixture of unidentified products. However, the acyclic compound **4c** was obtained in a reasonable yield at room temperature using Y(OTf)_3_ as an initiator (Scheme 5). This product was unstable, and all attempts to cyclize it were unsuccessful.

To test the generality of the DA cyclopropane ring opening with anilines, we tried to involve substrates with other acceptor groups in this reaction (Scheme 5). 2-Phenylcyclopropane-1,1-dicarbonitrile was found to undergo ring opening with aniline or 2-bromo-4-methylaniline under catalysis with 25 mol% Y(OTf)_3_ at room temperature for 4 days. The full conversion of these substrates required a significantly longer reaction time compared to the corresponding 1,1-diesters. Despite the mild reaction conditions, **4d**,**e** were isolated only in 41% and 43% yields, presumably due to the competitive realization of the side processes resulting from the coexistence of the amino and cyano groups. 2-Phenyl-1-cyanocyclopropanecarboxylate turned out to be a less reactive substrate, which did not undergo conversion to amine **4f** even at a high temperature. Other Lewis acids, such as Fe(OTf)_3_, Sc(OTf)_3_, and Ni(ClO_4_)_2_·6H_2_O, failed also to induce the reaction of this substrate with anilines.

**Scheme 6:**
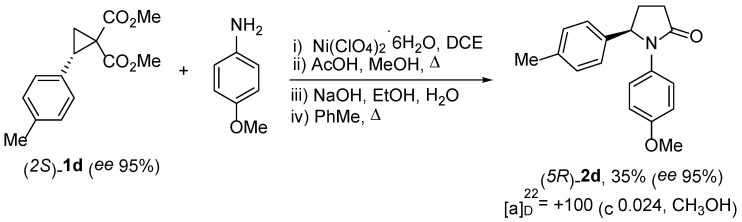
Synthesis of (*R*)-γ-lactam (**2d**).

Most biologically active compounds bearing chiral centers have different activities for different stereoisomers. This means that methods of their preparation in an optically pure form are highly desirable. Obviously, this also applies to bioactive-substituted pyrrolidones of types **2** and **3**, which can be considered as cyclic analogs of GABA. We tested the possibility of using the developed procedure for the synthesis of chiral pyrrolidones **2** starting from optically active DA cyclopropane **1** as a substrate. We found that dimethyl (*S*)-2-(*p*-tolyl)cyclopropane-1,1-dicarboxylate (*S*)-**1c** was converted to the corresponding γ-lactam with a full inversion of the absolute configuration of the chiral center (Scheme 6). This result is consistent with previous investigations demonstrating that the nucleophilic ring opening of DA cyclopropanes under catalysis with moderately activating Lewis acids proceeds by an S*_N_*2-like mechanism, and subsequent stages (cyclization, saponification, and decarboxylation) do not affect the chiral center.

**Scheme 7:**
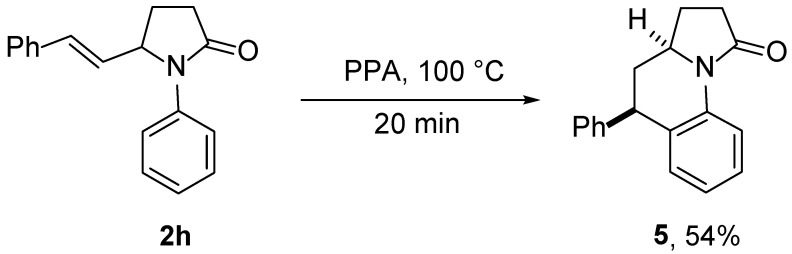
Synthesis of benz[*g*]indolizidine **5**.

The synthetic utility of the developed transformations can be significantly extended by diverse postmodifications of their multiple functionalities of the synthesized pyrrolidones **2**,**3**, that allows for preparing complex azaheterocycles. In particular, the treatment of pyrrolidone **2h** with PPA at 100 °C produced benz[*g*]indolizidine **5** in an acceptable yield (Scheme 7). Based on NOESY spectroscopy [73] and a comparison of its spectra with spectral data for the related compounds described earlier [74], the hydrogen atoms at the stereogenic centers in compound **5** have a *cis* arrangement.

## 3. Experimental Section

### 3.1. General Information

The structures of synthesized compounds were elucidated with the aid of 1D NMR (^1^H, ^13^C) and 2D NMR (NOESY, HSQC and HMBC ^1^H-^13^C) spectroscopy. NMR spectra were acquired on Avance 600 and Avance 500 (Bruker, Billerica, MA, USA) and 400-MR (Agilent, Santa Clara, CA, USA) spectrometers at room temperature; the chemical shifts δ were measured in ppm with respect to solvent (1H: CDCl_3_, δ = 7.27 ppm; CD_3_OD, δ = 3.35 ppm; ^13^C: CDCl_3_, δ = 77.0 ppm; CD_3_OD: ^13^C: δ = 49.9 ppm). Splitting patterns were designated as s, singlet; d, doublet; m, multiplet; dd, double doublet; and br, broad. Coupling constants (J) were in Hertz. Infrared spectra were recorded on an FTIR spectrometer ALPHA II (Bruker, Billerica, MA, USA) in KBr for solid substances and as thin film for oils. High resolution and accurate mass measurements were carried out using a micrOTOF-Q^TM^ ESI-TOF (Electrospray Ionization/Time of Flight, Bruker, Billerica, MA, USA). Elemental analyses were performed with an EA-1108 CHNS elemental analyzer instrument (Fisons, Ipswich, UK). Melting points (mp) were measured using a Stuart^®^ SMP3 melting point apparatus (Cole-Parmer, Stone, Staffordshire, UK). Microwave reactions were performed in a Monowave 200—Anton Paar microwave reactor in sealed reaction vessels. The temperature was monitored with installed IR detector. X-Ray analysis was performed on STOE STADI VARI PILATUS-100K diffractometer (Stoe & Sie, Darmstadt, Germany). Analytical thin-layer chromatography (TLC) was carried out with silica gel plates (silica gel 60, F_254_, supported on aluminum); visualization was performed using a UV lamp (365 nm). Column chromatography was performed on silica gel 60 (230–400 mesh, Merck, Darmstadt, Germany). Enantiomeric purity of the optically active compounds was determined by chiral HPLC with a Hitachi LaChrome Elite-2000 chromatograph (Hitachi Hugh-Tech Corp, Toranomonon Minato-Ku, Japan) using a Daicel (Daicel Corp, Osaka, Japan) Chiralcel OD-H column (0.46 × 25 cm) at room temperature. The column was eluted with heptane/*i*-PrOH = 70:30 at a flow rate of 1 mL/min, and peak detection was accomplished using a UV detector at 219 nm. Optical rotation was measured on a Krüss P8000 polarimeter (A. Krüss Optronic GmbH, Hamburg, Germany). All reactions were carried out using freshly distilled and dry solvents. Cyclopropanes **1** were prepared by Knoevenagel/Corey–Chaykovsky reaction sequences from the corresponding aldehydes [75,76]. Compounds **2c**,**j**, **3b**,**c**, and (2*S*)-**1d** were described previously [15,16,17,39,77,78]. Commercial reagents employed in the synthesis were analytical-grade, obtained from Sigma-Aldrich (St. Louis, MO, USA) or Alfa Aesar (Ward Hill, MA, USA). The ^1^H NMR and ^13^C NMR for synthesized compounds as well as 2D (HSQC and HMBC) NMR spectra for selected compounds are available in the Supplementary Materials.

### 3.2. Synthesis of Pyrrolidin-2-ones 2,3 from Anilines and Benzylamines

#### 3.2.1. General Procedure 1

To a 0.2 M solution of aniline or benzylamine (1.0–1.2 equiv.) in DCE in the presence of molecular sieves, 4 Å Ni(ClO_4_)_2_∙6H_2_O or Y(OTf)_3_ (0.2 equiv.) was added under Ar atmosphere; then, cyclopropane **1** (1–4 mmol, 1.0 equiv.) was added. The resulting mixture was stirred at room temperature for 1–3 h, diluted with dichloromethane (DCM), and filtered through a short pad of silica gel using EtOAc as the eluent. The filtrate was concentrated under vacuum; the residue was dissolved in toluene (0.13 M). Next, acetic acid (2.0 equiv.) was added, and the reaction mixture was stirred under reflux for 7 h. Then, solvent was removed under vacuum, and residue was dissolved in ethanol (0.17 M); 1M aq. solution of sodium hydroxide (2.0 equiv.) was added in one portion. The reaction mixture was stirred at room temperature for 2 h, and after that, ethanol was removed under vacuum. The residue was diluted with water, and 1M HCl was added until pH 1. The resulting mixture was extracted with ethyl acetate (3 × 10 mL). Combined organic layers were dried with Na_2_SO_4_ and concentrated in vacuo. The residue was dissolved in toluene (0.07 M) and was refluxed for 7 h. The solvent was removed under vacuum; the pure product was isolated by silica gel column chromatography.

#### 3.2.2. General Procedure 2

To a 2 M solution of cyclopropane **1** (1 equiv.) and benzylamine (1.2 equiv.) in DCM or DCE in the presence of molecular sieves, 4 Å Ni(ClO_4_)_2_∙6H_2_O (0.1 equiv.) was added under Ar atmosphere The reaction mixture was placed into oil bath, which was preheated to 45 °C, and stirred at the same temperature for 1–2.5 h, and after, it was cooled to room temperature and diluted with DCM. The resulting solution was passed through a plug of silica using 1:1 petroleum ether:EtOAc system as an eluent. Concentration under reduced pressure gave a residue, which was dissolved in DMSO/H_2_O mixture (3:1, 0.16 M). To this solution, NaCl (1.5 equiv.) was added; the resulting mixture was heated at 160 °C under MW irradiation for 4–6 h. Then, the reaction mixture was diluted with H_2_O and extracted with EtOAc three times. Combined organic layers were washed with brine, dried with Na_2_SO_4_, and concentrated in vacuo. Pure product was isolated by silica gel column chromatography.

*5-(3,5-Dimethoxyphenyl)-1-phenylpyrrolidin-2-one* (**2a**) was obtained from dimethyl 2-(3,5-dimethoxyphenyl)cyclopropane-1,1-dicarboxylate (300 mg, 1.02 mmol), aniline (0.1 mL, 1.10 mmol), Ni(ClO_4_)_2_∙6H_2_O (75 mg, 0.2 mmol), DCE (5.1 mL), AcOH (120 μL), toluene (8.0 mL), NaOH (84 mg, 2.1 mmol), ethanol (5.9 mL), and water (2.0 mL) according to the general procedure **1**. Yield: 210 mg (69%); white solid, *R_f_* = 0.34 (ethyl acetate:petroleum ether; 1:1). ^1^H NMR (CDCl_3_, 600 MHz): δ 7.44 (d, ^3^*J* = 7.9 Hz, 2H, Ph), 7.27–7.25 (m, 2H, Ph), 7.08–7.06 (m, 1H, Ph), 6.37 (d, ^4^*J* = 2.0 Hz, 2H, Ar), 6.33–6.32 (m, 1H, Ar), 5.17 (dd, ^3^*J* = 7.2 Hz, ^3^*J* = 4.2 Hz, 1H, CH), 3.73 (s, 6H, 2 × CH_3_O), 2.80–2.74 (m, 1H, CH_2_), 2.64–2.56 (m, 2H, CH_2_), 2.03–1.97 (m, 1H, CH_2_). ^13^C{^1^H} NMR (CDCl_3_, 150 MHz): δ 174.8 (CO), 161.3 (2 × C), 144.0 (C), 138.3 (C), 128.7 (2 × CH), 124.9 (CH), 122.0 (2 × CH), 103.9 (2 × CH), 99.2 (CH), 63.9 (CH), 55.3 (2 × CH_3_O), 31.3 (CH_2_), 29.3 (CH_2_). IR (KBr, cm^−1^) 2996, 2980, 2941, 2904, 2874, 2840, 1693, 1654, 1613, 1594, 1538, 1499, 1490, 1482, 1461, 1443, 1429, 1370, 1352, 1318, 1284, 1250, 1227, 1201, 1162, 1145, 1117, 1066, 1030, 1011, 978. HRMS ESI-TOF: *m*/*z* = 298.1435 [M + H]^+^ (298.1438 calcd. for C_18_H_20_NO_3_^+^).

*5-(3,4,5-Trimethoxyphenyl)-1-(2-fluorophenyl)pyrrolidin-2-one* (**2b**) was obtained from dimethyl 2-(3,4,5-dimethoxyphenyl)cyclopropane-1,1-dicarboxylate (1.00 g, 3.08 mmol), 2-fluoroaniline (472 mg, 3.08 mmol), Ni(ClO_4_)_2_∙6H_2_O (226 mg, 0.62 mmol), DCE (15.4 mL), AcOH (355 μL), toluene (26.7 mL), NaOH (247 mg, 6.18 mmol), ethanol (17.8 mL), and water (3.1 mL) according to the general procedure **1**. Yield: 841 mg (79%); yellowish viscous oil, *R_f_* = 0.42 (ethyl acetate:petroleum ether; 1:2). ^1^H NMR (CDCl_3_, 400 MHz): δ 7.19–7.10 (m, 2H, Ar), 7.04–7.00 (m, 2H, Ar), 6.43 (s, 2H, Ar), 5.14–5.11 (m, 1H, CH), 3.75 (s, 6H, 2 × CH_3_O), 3.74 (s, 3H, CH_3_O), 2.78–2.58 (m, 3H), 2.09–2.00 (m, 1H). ^13^C{^1^H} NMR (CDCl_3_, 100 MHz): δ 174.7 (CO), 157.2 (d, ^1^*J*_CF_ = 250 Hz), 153.2 (2 × C), 137.1 (C), 136.3 (C), 128.4 (d, ^3^*J*_CF_ = 7 Hz, CH), 127.9 (CH), 125.0 (d, ^2^*J*_CF_ = 12 Hz, C), 124.1 (CH), 116.4 (d, ^2^*J*_CF_ = 20 Hz, CH), 103.0 (2 × CH), 64.7 (CH), 60.5 (CH_3_O), 55.8 (2 × CH_3_O), 30.5 (CH_2_), 29.6 (CH_2_). IR (film, cm^−1^): 2840, 2827, 1703, 1593, 1504, 1237, 1131. HRMS (ESI/TOF): *m*/*z* = 346.1449 [M + H]^+^ (346.1449 calcd. for C_19_H_21_FNO_4_^+^).

*1,5-Diphenylpyrrolidin-2-one* (**2c**) [17] was obtained from dimethyl 2-phenylcyclopropane-1,1-dicarboxylate (100 mg, 0.4 mmol), aniline (38.6 μL, 0.4 mmol), Ni(ClO_4_)_2_∙6H_2_O (30.8 mg, 0.08 mmol), DCE (2 mL), AcOH (46 μL), toluene (2.7 mL), NaOH (35 mg, 0.8 mmol), ethanol (2.5 mL), and water (0.4 mL) according to the general procedure. Yield: 47 mg (47%); yellow crystals; mp = 109–112 °C (dec.); lit. 100 °C [17]; 106– 108 [16]; R_f_ = 0.25 (ethyl acetate:petroleum ether; 1:3). ^1^H NMR (CDCl_3_, 600 MHz): δ 7.43 (d, ^3^*J* = 8.0 Hz, 2H, Ph), 7.32–7.30 (m, 2H, Ph), 7.26–7.22 (m, 5H, Ph), 7.07 (m, 1H, Ph), 5.27–5.25 (m, 1H), 2.80–2.74 (m, 1H), 2.67–2.59 (m, 2H), 2.04–1.98 (m, 1H). ^13^C{^1^H} NMR (CDCl_3_, 150 MHz MHz): δ 175.0, 141.4, 138.3, 129.1 (2 × C), 128.8 (2 × C), 127.8, 126.0 (2 × C), 125.0, 122.2 (2 × C), 64.0, 31.3, 29.3. The spectral data were in accordance with the literature [17]. IR (KBr, cm^−1^): 3379, 3107, 3095, 3083, 3042, 3028, 2992, 2969, 2952, 2895, 1709, 1596, 1497, 1480, 1456, 1415, 1367, 1356, 1324, 1308, 1281, 1238, 1224, 1194, 1179, 1194, 1114, 1077, 1052, 1031, and 1002. HRMS (ESI/TOF): *m*/*z* = 238.1226 [M + H]^+^ (238.1228 calcd. for C_16_H_16_NO^+^).

*1-(4-Methoxyphenyl)-5-(p-tolyl)pyrrolidin-2-one* (**2d**) was obtained from dimethyl 2-(4-methylphenyl)cyclopropane-1,1-dicarboxylate (182 mg, 0.73 mmol), *p*-anisidine (91 mg, 0.73 mmol), Ni(ClO_4_)_2_∙6H_2_O (55 mg, 0.15 mmol), DCE (5.0 mL), AcOH (90 μL), toluene (4.9 mL), NaOH (55.6 mg, 1.39 mmol), ethanol (3.9 mL), and water (1.4 mL) according to the general procedure **1**. Yield: 92 mg (45%); ivory solid; mp 112–114 °C; *R_f_* = 0.47 (ethyl acetate:petroleum ether; 2:1). ^1^H NMR (CDCl_3_, 600 MHz): δ 7.28 (d, ^3^*J* = 8.9 Hz, 2H, Ar), 7.10 (s, 4H, Ar), 6.76 (d, ^3^*J* = 8.9 Hz, 2H, Ar), 5.14 (dd, ^3^*J* = 7.4 Hz, ^3^*J* = 4.6 Hz, 1H, CH), 3.63 (s, 3H, CH_3_O), 2.77–2.70 (m, 1H, CH_2_), 2.62–2.55 (m, 2H, CH_2_), 2.29 (s, 3H, CH_3_), 2.00–1.95 (m, 1H, CH_2_). ^13^C NMR (CDCl_3_, 150 MHz): δ 174.6 (CO), 156.8 (C), 138.4 (C), 137.4 (C), 131.2 (C), 129.5 (2 × CH), 126.0 (2 × CH), 124.2 (2 × CH), 113.9 (2 × CH), 64.1 (CH), 55.2 (CH_3_O), 31.1 (CH_2_), 29.1 (CH_2_), 21.0 (CH_3_). IR (KBr, cm^−1^): 2835, 1694, 1512, 1248, 1035. HRMS (ESI/TOF): *m*/*z* = 282.1492 [M + H]^+^ (282.1489 calcd. for C_18_H_20_NO_2_^+^). Anal. calcd. for C_18_H_19_NO_2_: C, 76.84; H, 6.81, N, 4.95. Found: C, 76.63; H, 6.85; N, 4.98.

*(R)-1-(4-Methoxyphenyl)-5-(p-tolyl)pyrrolidin-2-one* (5*R*)-**2d**) was obtained from dimethyl (*S*)-2-(*p*-tolyl)cyclopropane-1,1-dicarboxylate [79] (80 mg, 0.32 mmol, *ee* 95%, [α]_D_^20^ –130° (c 1.0, CHCl_3_)), 4-methoxyaniline (40 mg, 0.32 mmol), Ni(ClO_4_)_2_∙6H_2_O (24 mg, 0.066 mmol), DCE (1.6 mL), AcOH (37 μL), toluene (2.1 mL), NaOH (27 mg, 0.68 mmol), ethanol (1.9 mL), and water (0.65 mL) according to the general procedure. Yield 32 mg (35%); ivory solid; mp 112–114 °C; [α]_D_^22^ = +100 (c 0.024, CH_3_OH); *R*_f_ = 0.47 (ethyl acetate:petroleum ether; 2:1). Spectral data were identical to those of **2d**.

*5-(3-Chlorophenyl)-1-(4-nitrophenyl)pyrrolidin-2-one* (**2e**) was obtained from dimethyl 2-(3-chlorophenyl)cyclopropane-1,1-dicarboxylate (300 mg, 1.11 mmol), 4-nitroaniline (170 mg, 1.22 mmol), and Ni(ClO_4_)_2_∙6H_2_O (82 mg, 0.223 mmol) in DCE (5.6 mL) according to the modified general procedure **1** (2.5 h under reflux for the first step and 12 h for the second step). Yield: 151 mg (43%); yellowish solid, mp 64–66 °C, *R_f_* = 0.56 (ethyl acetate:petroleum ether; 1:1). ^1^H NMR (CDCl_3_, 600 MHz): δ 8.09 (d, ^3^*J* = 9.6 Hz, 2H, Ar), 7.66 (d, ^3^*J* = 9.6 Hz, 2H, Ar), 7.30–7.22 (m, 2H, Ar), 7.20 (s, 1H, Ar), 7.07 (dt, ^3^*J* = 7.2 Hz, ^4^*J* = 1.8 Hz, 1H, Ar), 5.30 (dd, ^3^*J* = 7.9 Hz, ^3^*J* = 4.2 Hz, 1H, C(5)H), 2.83–2.60 (m, 3H, C(3)H_2_, C(4)H_2_), 2.07–1.99 (m, 1H, C(4)H_2_). ^13^C NMR (CDCl_3_, 150 MHz): δ 175.2 (CO), 143.7 (C), 143.6 (C), 142.4 (C), 135.4 (C), 130.7 (CH), 128.5 (CH), 125.8 (CH), 124.5 (2 × CH), 123.6 (CH), 120.7 (2 × CH), 62.8 (C(5)H), 31.0 (C(3)H_2_), 28.8 (C(4)H_2_). IR (KBr, cm^−1^): 1708, 1592, 1510, 1496, 1335, 1323, 1296, 1286, 1219, 1194, 112, 848. HRMS ESI-TOF: *m*/*z* = 317.0687 [M + H]^+^ (317.0687 calcd. for C_16_H_14_N_2_O_3_^+^).

*1-(3,4-Dimethoxyphenyl)-5-(5-methylfuran-2-yl)pyrrolidin-2-on*e (**2f**) was obtained from dimethyl 2-(5-methylfuran-2-yl)cyclopropane-1,1-dicarboxylate (300 mg, 1.26 mmol), 3,4-dimethoxyaniline (193 mg, 1.26 mmol), Ni(ClO_4_)_2_∙6H_2_O (92.0 mg, 0.25 mmol), DCE (5.6 mL), AcOH (144 μL), toluene (8 mL), NaOH (104 mg, 2.6 mmol), ethanol (7.3 mL), and water (2.5 mL) according to the general procedure **1** (at 0 °C for the first step). Yield: 150 mg (40%); yellowish oil, *R_f_* = 0.39 (ethyl acetate:petroleum ether; 2:1). ^1^H NMR (CDCl_3_, 600 MHz): δ 6.87 (d, ^4^*J* = 2.0 Hz, 1H, Ar), 6.78 (d, ^3^*J* = 8.6 Hz, 1H, Ar), 6.73 (dd, ^3^*J* = 8.6 Hz, ^4^*J* = 2.0 Hz, 1H, Ar), 6.00 (d, ^3^*J* = 2.7 Hz, 1H, Fu), 5.83 (d, ^3^*J* = 2.7 Hz, 1H, Fu), 5.05–5.00 (m, 1H, CH), 3.83 (s, 3H, CH_3_O), 3.79 (s, 3H, CH_3_O), 2.87–2.81 (m, 1H, CH_2_), 2.62–2.56 (m, 1H, CH_2_), 2.53–2.46 (m, 1H, CH_2_), 2.33–2.28 (m, 1H, CH_2_), 2.25 (s, 3H, CH_3_). ^13^C NMR (CDCl_3_, 150 MHz): δ 174.4 (CO), 152.0 (C), 151.0 (C), 148.6 (C), 147.0 (C), 130.9 (C), 116.3 (CH), 110.8 (CH), 108.8 (CH), 108.4 (CH), 106.1 (CH), 58.7 (CH), 55.8 (CH_3_O), 55.6 (CH_3_O), 30.9 (CH_2_), 25.2 (CH_2_), 13.4 (CH_3_). IR (film, cm^−1^): 2837, 1674, 1565, 1511, 1239, 1022, 911, 538. HRMS ESI-TOF: *m*/*z* = 302.1389 [M + H]^+^ (302.1387 calcd. for C_17_H_20_NO_4_^+^).

*1-(2-Bromo-4-methylphenyl)-5-(thiophen-2-yl)pyrrolidin-2-one* (**2g**) was obtained from dimethyl 2-(thiophen-2-yl)cyclopropane-1,1-dicarboxylate (200 mg, 0.79 mmol), 2-bromo-4-methylaniline (147 mg, 0.78 mmol), Ni(ClO_4_)_2_∙6H_2_O (61.8 mg, 0.17 mmol), DCE (4.2 mL), AcOH (96 μL), toluene (5.3 mL), NaOH (63 mg, 1.57 mmol), ethanol (4.8 mL), and water (1.7 mL) according to the general procedure **1**. Yield: 162 mg (58%); colorless solid; mp 127–129 °C; *R_f_* = 0.54 (ethyl acetate). ^1^H NMR (CDCl_3_, 600 MHz): δ 7.26 (dd, ^3^*J* = 8.2 Hz, ^4^*J* = 2.0 Hz, 1H, Ar), 7.22 (d, ^3^*J* = 5.0 Hz, 1H, Ar), 7.15 (br. s, 1H, Ar), 7.05 (d, ^3^*J* = 8.2 Hz, 1H, Ar), 6.87–6.84 (m, 1H, Ar), 6.83–6.81 (m, 1H, Ar), 5.32–5.26 (m, 1H, CH), 2.87–2.81 (m, 1H, CH_2_), 2.77–2.71 (m, 1H, CH_2_), 2.70–2.64 (m, 1H, CH_2_), 2.41–2.34 (m, 1H, CH_2_), 2.11 (s, 3H, CH_3_). ^13^C NMR (CDCl_3_, 150 MHz): δ 173.7 (CO), 143.5 (C), 137.3 (C), 135.7 (C), 132.4 (CH), 130.9 (CH), 130.0 (C), 126.9 (CH), 126.8 (CH), 125.8 (CH), 119.1 (CH), 60.7 (CH), 30.9 (CH_2_), 29.6 (CH_2_), 18.0 (CH_3_). IR (KBr, cm^−1^): 3100, 2853, 1694, 1528, 716, 525. HRMS ESI-TOF: *m*/*z* = 336.0052 [M + H]^+^ (336.0052 calcd. for C_15_H_15_ Br^79^NOS^+^).

*(E)-1-Phenyl-5-styrylpyrrolidin-2-one* (**2h**) was obtained from dimethyl 2-(*E*)-styrylcyclopropane-1,1-dicarboxylate (300 mg, 1.15 mmol), aniline (107 mg, 1.15 mmol), Ni(ClO_4_)_2_∙6H_2_O (84 mg, 0.23 mmol), DCE (5.7 mL), AcOH (140 μL), toluene (8 mL), NaOH (99 mg, 2.48 mmol), ethanol (6.9 mL), and water (2.4 mL) according to the general procedure **1**. Yield: 182 mg (60%); yellow solid; mp 71–73 °C; *R_f_* = 0.38 (ethyl acetate:petroleum ether; 1:1). ^1^H NMR (CDCl_3_, 600 MHz): δ 7.50 (d, ^3^*J* = 8.6 Hz, 2H, Ar), 7.36–7.23 (m, 7H, Ar), 7.16–7.13 (m, 1H, Ar), 6.52 (d, ^3^*J* = 15.8 Hz, 1H, CH=), 6.14 (dd, ^3^*J* = 15.8 Hz, ^3^*J* = 7.4 Hz, 1H, CH=), 4.86–4.82 (m, 1H), 2.76–2.70 (m, 1H), 2.62–2.56 (m, 1H), 2.50–2.44 (m, 1H), 2.04–1.99 (m, 1H). ^13^C NMR (CDCl_3_, 150 MHz): δ 174.5, 138.2, 136.1, 132.2, 129.0, 128.9 (2 × C), 128.7 (2 × C), 128.1, 126.6 (2 × C), 125.4, 123.0 (2 × C), 62.4, 31.3, 26.7. IR (KBr, cm^−1^): 3059, 3026, 2978, 2943, 2873, 1699, 1695, 1598, 1529, 1497, 1456, 1449, 1382, 1294, 1219, 1210, 1154, 1115, 1072, 1042, 1029, 968. HRMS (ESI/TOF): *m*/*z* = 286.1202 [M+Na]^+^ (286.1202 calcd. for C_18_H_17_NNaO^+^).

*5-(2-Nitrophenyl)-1-phenylpyrrolidin-2-one* (**2i**) was obtained from dimethyl 2-(2-nitrophenyl)cyclopropane-1,1-dicarboxylate (300 mg, 1.07 mmol), aniline (120 mg, 1.29 mmol), Y(OTf)_3_ (118 mg, 0.22 mmol), DCE (5.4 mL), AcOH (123 μL), toluene (8 mL), NaOH (87 mg, 2.18 mmol), ethanol (6.2 mL), and water (2 mL) according to the general procedure **1**. Yield: 124 mg (41%); thick dark brown oil, *R_f_* = 0.27 (ethyl acetate:petroleum ether; 1:3). ^1^H NMR (CDCl_3_, 600 MHz): δ 8.09–8.07 (m, 1H, Ar), 7.56–7.54 (m, 1H, Ar), 7.44–7.42 (m, 3H, Ar), 7.40–7.38 (m, 1H, Ar), 7.28–7.26 (m, 2H, Ar), 7.10–7.07 (m, 1H, Ar), 5.99 (dd, ^3^*J* = 8.7 Hz, ^3^*J* = 3.4 Hz, 1H, CH), 2.92–2.85 (m, 1H, CH_2_), 2.75–2.64 (m, 2H, CH_2_), 2.07–2.02 (m, 1H, CH_2_). ^13^C NMR (CDCl_3_, 150 MHz): δ 175.1 (CO), 137.0 (C), 134.2 (CH), 129.1 (2 × CH), 128.8 (CH), 128.5 (C), 127.3 (CH), 126.8 (C), 125.8 (CH), 125.3 (CH), 121.5 (2 × CH), 59.7 (CH), 30.8 (CH_2_), 28.1 (CH_2_). IR (film, cm^−1^): 3308, 3199, 3136, 3103, 3066, 3043, 2953, 2924, 2853, 1699, 1598, 1579, 1526, 1498, 1457, 1444, 1382, 1348, 1295, 1251, 1225, 1162, 1118, 1073, 1040. HRMS ESI-TOF: *m*/*z* = 283.1077 [M + H]^+^ (283.1077 calcd. for C_16_H_15_N_2_O_3_^+^).

*1-(4-Methoxyphenyl)-5-(pyridin-3-yl)pyrrolidin-2-one* (**2j**) [15] was obtained from dimethyl 2-(pyridin-3-yl)cyclopropane-1,1-dicarboxylate (250 mg, 1.06 mmol), 4-methoxyaniline (131 mg, 1.06 mmol), Ni(ClO_4_)_2_∙6H_2_O (78 mg, 0.21 mmol), DCE (5.3 mL), AcOH (122 μL), toluene (6.7 mL), NaOH (77 mg, 1.9 mmol), ethanol (5.4 mL), and water (1.9 mL) according to the general procedure **1** but the product of hydrolysis was refluxed in water (14 mL, 0.065 M) for 14 h. Yield: 95 mg (33%); brown solid; mp 72–73 °C; lit. 73–75 °C [17]; *R_f_* = 0.14 (ethyl acetate). ^1^H NMR (CDCl_3_, 600 MHz): δ 8.51–8.50 (m, 1H, Ar), 8.48–8.47 (m, 1H, Ar), 7.52–7.50 (m, 1H, Ar), 7.23–7.20 (m, 3H, Ar), 6.77 (d, ^3^*J* = 9.1 Hz, 2H, Ar), 5.22–5.20 (m, 1H, CH), 3.70 (s, 3H, CH_3_O), 2.50–2.64 (m, 3H, CH_2_), 2.03–1.97 (m, 1H, CH_2_). ^13^C NMR (CDCl_3_, 150 MHz): δ 174.6 (CO), 157.2 (C), 149.4 (CH), 148.4 (CH), 136.9 (C), 133.8 (CH), 130.4 (C), 124.6 (2 × CH), 123.9 (CH), 114.2 (2 × CH), 62.0 (CH), 55.4 (CH_3_O), 31.0 (CH_2_), 28.9 (CH_2_). IR (KBr, cm^−1^): 3309, 3128, 3041, 3000, 2953, 2934, 2911, 2834, 1672, 1607, 1547, 1509, 1463, 1441, 1431, 1409, 1385, 1296, 1243, 1178, 1107, 1028. HRMS ESI-TOF: *m*/*z* = 269.1285 [M + H]^+^ (269.1285 calcd. for C_16_H_17_N_2_O_2_^+^). Spectral data are consistent with the reported ones [15].

*1-Benzyl-5-(3,4,5-trimethoxyphenyl)pyrrolidin-2-one (***3a**) To a solution of dimethyl 2-(3,4,5-trimethoxyphenyl)cyclopropane-1,1-dicarboxylate (550 mg, 1.70 mmol) in DCE (8.5 mL) in the presence of molecular sieves, 4 Å Ni(ClO_4_)_2_∙6H_2_O (63.4 mg, 0.17 mmol) was added; then, benzylamine (222 μL, 2.03 mmol) was added. The resulting mixture was refluxed for 1.5 h, diluted with DCM, and filtered through a small pad of silica gel using EtOAc as the eluent. Then, solvent was removed under vacuum; residue was dissolved in ethanol (9.8 mL), and aq. solution of NaOH (136 mg, 3.40 mmol; 3.5 mL) was added in one portion. The reaction mixture was stirred at room temperature for 2 h, and after that, ethanol was removed under vacuum. The residue was diluted with water, and 1 M HCl was added until pH 1. Then, mixture was extracted with ethyl acetate (3 × 10 mL). The combined organic layers were dried with Na_2_SO_4_ and concentrated in vacuo. The residue was dissolved in toluene (26.1 mL) and refluxed for 7 h. The solvent was removed under vacuum; pure product was isolated by column chromatography on silica gel. Yield: 393 mg (68%); yellowish solid; mp 99–102 °C; *R_f_* = 0.69 (ethyl acetate). ^1^H NMR (CDCl_3_, 600 MHz): δ 7.27–7.21 (m, 3H, Ar), 7.08–7.07 (m, 2H, Ar), 6.29 (s, 2H, Ar), 5.01 (d, ^2^*J* = 14.5 Hz, 1H, CH_2_), 4.32 (dd, ^3^*J* = 7.9 Hz, ^3^*J* = 6.1 Hz, 1H, CH), 3.84 (s, 3H, CH_3_O), 3.79 (s, 6H, 2 × CH_3_O), 3.62 (d, ^2^*J* = 14.5 Hz, 1H, CH_2_), 2.65–2.60 (m, 1H, CH_2_), 2.51–2.45 (m, 1H, CH_2_), 2.41–2.35 (m, 1H, CH_2_), 1.91–1.85 (m, 1H, CH_2_). ^13^C NMR (CDCl_3_, 150 MHz): δ 175.4 (CO), 153.7 (2 × C), 137.6 (C), 136.5 (C), 136.4 (C), 128.6 (2 × CH), 128.5 (2 × CH), 127.5 (CH), 103.5 (2 × CH), 61.9 (CH), 60.9 (CH_3_O), 56.2 (2 × CH_3_O), 44.6 (CH_2_), 30.4 (CH_2_), 28.3 (CH_2_). IR (KBr, cm^−1^): 2926, 1672, 1594, 1247, 1117, 1008, 700. HRMS ESI-TOF: *m*/*z* = 342.1700 [M + H]^+^ (342.1700 calcd. for C_20_H_24_NO_4_^+^).

*1-(4-Methoxybenzyl)-5-(3,4,5-trimethoxyphenyl)pyrrolidin-2-one* (**3b**) [39] was obtained from dimethyl 2-(3,4,5-trimethoxyphenyl)cyclopropane-1,1-dicarboxylate (550 mg, 1.70 mmol), (4-methoxyphenyl)methanamine (285 μL, 2.18 mmol), Ni(ClO_4_)_2_∙6H_2_O (63.0 mg, 0.17 mmol), DCE (8.5 mL), NaOH (128 mg, 3.20 mmol), ethanol (9.0 mL), and water (3.2 mL) according to the general procedure **2b**. Yield: 440 mg (70%); light yellow oil; *R_f_* = 0.66 (ethyl acetate).^1^H NMR (CDCl_3_, 600 MHz): δ 6.96 (d, ^3^*J* = 8.6 Hz, 2H, Ar), 6.75 (d, ^3^*J* = 8.6 Hz, 2H, Ar), 6.27 (s, 2H, Ar), 4.92 (d, ^2^*J* = 14.5 Hz, 1H, CH_2_), 4.27 (dd, ^3^*J* = 8.2 Hz, ^3^*J* = 6.4 Hz, 1H, CH), 3.81 (s, 3H, CH_3_O), 3.77 (s, 6H, 2 × CH_3_O), 3.72 (s, 3H, CH_3_O), 3.52 (d, ^2^*J* = 14.5 Hz, 1H, CH_2_), 2.60–2.55 (m, 1H, CH_2_), 2.45–2.40 (m, 1H, CH_2_), 2.36–2.30 (m, 1H, CH_2_), 1.86–1.80 (m, 1H, CH_2_). ^13^C NMR (CDCl_3_, 150 MHz): δ 175.3 (CO), 158.9 (C), 153.6 (2 × C), 137.5 (C), 136.4 (C), 129.8 (2 × CH), 128.4 (C), 113.7 (2 × CH), 103.5 (2 × CH), 61.8 (CH), 60.8 (CH_3_O), 56.1 (2 × CH_3_O), 55.2 (CH_3_O), 43.9 (CH_2_), 30.4 (CH_2_), 28.2 (CH_2_). Spectral data are consistent with the reported ones [39].

*1-Benzyl-5-(2,4-dimethoxyphenyl)pyrrolidin-2-one (***3c**) was obtained from dimethyl 2-(2,4-dimethoxyphenyl)cyclopropane-1,1-dicarboxylate (125 mg, 0.425 mmol), benzylamine (0.056 mL, 0.51 mmol), Ni(ClO_4_)_2_∙6H_2_O (16 mg, 0.044 mmol), and NaCl (37 mg, 0.64 mmol) in DCM (0.5 mL) according to the general procedure **2** (1 h for the first step and 4 h for the second step). Yield: 66 mg (50%); yellowish oil, *R_f_* = 0.47 (ethyl acetate:petroleum ether; 2:1). ^1^H NMR (CDCl_3_, 400 MHz): δ 7.22–7.29 (m, 3H, Ar), 7.08–7.13 (m, 2H, Ar), 6.91–6.95 (m, 1H, Ar), 6.43–6.49 (m, 2H, Ar), 5.05 (d, ^2^*J* = 14.7 Hz, 1H, CH_2_), 4.73 (dd, ^3^*J* = 8.5 Hz, ^3^*J* = 4.2 Hz, 1H, CH), 3.82 (s, 3H, CH_3_O), 3.71 (s, 3H, CH_3_O), 3.54 (d, ^2^*J* = 14.7 Hz, 1H, CH_2_), 2.54–2.64 (m, 1H, CH_2_), 2.40–2.49 (m, 1H, CH_2_), 2.27–2.38 (m, 1H, CH_2_), 1.83–1.94 (m, 1H, CH_2_). ^13^C NMR (CDCl_3_, 100 MHz): δ 175.5 (CO), 160.5 (C), 158.2 (C), 136.7 (C), 128.3 (4 × CH), 128.0 (CH), 127.2 (CH), 120.6 (C), 104.0 (CH), 98.8 (CH), 56.1 (CH), 55.32 (CH_3_O), 55.21 (CH_3_O), 44.2 (*C*H_2_Ph), 30.2 (C(4)H_2_), 26.3 (C(3)H_2_). IR (film, cm^−1^): 3086, 3064, 3030, 3001, 2940, 2837, 1739, 1685, 1612, 1588, 1507, 1456, 1441, 1416, 1358, 1292, 1277, 1262, 1209, 1158, 1119, 1033. HRMS ESI-TOF: *m*/*z* = 312.1599 [M + H]^+^ (312.1594 calcd. for C_19_H_22_NO_3_^+^). Spectral data are consistent with the reported ones [77].

*1-(2,5-Dimethoxybenzyl)-5-(3,4-dimethoxyphenyl)pyrrolidin-2-on* (**3d**) was obtained from dimethyl 2-(3,4-dimethoxyphenyl)cyclopropane-1,1-dicarboxylate (300 mg, 1.02 mmol), 2,5-dimethoxybenzylamine (0.184 mL, 1.22 mmol), Ni(ClO_4_)_2_∙6H_2_O (37 mg, 0.101 mmol), and NaCl (85 mg, 1.5 mmol) in DCM (0.5 mL) according to the general procedure **2** (1 h for the first step and 6 h for the second step). Yield: 178 mg (47%); yellow oil, *R_f_* = 0.49 (ethyl acetate:petroleum ether; 3:1). ^1^H NMR (CDCl_3_, 500 MHz): δ 6.81 (d, ^3^*J* = 8.2 Hz, 1H, Ar), 6.73 (dd, ^3^*J* = 8.8 Hz, ^4^*J* = 2.9 Hz, 1H, Ar), 6.70 (d, ^3^*J* = 8.8 Hz, 1H, Ar), 6.66 (dd, ^3^*J* = 8.2 Hz, ^4^*J* = 2.0 Hz, 1H, Ar), 6.63 (d, ^4^*J* = 2.9 Hz, 1H, Ar), 6.57 (d, ^4^*J* = 2.0 Hz, 1H, Ar), 4.85 (d, ^2^*J* = 14.8 Hz, 1H, CH_2_), 4.42 (dd, ^3^*J* = 8.1 Hz, ^3^*J* = 5.8 Hz, 1H, CH), 3.86 (s, 3H, CH_3_O), 3.81 (s, 3H, CH_3_O), 3.78 (d, ^2^*J* = 14.8 Hz, 1H, CH_2_), 3.70 (s, 3H, CH_3_O), 3.61 (s, 3H, CH_3_O), 2.57–2.64 (m, 1H, CH_2_), 2.42–2.50 (m, 1H, CH_2_), 2.35–2.45 (m, 1H, CH_2_), 1.82–1.90 (m, 1H, CH_2_). ^13^C NMR (CDCl_3_, 125 MHz): δ 175.3 (CO), 153.3 (C), 151.6 (C), 149.2 (C), 148.5 (C), 133.7 (C), 125.5 (C), 118.8 (CH), 115.8 (CH), 112.8 (CH), 111.1 (CH), 111.0 (CH), 109.3 (CH), 61.7 (CH), 55.8 (CH_3_O), 55.7 (CH_3_O), 55.59 (CH_3_O), 55.53 (CH_3_O), 39.4 (*C*H_2_Ar), 30.1 (C(4)H_2_), 28.3 (C(3)H_2_). IR (film, cm^−1^): 3074, 2998, 2940, 2912, 2835, 2251, 2063, 1693, 1608, 1593, 1518, 1500, 1465, 1412, 1357, 1315, 1303, 1278, 1260, 1218, 1154, 1138, 1120, 1046, 1026. HRMS ESI-TOF: *m*/*z* = 372.1817 [M + H]^+^ (372.1805 calcd. for C_21_H_26_NO_5_^+^).

*1-(Furan-2-ylmethyl)-5-(2,4,5-trimethoxyphenyl)pyrrolidin-2-one* (**3e**) was obtained from dimethyl 2-(2,4,5-trimethoxyphenyl)cyclopropane-1,1-dicarboxylate (400 mg, 1.23 mmol), furfurylamine (114 μL, 1.29 mmol), Ni(ClO_4_)_2_∙6H_2_O (90.2 mg, 0.25 mmol), DCE (6.2 mL), AcOH (140 μL), toluene (10.7 mL), NaOH (41.3 mg, 1.03 mmol), ethanol (3 mL), and water (1 mL) according to the general procedure **1**. Yield: 132 mg (32%); light yellow thick oil, *R_f_* = 0.18 (ethyl acetate:petroleum ether; 1:1). ^1^H NMR (CDCl_3_, 600 MHz) δ 7.28–7.26 (m, 1H, Fu), 6.53 (s, 1H, Ar), 6.51 (s, 1H, Ar), 6.22 (dd, ^3^*J* = 3.0 Hz, ^3^*J* = 1.7 Hz, 1H, Fu), 6.01 (br. d, ^3^*J* = 3.0 Hz, 1H, Fu), 4.86–4.83 (m, 2H, CH + CH_2_), 3.87 (s, 3H, CH_3_O), 3.77 (s, 3H, CH_3_O), 3.74 (s, 3H, CH_3_O), 3.69 (d, ^2^*J* = 15.4 Hz, 1H, CH_2_), 2.57–2.52 (m, 1H, CH_2_), 2.44–2.32 (m, 2H, CH_2_), 1.87–1.81 (m, 1H, CH_2_). ^13^C NMR (CDCl_3_, 150 MHz): δ 175.5 (CO), 151.7 (C), 150.3 (C), 149.4 (C), 143.3 (C), 142.1 (CH), 119.9 (C), 111.2 (CH), 110.2 (CH), 108.3 (CH), 98.0 (CH), 56.7 (CH_3_O), 56.4 (CH + CH_3_O), 56.2 (CH_3_O), 37.4 (CH_2_), 30.3 (CH_2_), 26.7 (CH_2_). IR (film, cm^−1^): 2992, 2935, 2836, 1687, 1684, 1611, 1513, 1463, 1456, 1440, 1411, 1399, 1348, 1317, 1276, 1208, 1163, 1116, 1079, 1032, 1012. HRMS (ESI/TOF): *m*/*z* = 331.1420 [M]^+^ (331.1414 calcd. for C_18_H_21_NO_5_^+^).

*1-[(1H-Indol-4-yl)methyl]-5-(3,4-dimethoxyphenyl)pyrrolidin-2-one* (**3f**) was obtained from dimethyl 2-(2,4-dimethoxyphenyl)cyclopropane-1,1-dicarboxylate (100 mg, 0.34 mmol), 4-aminomethylindole (60 mg, 0.41 mmol), Ni(ClO_4_)_2_∙6H_2_O (13 mg, 0.036 mmol) in DCM (0.2 mL), and NaCl (30 mg, 0.51 mmol) according to the general procedure **2** (1 h for the first step and 4 h for the second step). Yield: 50 mg (42%); yellow oil, *R_f_* = 0.35 (ethyl acetate:petroleum ether; 4:1). ^1^H NMR (CDCl_3_, 500 MHz): δ 8.56 (br. s, 1H, NH), 7.34–7.37 (m, 1H, Ar), 7.18–7.21 (m, 1H, Ar), 7.05–7.09 (m, 1H, Ar), 6.86–6.90 (m, 1H, Ar), 6.68–6.72 (m, 2H, Ar), 6.56–6.59 (m, 2H, Ar), 5.45 (d, ^2^*J* = 14.3 Hz, 1H, CH_2_), 4.25 (dd, ^3^*J* = 8.0 Hz, ^3^*J* = 5.8 Hz, 1H, CH), 3.93 (s, 3H, CH_3_O), 3.83 (s, 3H, CH_3_O), 3.81 (d, ^2^*J* = 14.3 Hz, 1H, CH_2_), 2.68 (ddd, ^2^*J* = 17.2 Hz, ^3^*J* = 9.9 Hz, ^3^*J* = 5.8 Hz, 1H, CH_2_), 2.51 (ddd, ^2^*J* = 17.2 Hz, ^3^*J* = 9.9, ^3^*J* = 7.2 Hz, 1H, CH_2_), 2.26–2.35 (m, 1H, CH_2_), 1.82–1.90 (m, 1H, CH_2_). ^13^C NMR (CDCl_3_, 125 MHz): δ 175.0 (CO), 149.3 (C), 148.6 (C), 135.9 (C), 135.6 (C), 127.8 (C), 127.0 (C), 124.3 (CH), 121.4 (CH), 120.7 (CH), 119.1 (CH), 111.2 (CH), 110.8 (CH), 109.7 (CH), 101.3 (CH), 61.1 (CH), 55.91 (CH_3_O), 55.85 (CH_3_O), 42.8 (*C*H_2_Ar), 30.5 (C(4)H_2_), 28.1 (C(3)H_2_). IR (film, cm^−1^): 3465, 3396, 3371, 3308, 3293, 3272, 3118, 2998, 2932, 2875, 2836, 2384, 1730, 1665, 1611, 1595, 1516, 1462, 1443, 1415, 1347, 1317, 1305, 1259, 1236, 1173, 1151, 1137, 1086, 1025. HRMS ESI-TOF: *m*/*z* = 351.1696 [M + H]^+^ (351.1703 calcd. for C_21_H_23_N_2_O_3_^+^).

*(E)-1-[2-(1H-Indol-3-yl)ethyl]-5-styrylpyrrolidin-2-one* (**3g**) was obtained from dimethyl (*E*)-2-styrylcyclopropane-1,1-dicarboxylate (200 mg, 0.77 mmol), tryptamine (123 mg, 0.77 mmol), Ni(ClO_4_)_2_∙6H_2_O (56 mg, 0.155 mmol), DCE (3.8 mL), AcOH (90 μL), toluene (5.3 mL), NaOH (63 mg, 1.58 mmol), ethanol (4.4 mL), and water (0.8 mL) according to the general procedure **1**. Yield: 28 mg (11%); brownish thick oil, *R_f_* = 0.31 (ethyl acetate). ^1^H NMR (CDCl_3_, 600 MHz): ^1^H NMR (CDCl_3_, 600 MHz): δ 8.23 (br. s, 1H, NH), 7.54 (d, ^3^*J* = 7.9 Hz, 1H, Ar), 7.37–7.32 (m, 5H, Ar), 7.30–7.26 (m, 1H, Ar), 7.19–7.16 (m, 1H, Ar), 7.03–7.00 (m, 2H, Ar), 6.34 (d, ^3^*J* = 15.6 Hz, 1H, CH=), 5.91 (dd, ^3^*J* = 15.6 Hz, ^3^*J* = 8.6 Hz, 1H, CH=), 4.01–3.97 (m, 1H, CH), 3.89–3.85 (m, 1H, CH_2_), 3.36–3.31 (m, 1H, CH_2_), 3.11–3.06 (m, 1H, CH_2_), 2.99–2.94 (m, 1H, CH_2_), 2.52–2.46 (m, 1H, CH_2_), 2.42–2.36 (m, 1H, CH_2_), 2.21–2.15 (m, 1H, CH_2_), 1.80–1.74 (m, 1H, CH_2_). ^13^C NMR (CDCl_3_, 150 MHz): δ 175.1 (CO), 136.3 (C), 135.9 (C), 133.3 (CH), 128.9 (CH), 128.7 (2 × CH), 128.2 (CH), 127.4 (C), 126.6 (2 × CH), 122.1 (CH), 122.0 (CH), 119.3 (CH), 118.8 (CH), 113.0 (C), 111.2 (CH), 61.8 (CH), 41.4 (CH_2_), 30.4 (CH_2_) 25.9 (CH_2_), 23.5 (CH_2_). IR (film, cm^−1^): 3210, 3180, 3110, 3079, 3057, 3028, 2962, 2929, 2878, 2853, 2723, 2601, 2545, 2385, 2350, 2253, 1956, 1883, 1804, 1666, 1548, 1493, 1452, 1417, 1368, 1340, 1312, 1274, 1264, 1230, 1181, 1160, 1143, 1126, 1102, 1071, 1052, 1029, 1008, 979, 905. HRMS ESI-TOF: *m*/*z* = 331.1805 [M + H]^+^ (331.1805 calcd. for C_22_H_23_N_2_O^+^).

*1-Cyclobutyl-5-(4-morpholinophenyl)pyrrolidin-2-one* (**3h**) was obtained from dimethyl 2-(4-morpholinophenyl)cyclopropane-1,1-dicarboxylate (300 mg, 0.94 mmol), cyclobutylamine (84 μL, 0.99 mmol) in DCE (0.5 mL), and NaCl (75 mg, 1.28 mmol) according to the general procedure **2** (2.5 h for the first step and 4 h for the second step). Yield: 142 mg (50%); yellow oil, *R_f_* = 0.71 (chloroform:methanol, 10:1). ^1^H NMR (CDCl_3_, 500 MHz): δ 7.09 (d, ^3^*J* = 8.6 Hz, 2H, Ar), 6.96–6.88 (m, 2H, Ar), 4.73 (dd, ^3^*J* = 8.3 Hz, ^3^*J* = 3.4 Hz, 1H, CH), 4.32–4.23 (m, 1H, CH), 3.90–3.83 (m, 4H, 2 × CH_2_), 3.20–3.13 (m, 4H, 2 × CH_2_), 2.58–2.59 (m, 1H, CH_2_), 2.46–2.39 (m, 1H, CH_2_), 2.38–2.33 (m, 1H, CH_2_), 2.32–2.25 (m, 1H, CH_2_), 2.15–2.07 (m, 1H, CH_2_), 1.97–1.88 (m, 1H, CH_2_), 1.82–1.74 (m, 2H, CH_2_), 1.56–1.45 (m, 2H, CH_2_). ^13^C NMR (CDCl_3_, 125 MHz): δ 175.3 (CO*),* 126.6 (2 × CH), 115.8 (2 × CH), 66.6 (2 × CH_2_), 60.49 (CHAr), 49.2 (2 × CH_2_), 47.7 (CHN), 30.2 (CH_2_), 29.0 (CH_2_), 28.18 (CH_2_), 28.14 (CH_2_), 15.4 (CH_2_), quaternary aromatic carbons not observed. HRMS ESI-TOF: *m*/*z* = 301.1904 [M + H]^+^ (301.1911 calcd. for C_18_H_25_N_2_O_2_^+^).

*5-(3,4-Dimethoxyphenyl)-1-(prop-2-yn-1-yl)pyrrolidin-2-one* (**3i**) was obtained from dimethyl 2-(3,4-dimethoxyphenyl)cyclopropane-1,1-dicarboxylate (300 mg, 1.02 mmol), propargylamine (78 μL, 1.22 mmol) in DCE (0.5 mL), and NaCl (70 mg, 1.2 mmol) according to the general procedure **2** (2.5 h for the first step and 4 h for the second step). Yield: 86 mg (33%); yellow oil, *R_f_* = 0.43 (ethyl acetate). ^1^H NMR (CDCl_3_, 400 MHz): δ 6.83 (d, ^3^*J* = 8.3 Hz, 1H, Ar), 6.77 (dd, ^3^*J* = 8.3 Hz, ^4^*J* = 1.9 Hz, 1H, Ar), 6.69 (d, ^4^*J* = 1.9 Hz, 1H, Ar), 4.73–4.69 (m, 1H, CH), 4.52 (dd, ^2^*J* = 17.4 Hz, ^4^*J* = 2.5 Hz, 1H, CH_2_), 3.85 (s, 3H, CH_3_O), 3.84 (s, 3H, CH_3_O), 3.22 (dd, ^2^*J* = 17.4 Hz, ^4^*J* = 1.7 Hz, 1H, CH_2_), 2.61–2.52 (m, 1H, CH_2_), 2.50–2.42 (m, 2H, CH_2_), 2.13 (dd, ^4^*J* = 2.5 Hz, ^4^*J* = 1.7 Hz, 1H, CH), 1.95–1.86 (m, 1H, CH_2_). ^13^C NMR (CDCl_3_, 100 MHz): δ 174.8 (CO), 149.4 (C), 148.9 (C), 132.5 (C), 119.3 (CH), 111.2 (CH), 109.3 (CH), 77.6 (C), 71.7 (CH), 61.2 (CH), 55.84 (CH_3_O), 55.80 (CH_3_O), 30.3 (CH_2_), 30.0 (CH_2_), 28.0 (CH_2_). IR (film, cm^−1^): 3250, 3073, 2998, 2957, 2939, 2837, 2585, 2468, 2280, 2117, 2030, 2846, 1693, 1607, 1594, 1519, 1465, 1465, 1422, 1371, 1347, 1306, 1237, 1179, 1139, 1066, 1026. HRMS ESI-TOF: *m*/*z* = 260.1292 [M + H]^+^ (260.1281 calcd. for C_15_H_18_NO_3_^+^).

*Dimethyl 2-[2-(3,5-dimethoxyphenyl)-2-(phenylamino)ethyl]malonate* (**4a**). To a solution of dimethyl 2-(3,5-dimethoxyphenyl)cyclopropane-1,1-dicarboxylate (300 mg, 1.02 mmol) in DCE (5.1 mL) in the presence of molecular sieves, 4 Å Ni(ClO_4_)_2_∙6H_2_O (75 mg, 0.206 mmol) was added under Ar atmosphere; then, aniline (0.1 mL, 1.10 mmol) was added. The resulting mixture was stirred at room temperature for 1 h, diluted with DCM, and filtered. The filtrate was concentrated under vacuum; pure product was isolated by column chromatography on silica gel. Yield: 363 mg (92%); yellow viscous oil; *R_f_* = 0.50 (ethyl acetate:petroleum ether; 1:3). ^1^H NMR (CDCl_3_, 600 MHz): δ 7.13–7.10 (m, 2H, Ar), 6.69–6.67 (m, 1H, Ar), 6.56 (d, ^3^*J* = 7.8 Hz, 2H, Ar), 6.52 (d, ^4^*J* = 2.2 Hz, 2H, Ar), 6.36–6.35 (m, 1H, Ar), 4.40–4.37 (m, 1H, CH), 4.23 (br.s, 1H, NH), 3.77 (s, 6H, 2 × CH_3_O), 3.76 (s, 3H, CH_3_O), 3.73 (s, 3H, CH_3_O), 3.60–3.58 (m, 1H, CH), 2.43–2.41 (m, 2H, CH_2_). ^13^C NMR (CDCl_3_, 150 MHz): δ 170.1 (*C*O_2_Me), 169.7 (*C*O_2_Me), 161.2 (2 × C), 146.9 (C), 145.3 (C), 129.2 (2 × CH), 117.7 (CH), 113.5 (2 × CH), 104.4 (2 × CH), 99.1 (CH), 56.6 (CH), 55.3 (2× CH_3_O), 52.8 (CH_3_O), 52.8 (CH_3_O), 49.3 (CH), 37.0 (CH_2_). IR (film, cm^−1^) 3386, 3088, 3051, 3003, 2953, 2839, 1748, 1732, 1602, 1506, 1460, 1433, 1347, 1313, 1290, 1277, 1260, 1234, 1205, 1156, 1120, 1064, 1020, 993. HRMS ESI-TOF: *m*/*z* = 410.1574 [M + Na]^+^ (410.1562 calcd. for C_21_H_25_NNaO_6_^+^).

*Dimethyl 2-{2-[(2-nitrophenyl)amino]-2-phenylethyl}malonate* (**4b**). To a solution of dimethyl 2-phenylcyclopropane-1,1-dicarboxylate (200 mg, 0.85 mmol) in DCE (4.3 mL) in the presence of molecular sieves, 4 Å Ni(ClO_4_)_2_∙6H_2_O (62 mg, 0.17 mmol) was added under Ar atmosphere; then, 2-nitroaniline (118 mg, 0.85 mmol) was added. The resulting mixture was refluxed for 2 h, diluted with DCM, and filtered. The filtrate was concentrated under vacuum; the pure product was isolated by column chromatography on silica gel. Yield: 226 mg (71%); yellow oil; *R_f_* = 0.47 (ethyl acetate:petroleum ether; 1:3). ^1^H NMR (CDCl_3_, 600 MHz): δ 8.45 (d, ^3^*J* = 6.9 Hz, 1H, NH), 8.17–8.16 (m, 1H, Ar), 7.38–7.29 (m, 6H, Ar), 6.73 (d, ^3^*J* = 8.7 Hz, 1H, Ar), 6.65–6.63 (m, 1H, Ar), 4.69–4.65 (m, 1H, CH), 3.77(s, 3H, CH_3_O), 3.71 (s, 3H, CH_3_O), 3.50 (t, ^3^*J* = 7.2 Hz, 1H, CH), 2.59–2.47 (m, 2H, CH_2_). ^13^C NMR (CDCl_3_, 150 MHz): δ 169.4 (*C*O_2_Me), 169.3 (*C*O_2_Me), 144.4 (C), 140.9 (C), 136.3 (CH), 132.7 (C), 129.3 (2 × CH), 128.2 (CH), 126.9 (CH), 126.4 (2 × CH), 116.2 (CH), 115.0 (CH), 55.8 (CH), 53.0 (CH_3_O), 53.0 (CH_3_O), 49.0 (CH), 37.0 (CH_2_). IR (film, cm^−1^): 3373, 3084, 3032, 3007, 2959, 2921, 2886, 2851, 1751, 1727, 1619, 1584, 1575, 1512, 1504, 1451, 1440, 1418, 1359, 1339, 1317, 1281, 1262, 1232, 1205, 1169, 1159, 1121, 1096, 1062, 1041, 1028, 1011, 985, 961. HRMS ESI-TOF: m/z = 373.1394 [M + H]^+^ (373.1394 calcd. for C_19_H_21_N_2_O_6_^+^).

*Dimethyl 2-{2-[(2-aminophenyl)amino]-2-(3,4-dimethoxyphenyl)ethyl}malonate* (**4c**). To a solution of dimethyl 2-(3,4-dimethoxyphenyl)cyclopropane-1,1-dicarboxylate (200 mg, 0.68 mmol) and *o*-phenylenediamine (74 mg, 0.68 mmol) in DCE (3.4 mL) in the presence of molecular sieves, 4 Å Y(OTf)_3_ (74 mg, 0.14 mmol) was added under Ar atmosphere. The resulting mixture was stirred at room temperature for 2.25 h, diluted with DCM, and filtered. The filtrate was concentrated under vacuum; pure product was isolated by column chromatography on silica gel. Yield: 150 mg (55%); viscous yellowish oil; *R_f_* = 0.52 (ethyl acetate:petroleum ether; 2:1). ^1^H NMR (CDCl_3_, 400 MHz): *δ* 6.87–6.83 (m, 2H, Ar), 6.80 (d, ^3^*J* = 8.1 Hz, 1H, Ar), 6.71–6.62 (m, 3H, Ar), 6.45–6.43 (m, 1H, Ar), 3.56 (dd, ^3^*J* = 8.0 Hz, 1H, ^3^*J* = 6.4 Hz, 1H, 1H, CH), 3.85 (s, 3H, CH_3_O), 3.84 (s, 3H, CH_3_O), 3.75 (s, 3H, CH_3_O), 3.71 (s, 3H, CH_3_O), 3.56 (dd, ^3^*J* = 7.4 Hz, ^3^*J* = 6.6 Hz, 1H, CH), 2.54–2.38 (m, 2H, CH_2_). Signals of NH_2_ groups were not observed. ^13^C NMR (CDCl_3_, 100 MHz): *δ* 170.1 (*C*O_2_Me), 169.7 (*C*O_2_Me), 149.1 (C), 148.2 (C), 136.0 (C), 134.7 (C), 134.4 (C), 120.4 (CH), 119.0 (CH), 118.3 (CH), 116.5 (CH), 113.6 (CH), 111.2 (CH), 109.4 (CH), 56.3 (CH), 55.8 (2 × CH_3_O), 52.7 (2 × CH_3_O), 49.3 (CH), 37.1 (CH_2_). IR (film, cm^−1^): 3400, 3350, 3002, 2953, 2837, 2254, 1738, 1729, 1598, 1512, 1453, 1437, 1343, 1263, 1237, 1142, 1053, 912. HRMS ESI-TOF: *m*/*z* = 403.1855 [M + H]^+^ (403.1864 calcd. for C_21_H_27_N_2_O_6_^+^).

*2-[2-Phenyl-2-(phenylamino)ethyl]malononitrile* (**4d**) To a solution of 2-phenylcyclopropane-1,1-dicarbonitrile (417 mg, 2.48 mmol) in DCE (12 mL) and aniline (0.26 mL, 2.88 mmol) in the presence of molecular sieves, 4Å Y(OTf)_3_ (265 mg, 0.49 mmol, 20 mol%) was added under Ar atmosphere. The reaction mixture was stirred at room temperature for 4 days. Then, the resulting mixture was poured into saturated aq. solution of NaHCO_3_ (12 mL) and extracted with CH_2_Cl_2_ (3 × 10 mL). The combined organic fractions were washed with saturated aq. solution of NaHCO_3_ (2 × 10 mL) and water (1 × 10 mL), dried with Na_2_SO_4_, and concentrated in vacuo. The resulting residue was purified by column chromatography on silica gel. Yield: 266 mg (41%); white solid; m.p. 149–151 °C; *R_f_* = 0.68 (ethyl acetate:petroleum ether; 1:3). ^1^H NMR (CDCl_3_, 600 MHz): δ 7.41–7.38 (m, 2H, Ph), 7.34–7.32 (m, 3H, Ph), 7.19–7.15 (m, 2H, Ph), 6.80–6.76 (m, 1H, Ph), 6.67 (d, ^3^*J* = 8.1 Hz, 2H, Ph), 4.72–4.68 (m, 1H, CH), 3.98–3.93 (m, 2H, CH, NH), 2.51–2.47 (m, 2H, CH_2_). ^13^C NMR (CDCl_3_, 150 MHz): δ 145.8 (C), 139.9 (C), 129.5 (2 × CH), 129.4 (2 × CH), 128.6 (CH), 126.1 (2 × CH), 119.3 (CH), 114.4 (2 × CH), 112.5 (2 × CN), 55.3 (CH), 38.4 (CH), 20.2 (CH_2_). IR (KBr, cm^−1^) 3379, 3057, 2882, 2257, 1601, 1506, 1453, 1428, 1311, 1258, 769, 754, 704. HRMS ESI-TOF: *m*/*z* = 262.1339 [M + H] (262.1339 calcd. for C_17_H_16_N_3_).

*2-{2-[(2-Bromo-4-methylphenyl)amino]-2-phenylethyl}malononitrile* (**4e**). To a solution of 2-phenylcyclopropane-1,1-dicarbonitrile (150 mg, 0.89 mmol) and 2-bromo-4-methylaniline (200 mg, 1.07 mmol) in DCE (4.5 mL) in the presence of molecular sieves, 4 Å Y(OTf)_3_ (96 mg, 0.18 mmol) was added under Ar atmosphere. The reaction mixture was stirred at room temperature for 3 days, poured into saturated aq. solution of NaHCO_3_, and extracted with CH_2_Cl_2_ (3 × 10 mL). The combined organic fractions were washed with saturated aq. solution of NaHCO_3_ (2 × 10 mL) and water (1 × 10 mL), dried with Na_2_SO_4_, and concentrated in vacuo. The resulting residue was purified by column chromatography on silica gel. Yield: 136 mg (43%); yellow viscous oil; *R_f_* = 0.40 (ethyl acetate:petroleum ether; 1:4). ^1^H NMR (CD_3_OD, 600 MHz): δ 7.34–7.29 (m, 4H, Ar) 7.24–7.21 (m, 1H, Ar), 6.84 (d, ^3^*J* = 7.9 Hz, 1H, Ar), 6.65 (dd, ^3^*J* = 7.9 Hz, ^4^*J* = 1.8 Hz, 1H, Ar), 6.59 (d, ^4^*J* = 1.8 Hz, 1H, Ar), 4.68 (dd, ^3^*J* = 10.6 Hz, ^3^*J* = 4.4 Hz, 1H, CH), 2.57 (dd, ^2^*J* = 14.2 Hz, ^3^*J* = 10.6 Hz, 1H, CH_2_), 2.45 (dd, ^2^*J* = 14.2 Hz, ^3^*J* = 4.4 Hz, 1H, CH_2_), 2.21 (s, 3H, CH_3_). Signals of CH and NH groups were not observed. ^13^C NMR (CD_3_OD, 150 MHz): δ 147.2 (CN), 142.2 (CN), 132.4 (CH), 129.9 (2 × CH), 128.9 (CH), 127.2 (2 × CH), 123.6 (C), 121.2 (CH), 121.0 (C), 115.2 (CH), 114.7 (C), 114.6 (C), 56.1 (CH), 38.5 (CH_2_), 17.6 (CH_3_). Signal of CH group was not observed. IR (film, cm^−1^) 3391, 2898, 2524, 2257, 1597, 1490, 1408, 1266, 1071, 835, 701. HRMS ESI-TOF: *m*/*z* = 354.0584 [M + H^+^] (354.0600 calcd. for C_18_H_17_Br^79^ N_3_^+^).

(*3aRS,5RS)-5-Phenyl-3,3a,4,5-tetrahydropyrrolo [1,2-a]quinolin-1(2H)-one* (**5**). (*E*)-1-Phenyl-5-styrylpyrrolidin-2-one (**2h**) (50 mg, 0.19 mmol) was dissolved in polyphosphoric acid (400 mg) in triple evacuated/N_2_-filled vial. The obtained mixture was stirred at 100 °C for 20 min. Then, the reaction mixture was cooled and quenched with saturated aq. NaHCO_3_ solution. The resulted mixture was extracted with ethyl acetate (3 × 7 mL). The combined organic phases were dried with Na_2_SO_4_. The solvent was removed under vacuum; the pure product was isolated by column chromatography on silica gel. Yield: 27 mg (54%); dark ivory solid; m.p. 174–176 °C; *R_f_* = 0.69 (ethyl acetate:petroleum ether; 1:1). ^1^H NMR (CDCl_3_, 600 MHz): δ 8.71 (d, ^3^*J* = 8.3 Hz, 1H, C(9)H), 7.35–7.32 (m, 2H, C(3′)H, C(5′)H), 7.28–7.26 (m, 1H, C(4′)H), 7.23–7.21 (m, 1H, C(8)H), 7.16 (br. d, ^3^*J* = 7.2 Hz, 2H, C(2′)H, C(6′)H), 6.91–6.87 (m, 1H, C(7)H), 6.77 (d, ^3^*J* = 7.9 Hz, 1H, C(6)H), 4.17 (dd, ^3^*J* = 12.4 Hz, ^3^*J* = 5.8 Hz, 1H, C(5)H), 4.14–4.10 (m, 1H, C(3a)H), 2.63 (ddd, ^2^*J* = 17.1 Hz, ^3^*J* = 10.8 Hz, ^3^*J* = 9.9 Hz, 1H, C(2)H_2_), 2.55 (ddd, ^2^*J* = 17.1 Hz, ^3^*J* = 9.7 Hz, ^3^*J* = 2.3 Hz, 1H, C(2)H_2_), 2.39 (ddd, ^2^*J* = 13.3 Hz, ^3^*J* = 5.8 Hz, ^3^*J* = 2.3 Hz, 1H, C(4)H_2_), 2.35–2.29 (m, 1H, C(3)H_2_), 1.97–1.93 (m, 1H, C(4)H_2_), 1.80–1.73 (m, 1H, C(3)H_2_). ^13^C NMR (CDCl_3_, 150 MHz): δ 173.9 (C(1)), 145.0 (C(1′)), 136.7 (C(9a)), 130.0 (C(6)H), 129.7 (C(5a)), 128.8 (C(2′)H, C(6′)H), 128.6 (C(3′)H, C(5′)H), 127.2 (C(8)H), 126.9 (C(4′)H), 123.9 (C(7)H), 119.1 (C(9)H), 57.9 (C(3a)H), 45.0 (C(5)H), 40.2 (C(4)H_2_), 32.1 (C(2)H_2_), 25.0 (C(3)H_2_). IR (KBr, cm^−1^): 3080, 3064, 3021, 2985, 2934, 2916, 2849, 1682, 1645, 1598, 1580, 1487, 1449, 1439, 1385, 1372, 1320, 1307, 1294, 1271, 1240, 1227, 1202, 1184, 1166, 1147, 1124, 1116, 1066, 1042, 1032, 1024. HRMS ESI-TOF: *m*/*z* = 264.1390 [M + H]^+^ (264.1383 calcd. for C_18_H_18_NO^+^).

## 4. Conclusions

In summary, we developed a convenient general method for the synthesis of substituted γ-lactams based on Lewis acid-catalyzed DA cyclopropane ring opening with primary amines as the key step. Various 1,5-disubstituted γ-lactams were synthesized in moderate to good yields in three or four steps, requiring only a single purification procedure. We also demonstrated the potential of our method in the synthesis of an optically pure γ-lactam derivative from optically active DA cyclopropane. Additionally, the presence of reactive functionalities at the C(1) and C(5) atoms of γ-lactams ensured the possibility for postmodifications of the obtained products to convert them into more complex azaheterocycles, such as benz[*g*]indolizidine derivatives.

## Figures and Tables

**Table 1:**
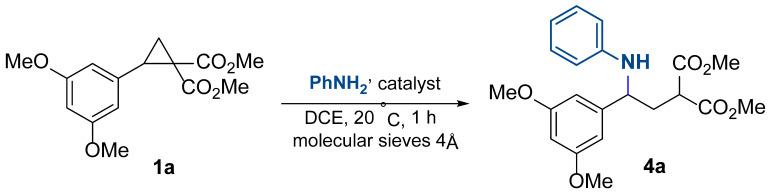
Optimization of reaction conditions for the model cyclopropane **1a** opening with aniline ^1^.

Entry	Catalyst	(mol%)	Yield of 4a, % ^2^
1	Al(OTf)_3_	20	-
2	Fe(OTf)_3_	20	78 ^3^
3	Sc(OTf)_3_	10	60 ^3,4^
4	Sc(OTf)_3_	20	84
5	Zn(OTf)_2_	20	80 ^3^
6	Y(OTf)_3_	20	93
7	Ni(ClO_4_)_2_·6H_2_O	5	21 ^3^
8	Ni(ClO_4_)_2_·6H_2_O	10	50 ^3^
9	Ni(ClO_4_)_2_·6H_2_O	20	92
10	TfOH	20	-

^1^ Concentration of **1a** was 0.2 M. ^2^ Isolated yield. ^3^ NMR yields (hexamethyldisiloxane was used as internal standard). ^4^ Concentration of **1a** was 0.03 M.

## Data Availability

Not applicable.

## Supplementary Materials

Copies of NMR spectra for novel compounds are available online. The following supporting information can be downloaded at: https://www.mdpi.com/article/10.3390/molecules27238468/s1.
