# Supplementary material for: Synthesis of 1,5-Substituted Pyrrolidin-2-ones from Donor–Acceptor Cyclopropanes and Anilines/Benzylamines
Source: Molecules. 2022 Dec 2;27(23):8468. doi: 10.3390/molecules27238468 (PMC9735934; doi:10.3390/molecules27238468)
Supplement: Supplementary file 1 [file molecules-27-08468-s001.zip › molecules-2031096-supplementary.pdf]

Synthesis of 1,5-Substituted Pyrrolidin-2-ones from Donor-Acceptor Cyclopropanes and Anilines/Benzylamines

Maksim A. Boichenko [1], Andrey Yu. Plodukhin [1], Vitaly V. Shorokhov [1], Danyla S. Lebedev [1], Anastasya V. Filippova [1], Sergey S. Zhokhov [1], Elena A. Tarasenko [1], Victor B. Rybakov [1], Igor V. Trushkov [2,*], and Olga A. Ivanova[1,*]

[1] Department of Chemistry, M. V. Lomonosov Moscow State University, Leninskie gory 1-3, Moscow 119991, Russia

[2] N. D. Zelinsky Institute of Organic Chemistry, Leninsky pr. 47, Moscow 119334, Russia

*Correspondence: iv@kinet.chem.msu.ru (O.A.I.); trush@ioc.ac.ru (I.V.T.); Tel.: +7-916-645-9951 (I.V.T.)

SUPPORTING INFORMATION

Table of Contents

^{1}H and ^{13}C NMR spectra	S2
HPLC determination of enantiomeric purity of pyrrolidin-2one (*R*)-**2d**	S65

5-(3,5-Dimethoxyphenyl)-1-phenylpyrrolidin-2-one (2a)

^{1}H NMR ($CDCl_3$, 600 MHz)

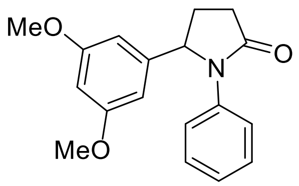

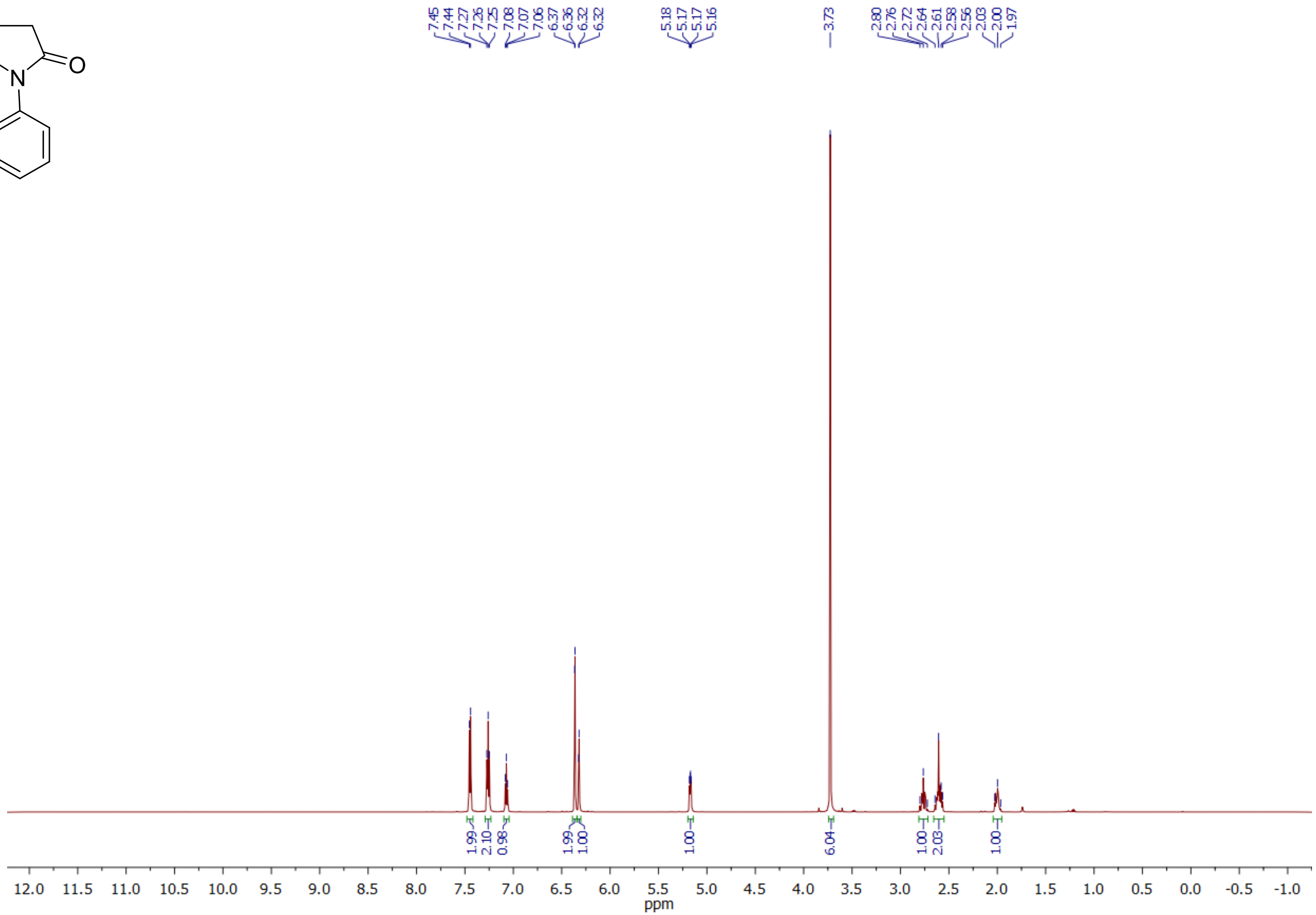

5-(3,5-Dimethoxyphenyl)-1-phenylpyrrolidin-2-one (**2a**)

^{13}C NMR ($CDCl_3$, 150 MHz)

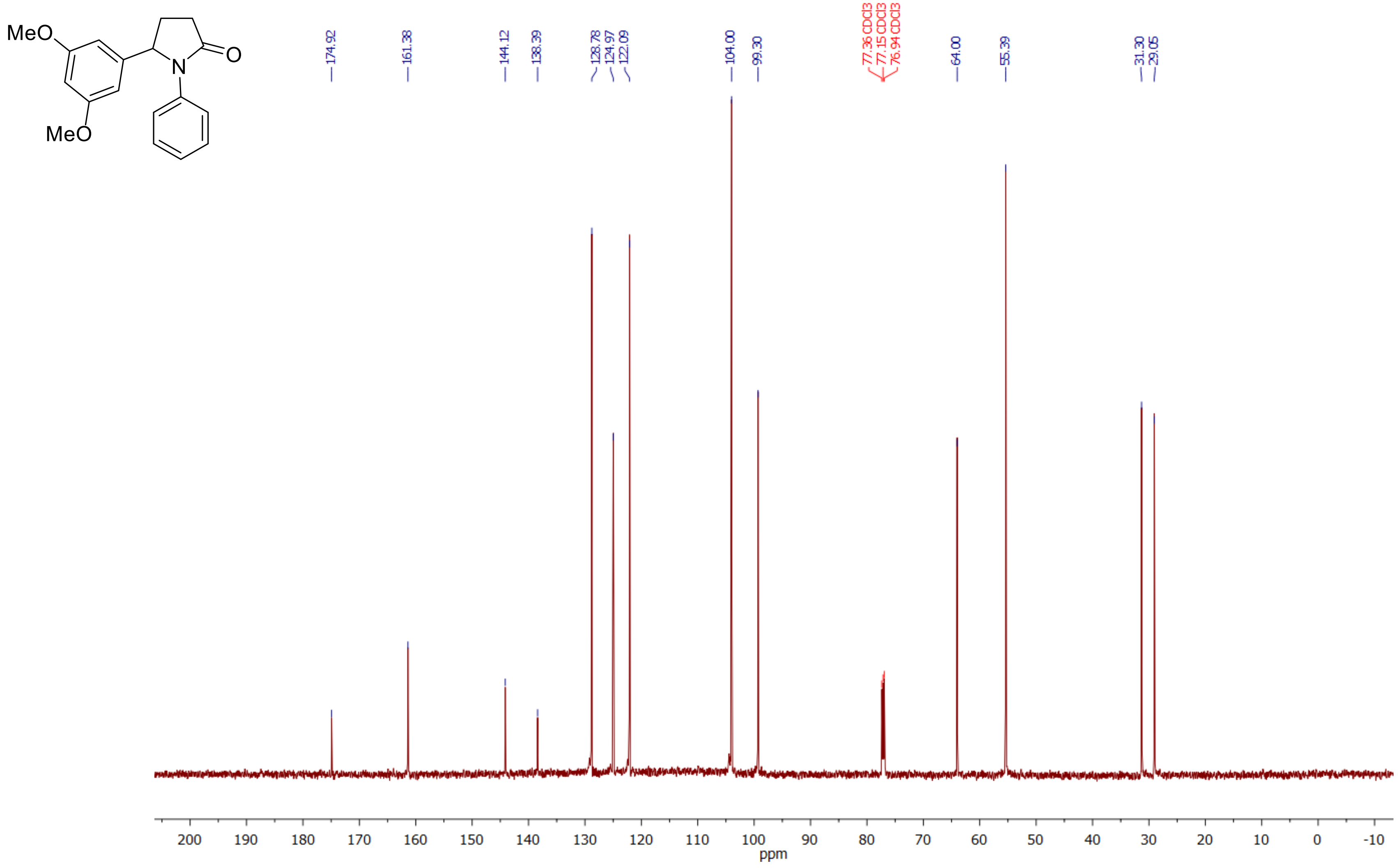

5-(3,4,5-Trimethoxyphenyl)-1-(2-fluorophenyl)pyrrolidin-2-one (2b)

^{1}H NMR ($CDCl_3$, 400 MHz)

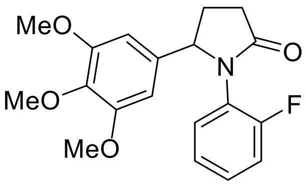

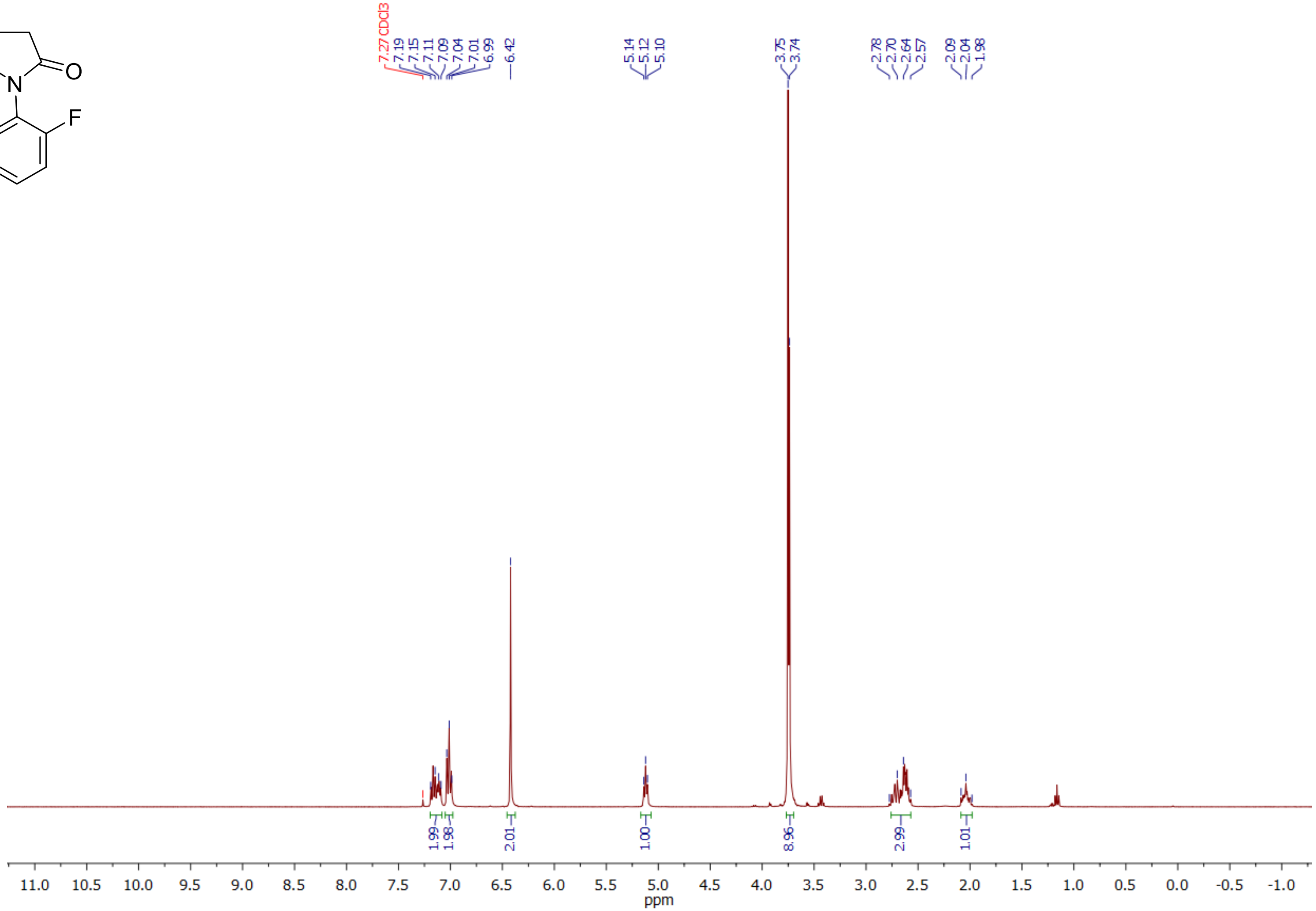

5-(3,4,5-Trimethoxyphenyl)-1-(2-fluorophenyl)pyrrolidin-2-one (2b)

^{13}C NMR ($CDCl_3$, 100 MHz)

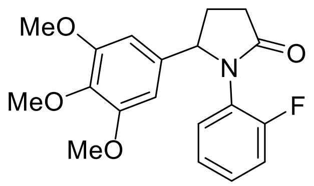

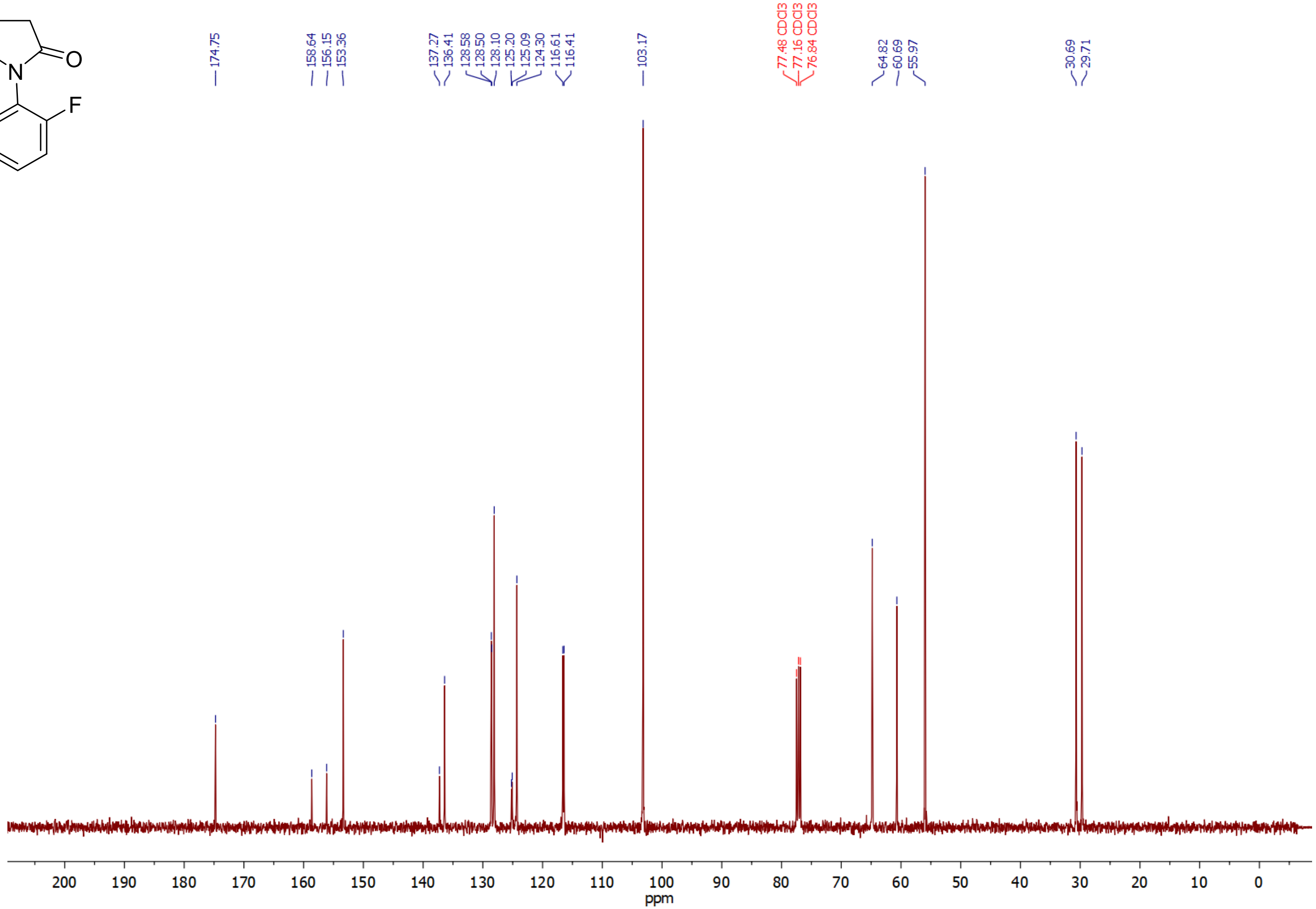

1-(4-Methoxyphenyl)-5-(*p*-tolyl)pyrrolidin-2-one (2d)

^{1}H NMR ($CDCl_3$, 600 MHz)

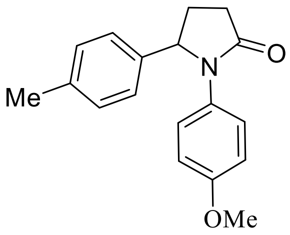

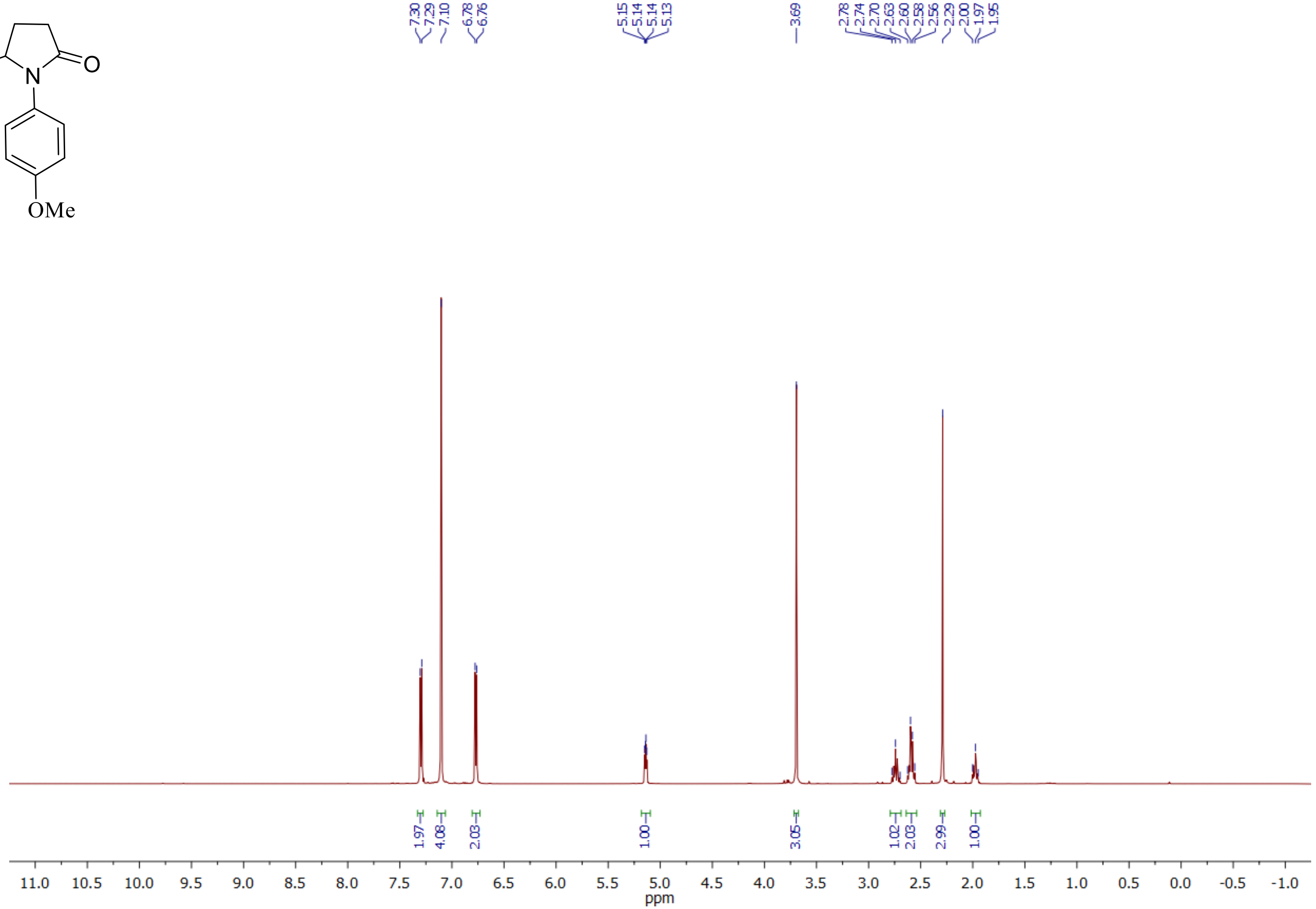

1-(4-Methoxyphenyl)-5-(*p*-tolyl)pyrrolidin-2-one (2d)

^{13}C NMR ($CDCl_3$, 150 MHz)

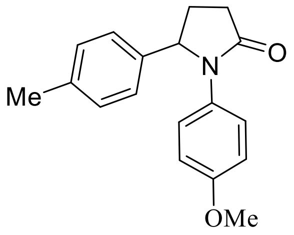

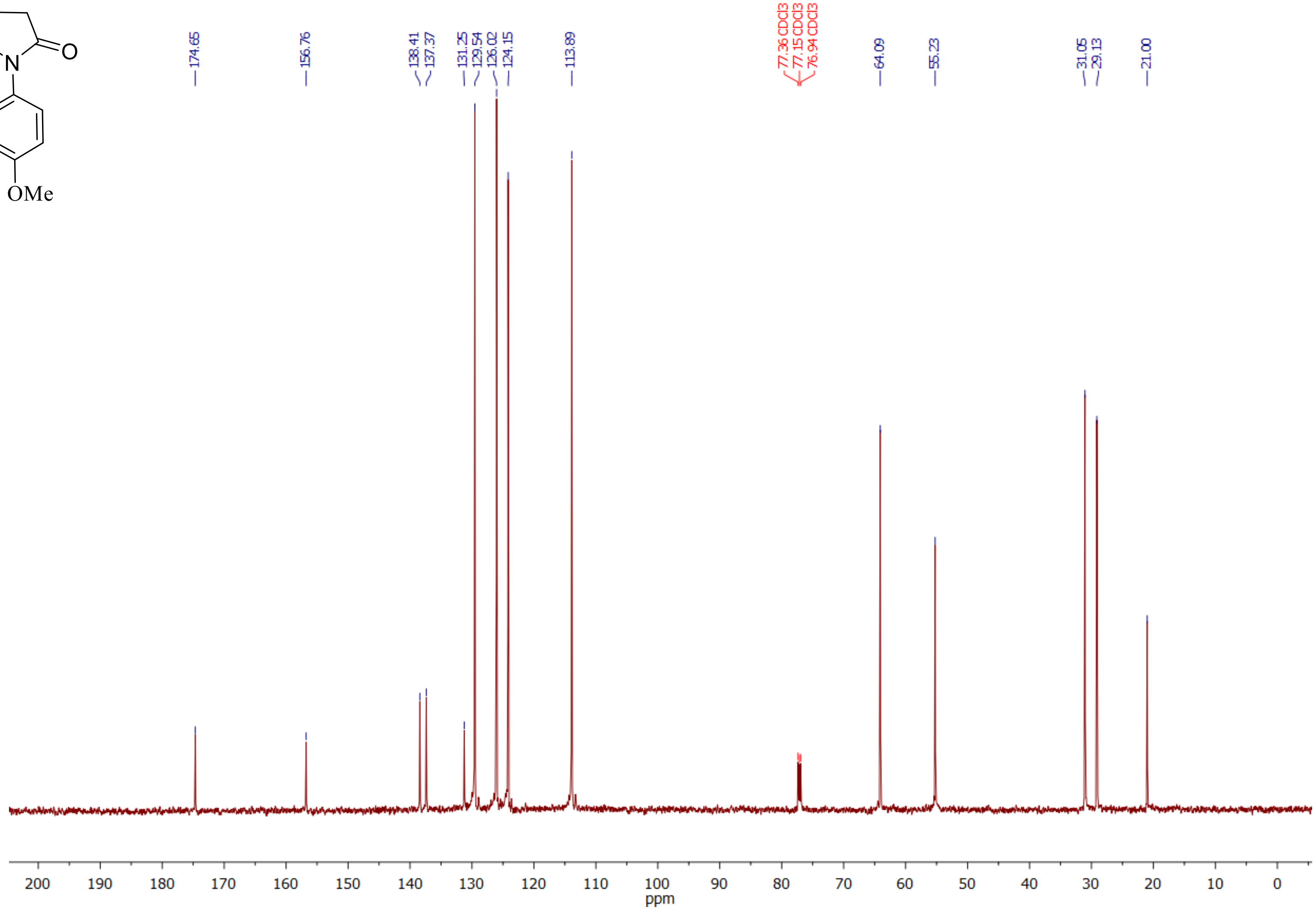

1-(4-Methoxyphenyl)-5-(p-tolyl)pyrrolidin-2-one (2d)

HSQC ^{1}H-^{13}C ($CDCl_3$)

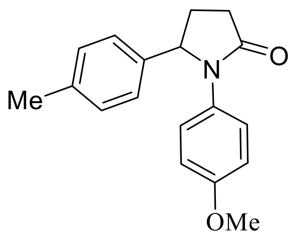

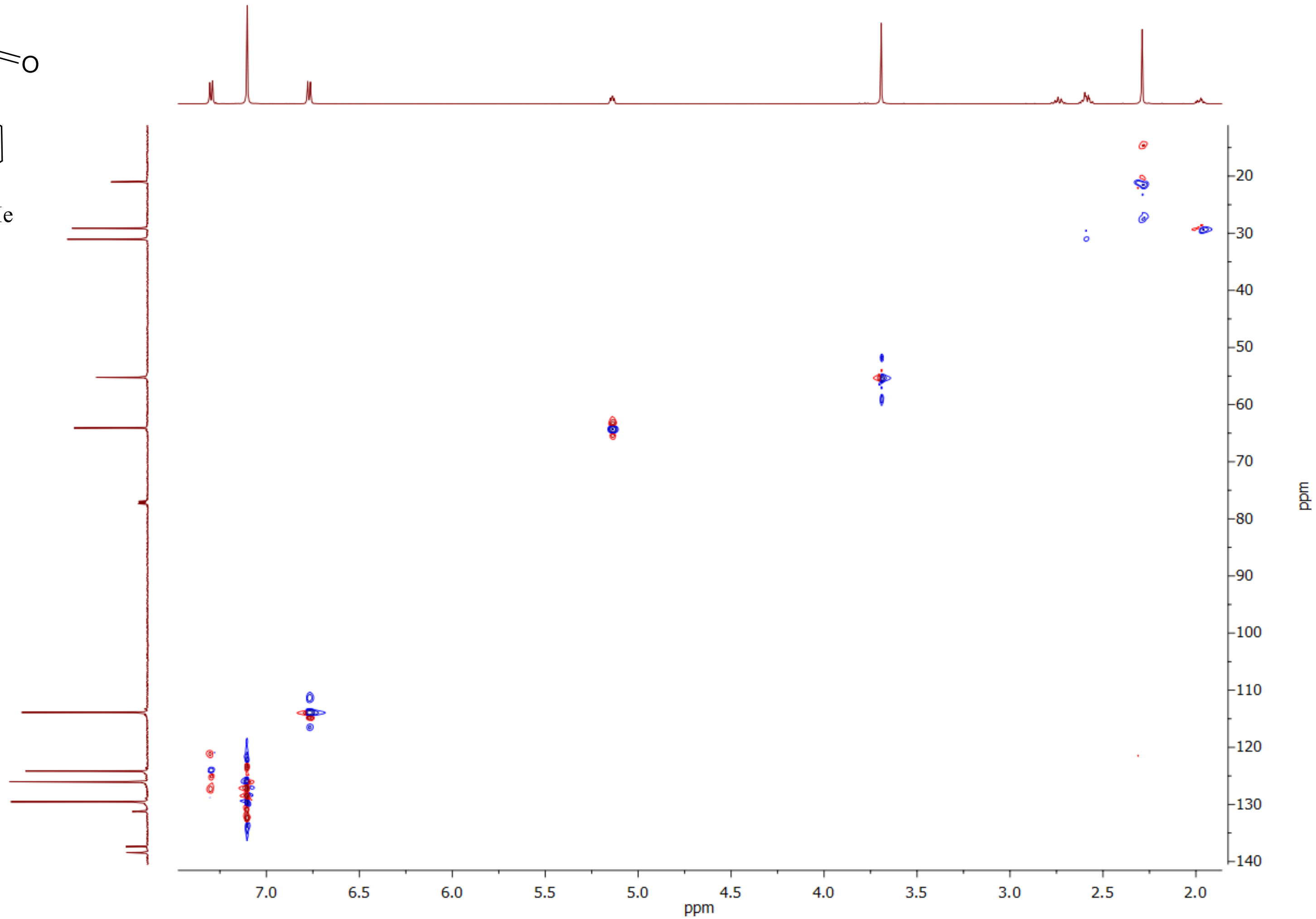

1-(4-Methoxyphenyl)-5-(*p*-tolyl)pyrrolidin-2-one (2d)

HMBC ^{1}H-^{13}C ($CDCl_3$)

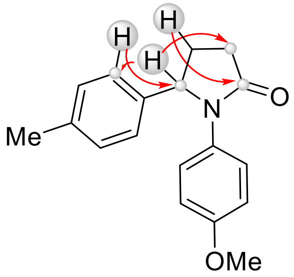

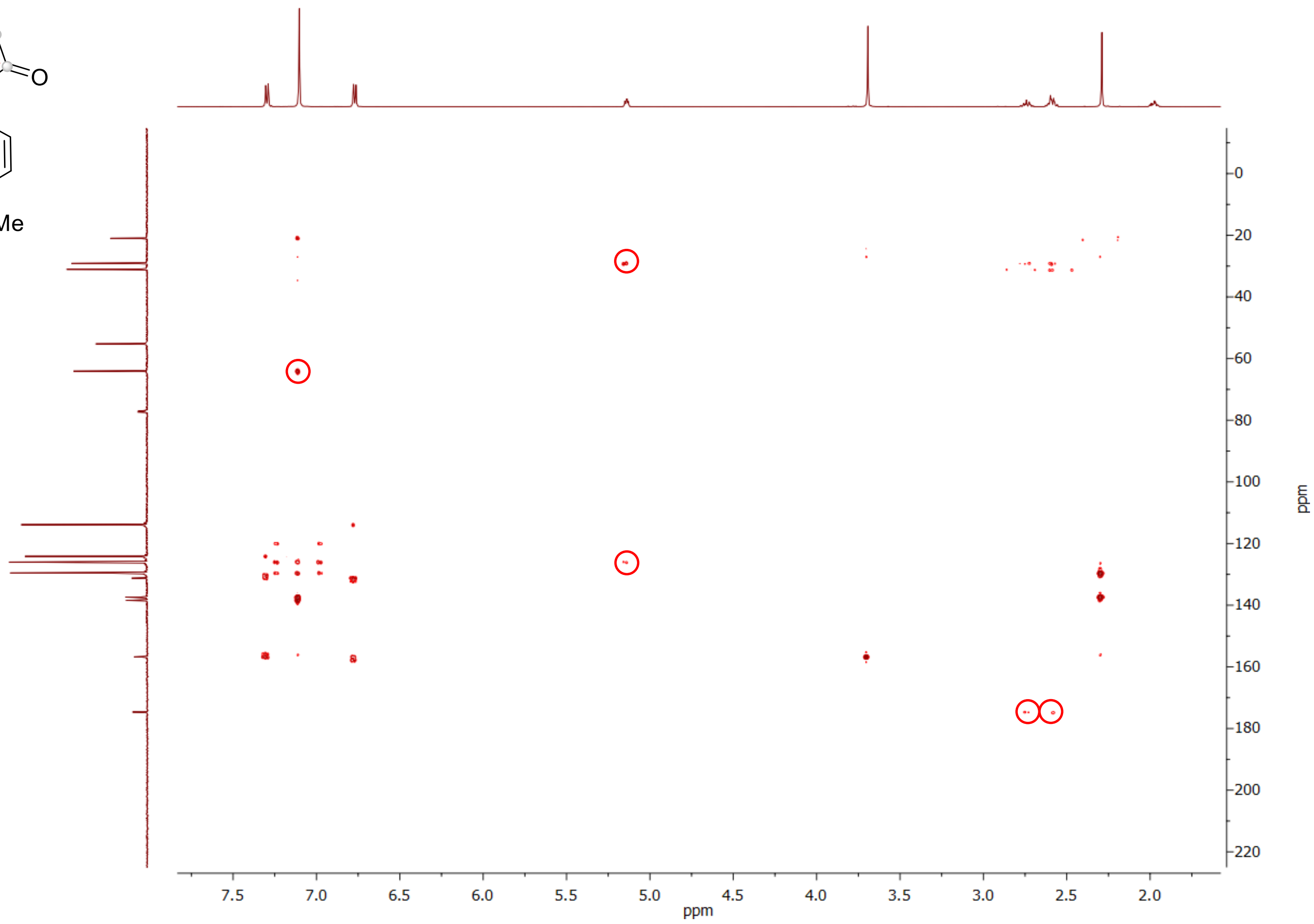

5-(3-Chlorophenyl)-1-(4-nitrophenyl)pyrrolidin-2-one (2e)

^{1}H NMR ($CDCl_3$, 600 MHz)

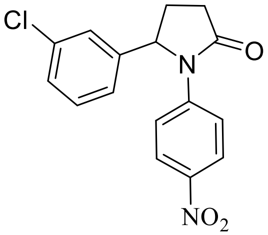

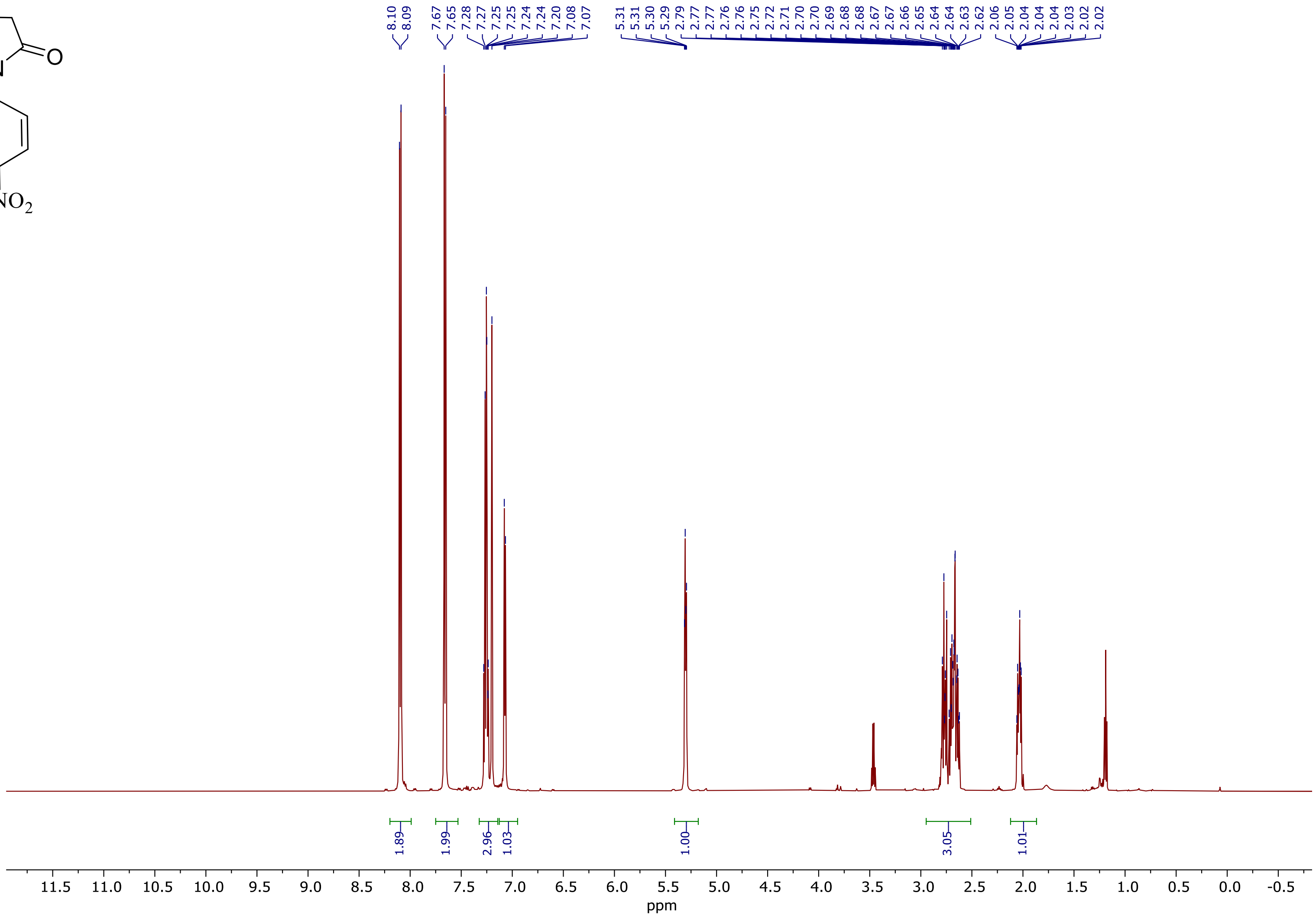

5-(3-Chlorophenyl)-1-(4-nitrophenyl)pyrrolidin-2-one (2e)

^{13}C NMR ($CDCl_3$, 150 MHz)

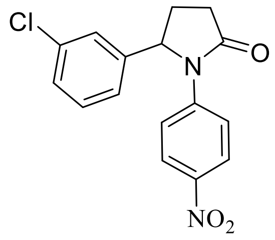

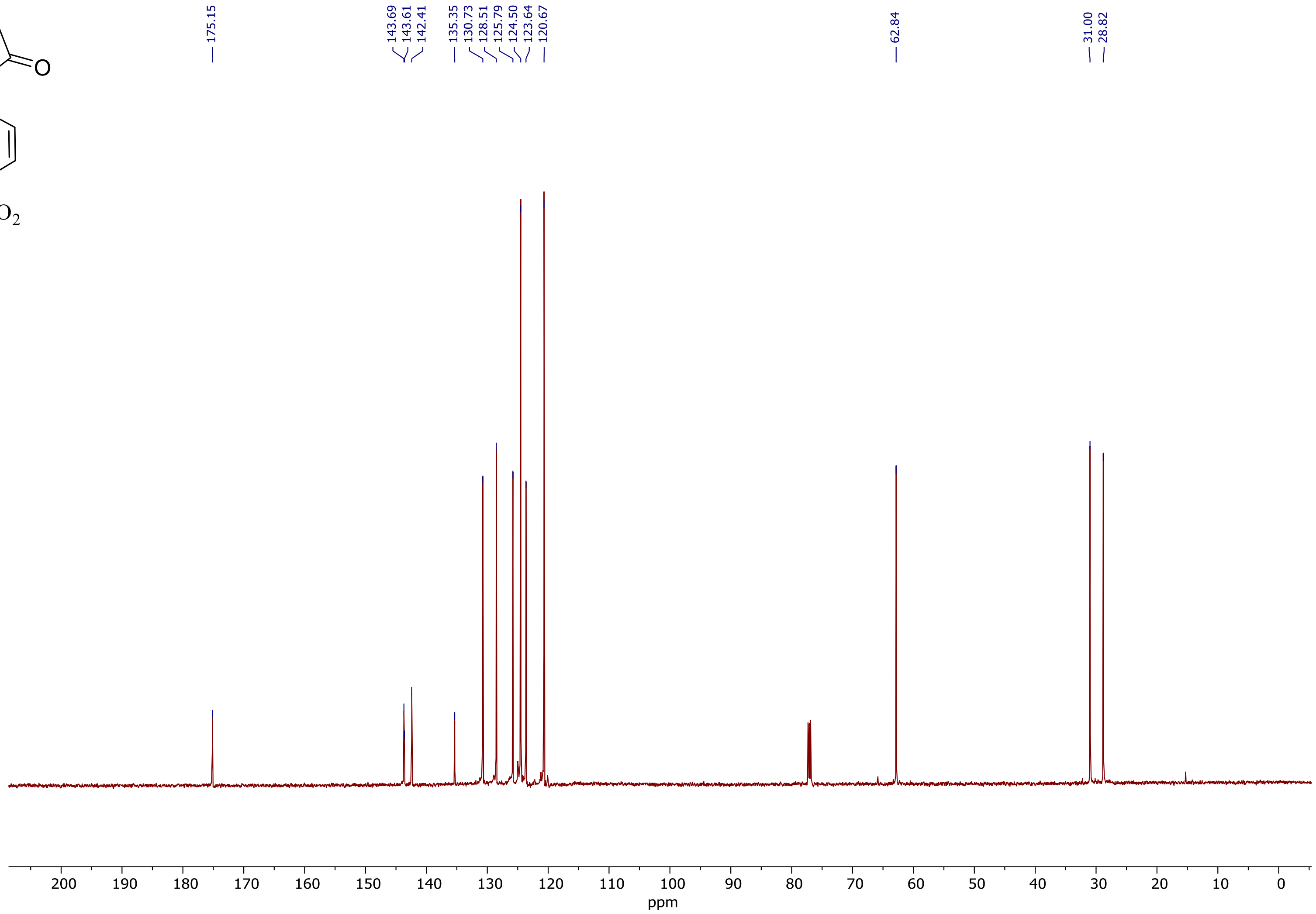

5-(3-Chlorophenyl)-1-(4-nitrophenyl)pyrrolidin-2-one (2e)

HSQC ^{1}H-^{13}C ($CDCl_3$)

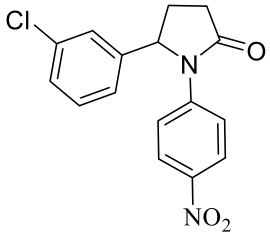

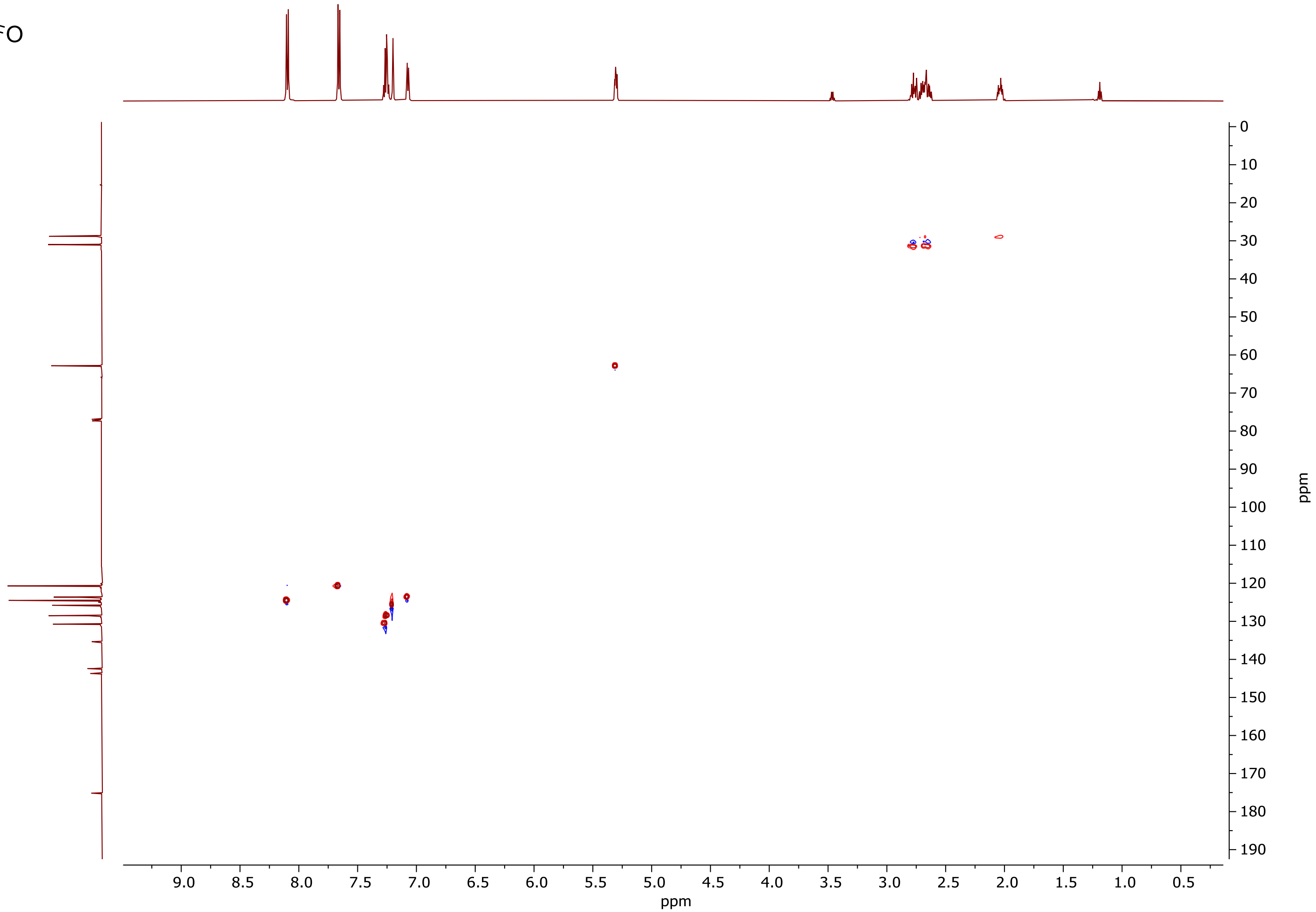

1-(3,4-Dimethoxyphenyl)-5-(5-methylfuran-2-yl)pyrrolidin-2-one (2f)

^{1}H NMR ($CDCl_3$, 600 MHz)

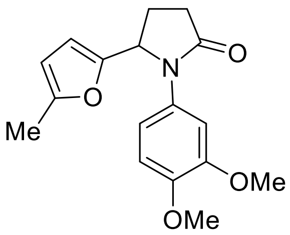

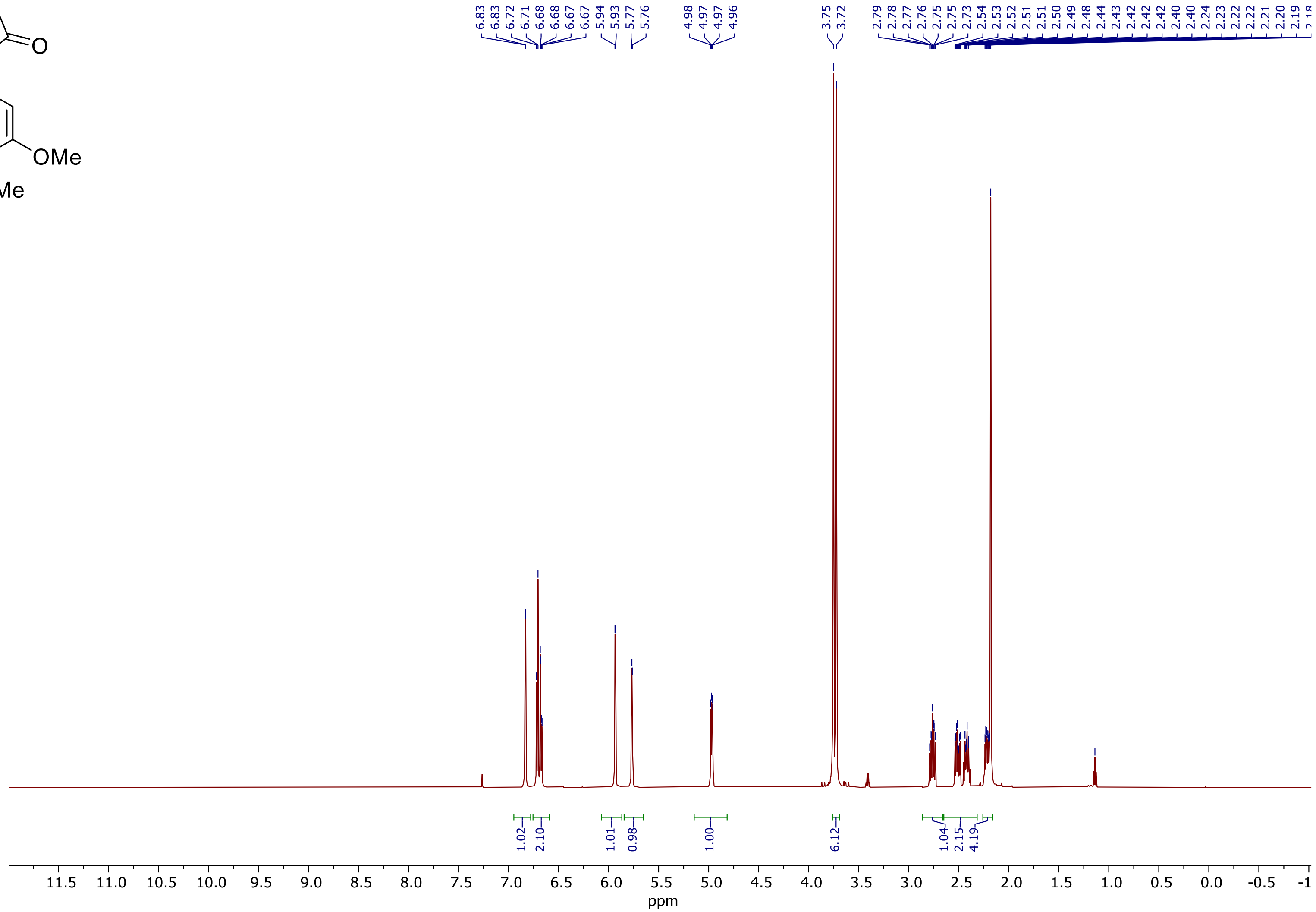

1-(3,4-Dimethoxyphenyl)-5-(5-methylfuran-2-yl)pyrrolidin-2-one (2f)

^{13}C NMR ($CDCl_3$, 150 MHz)

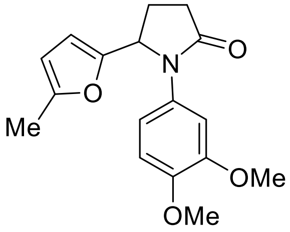

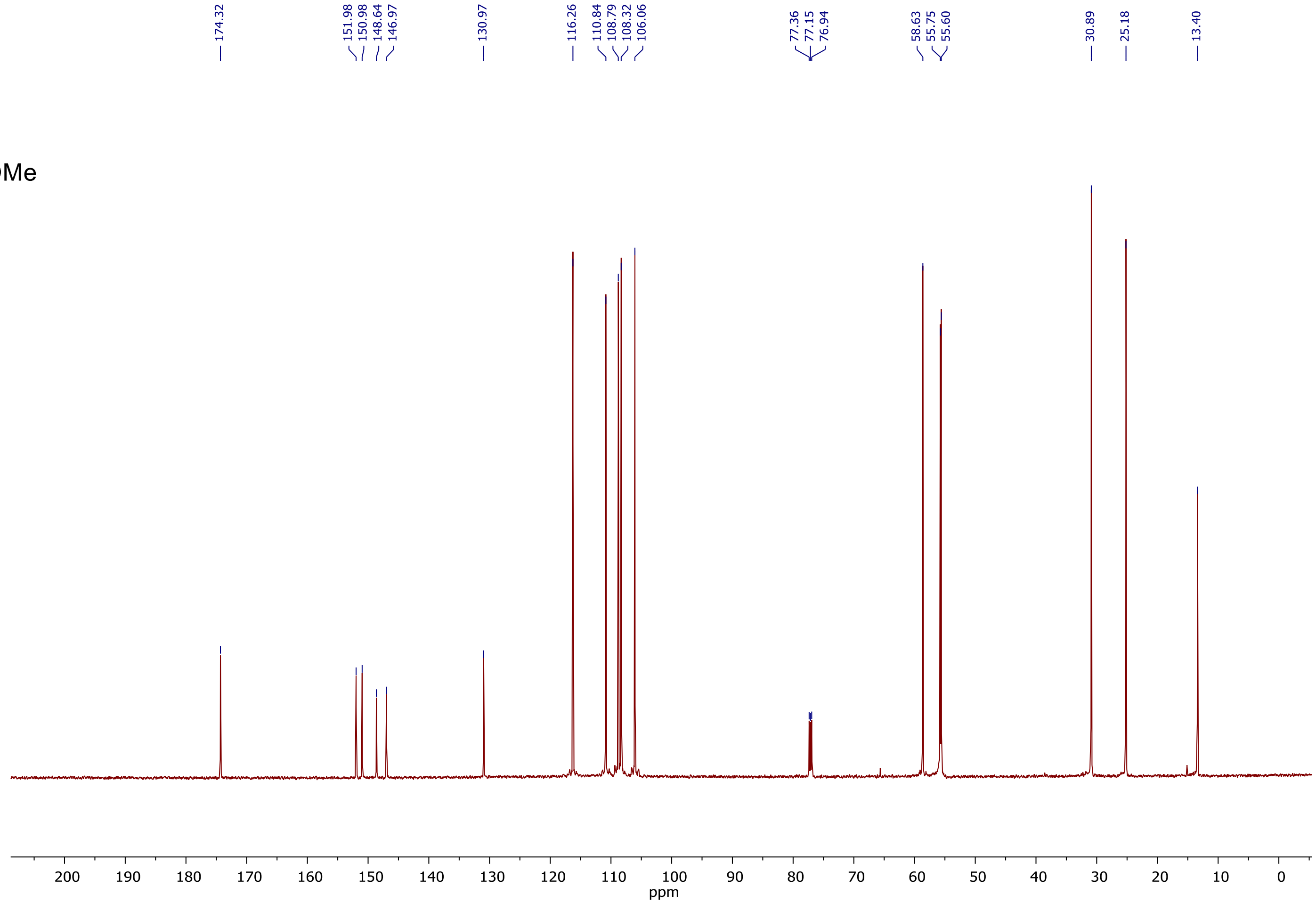

1-(3,4-Dimethoxyphenyl)-5-(5-methylfuran-2-yl)pyrrolidin-2-one (2f)

HSQC ^{1}H-^{13}C ($CDCl_3$)

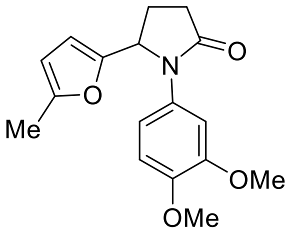

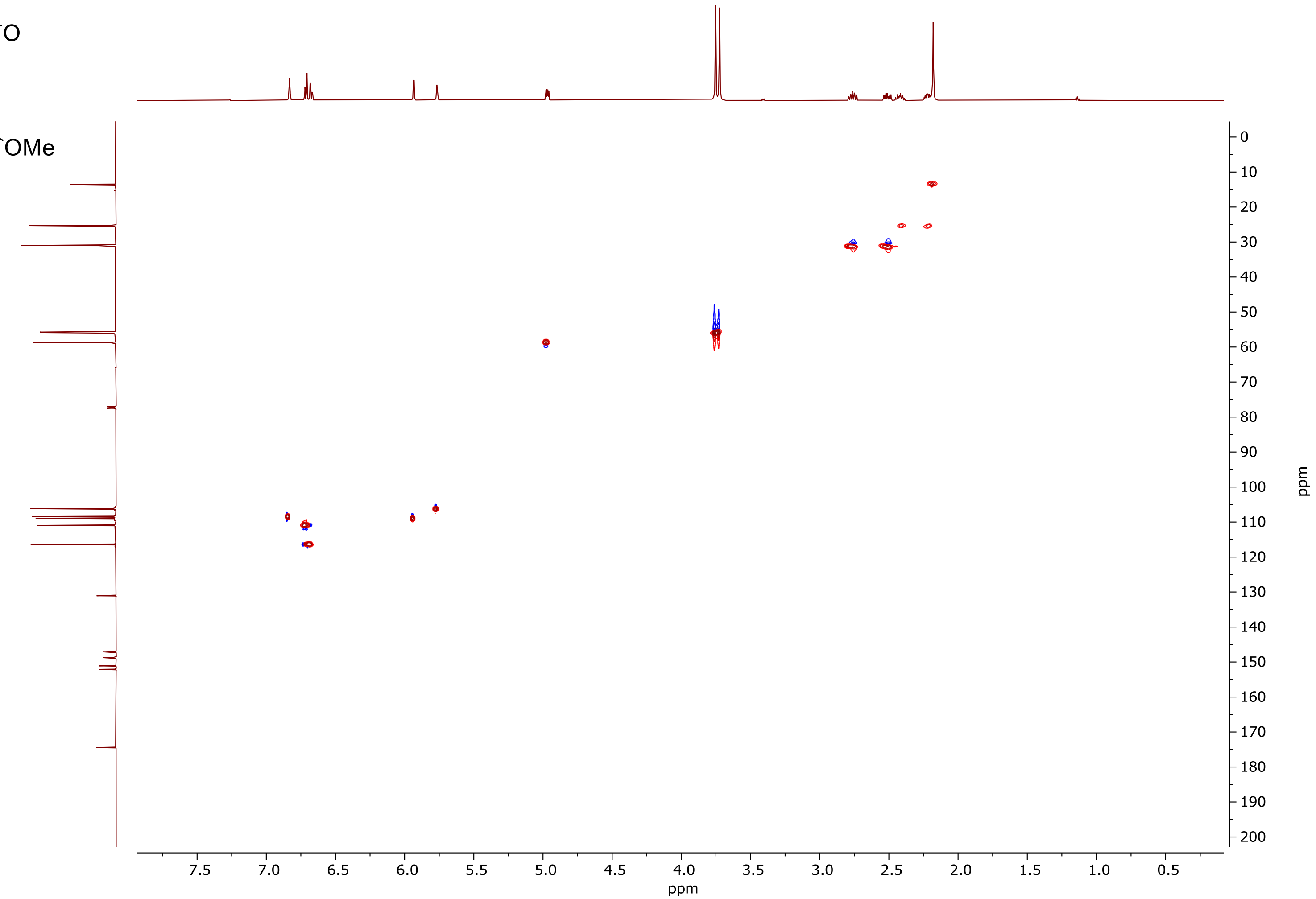

1-(3,4-Dimethoxyphenyl)-5-(5-methylfuran-2-yl)pyrrolidin-2-one (2f)

HMBC ^{1}H-^{13}C ($CDCl_3$)

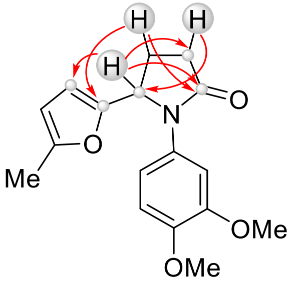

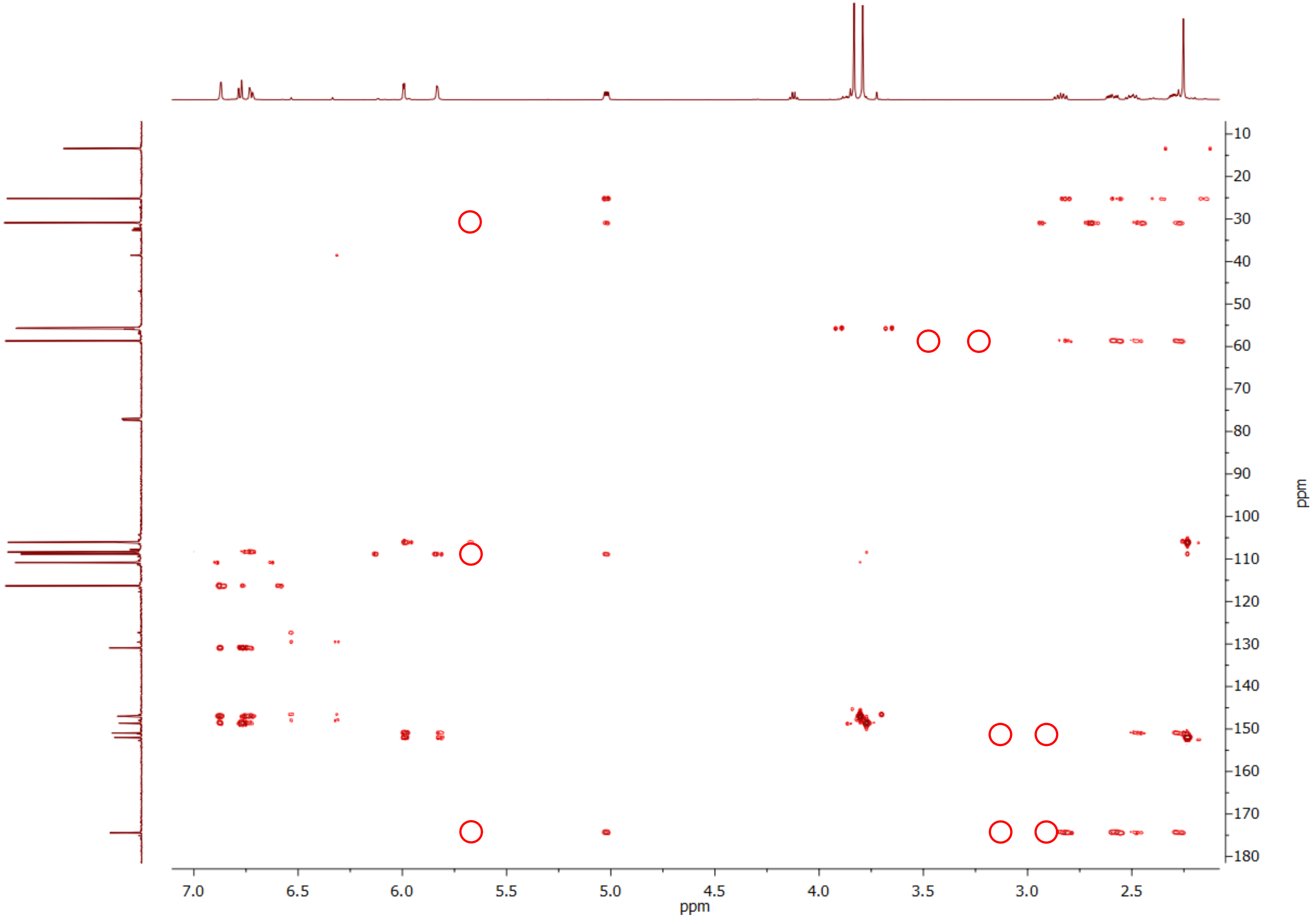

1-(2-Bromo-4-methylphenyl)-5-(thiophen-2-yl)pyrrolidin-2-one (2g)

^{1}H NMR ($CDCl_3$, 600 MHz)

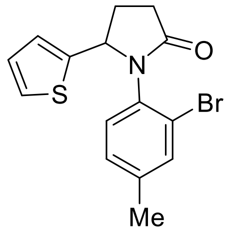

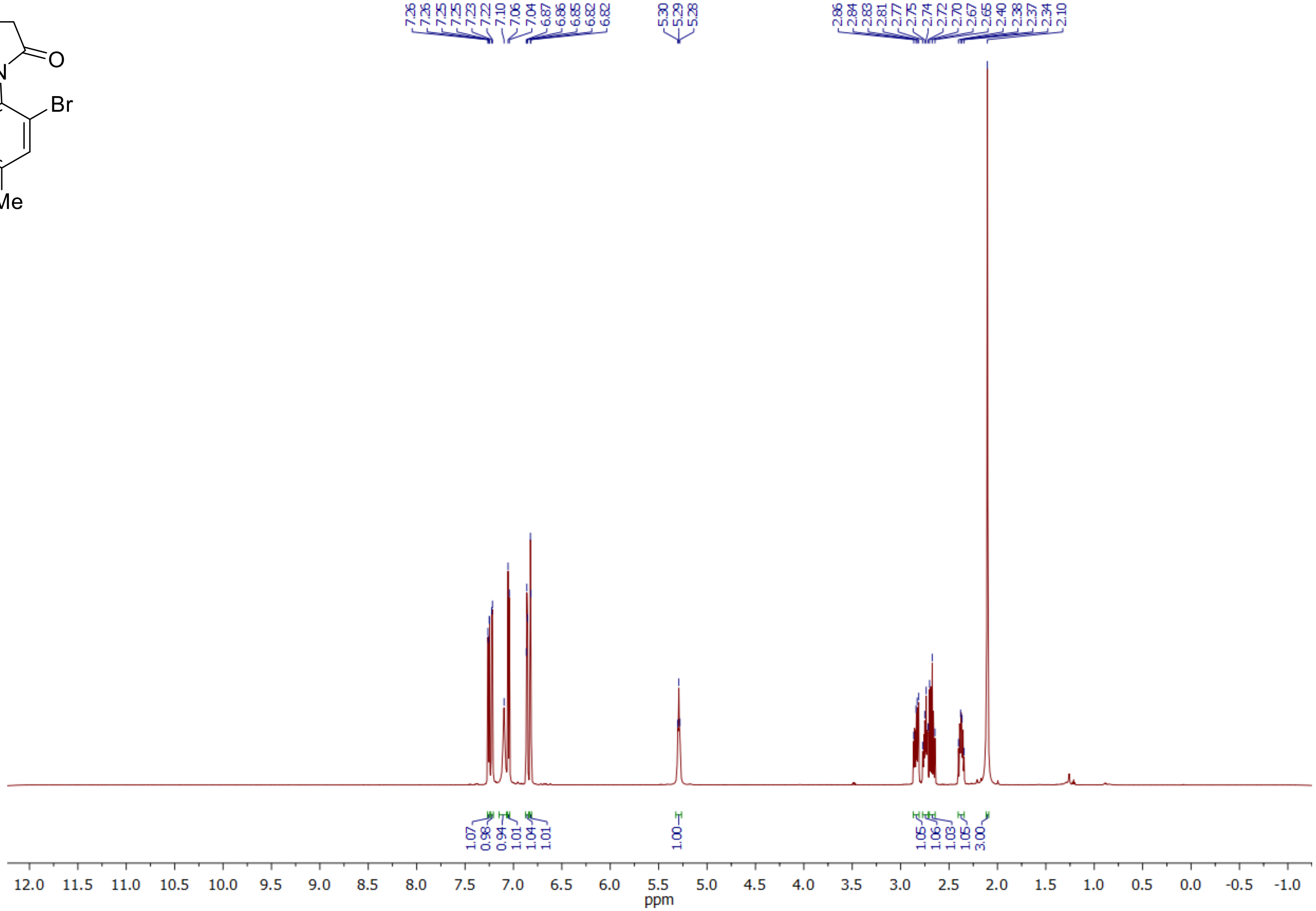

1-(2-Bromo-4-methylphenyl)-5-(thiophen-2-yl)pyrrolidin-2-one (2g)

^{13}C NMR ($CDCl_3$, 150 MHz)

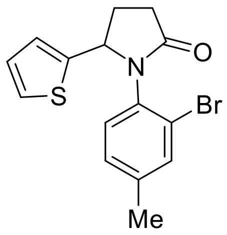

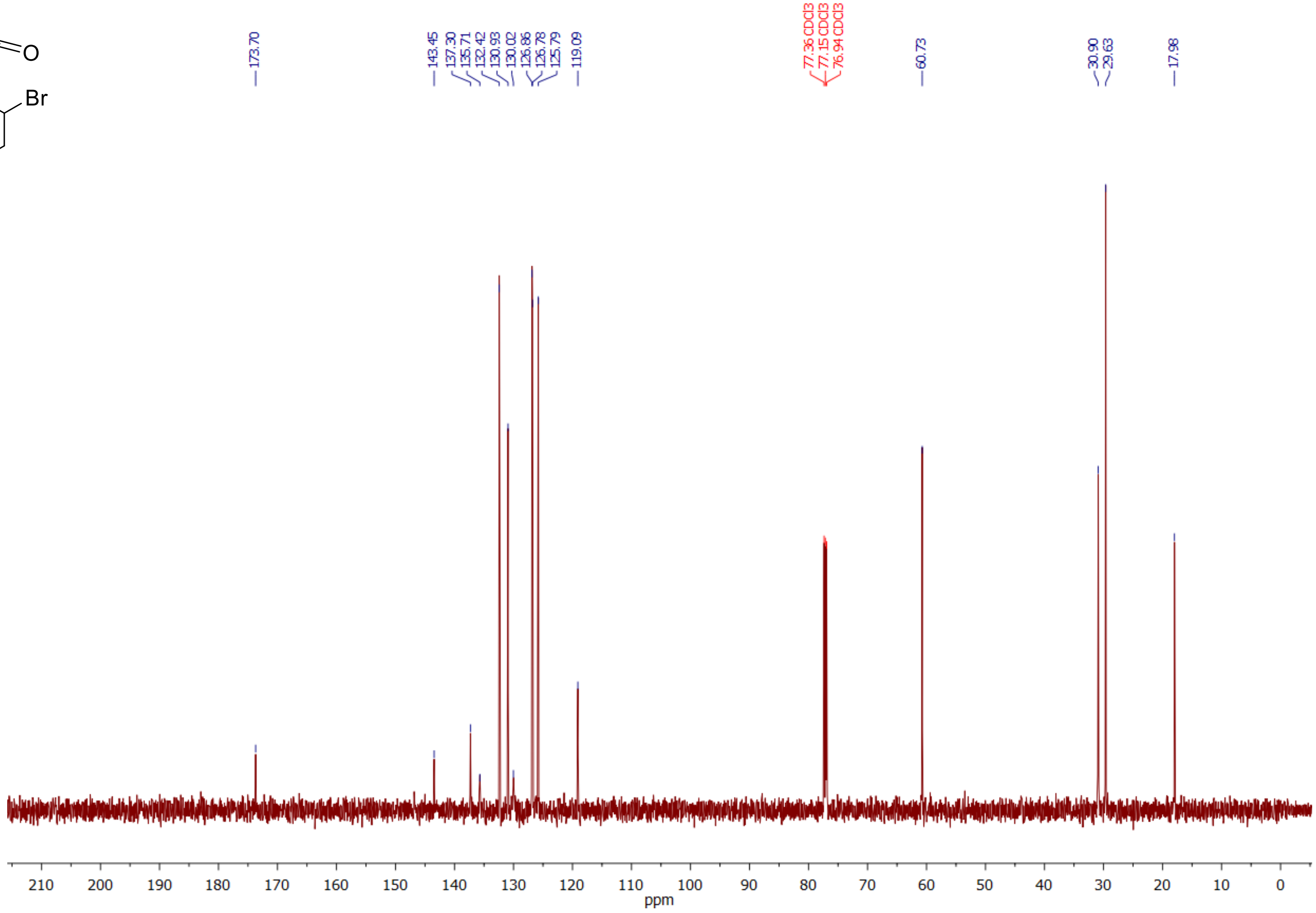

1-(2-Bromo-4-methylphenyl)-5-(thiophen-2-yl)pyrrolidin-2-one (2g)

HSQC ^{1}H-^{13}C ($CDCl_3$)

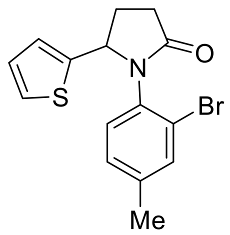

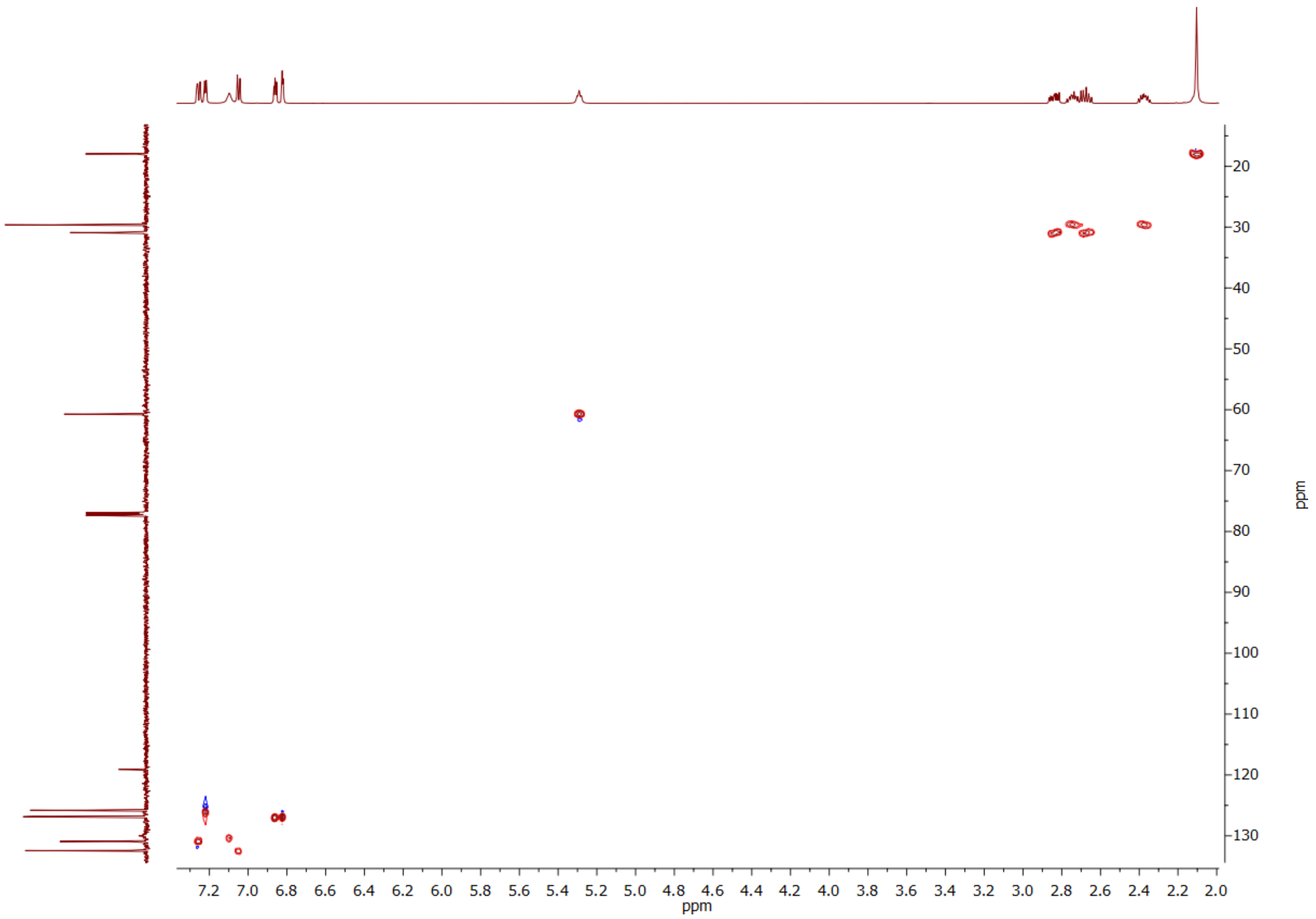

(*E*)-1-Phenyl-5-styrylpyrrolidin-2-one (2h)

^{1}H NMR ($CDCl_3$, 600 MHz)

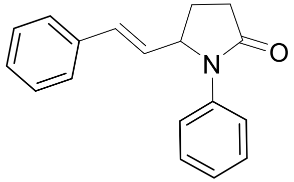

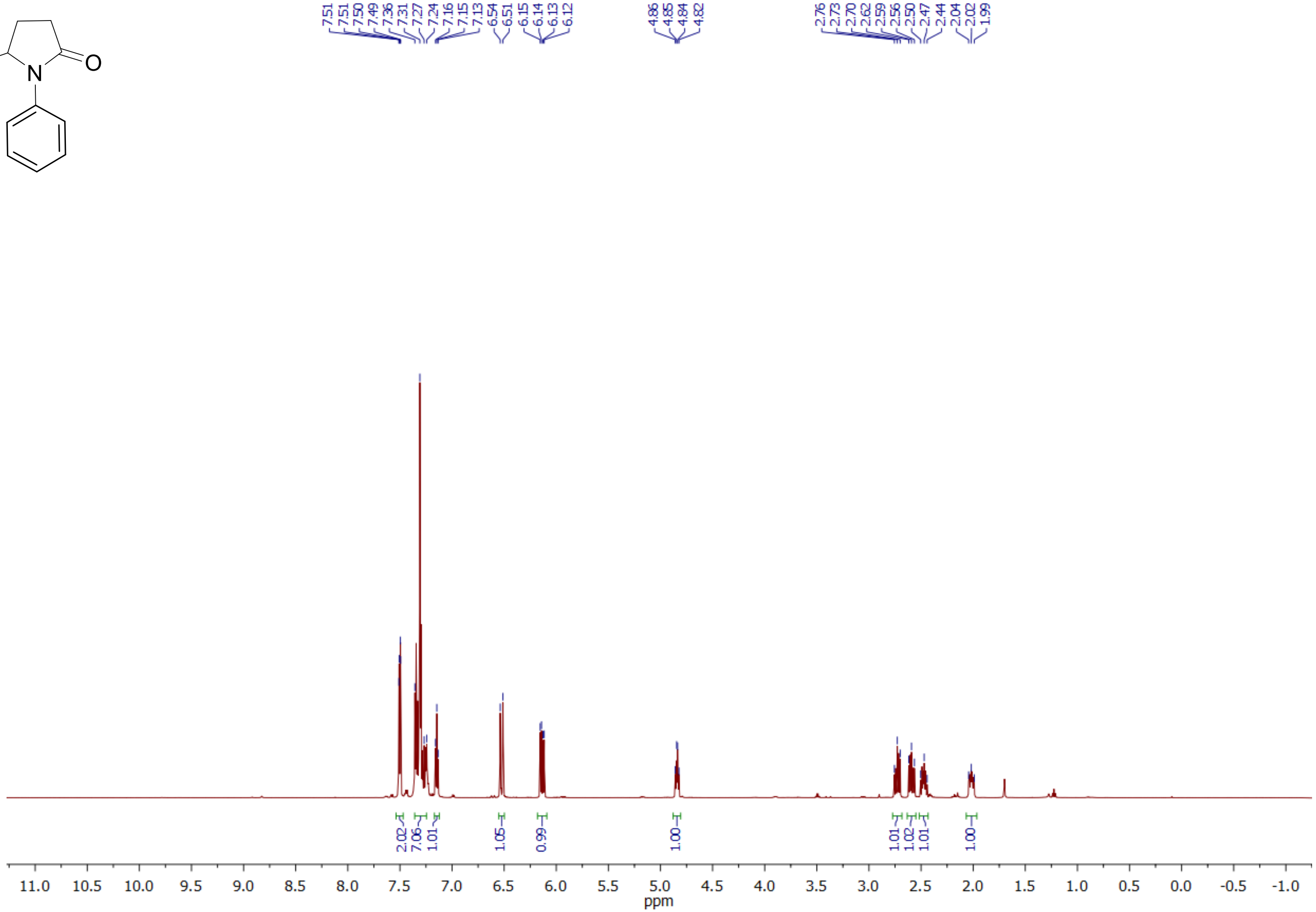

(*E*)-1-Phenyl-5-styrylpyrrolidin-2-one (2h)

^{13}C NMR ($CDCl_3$, 150 MHz)

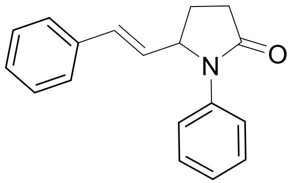

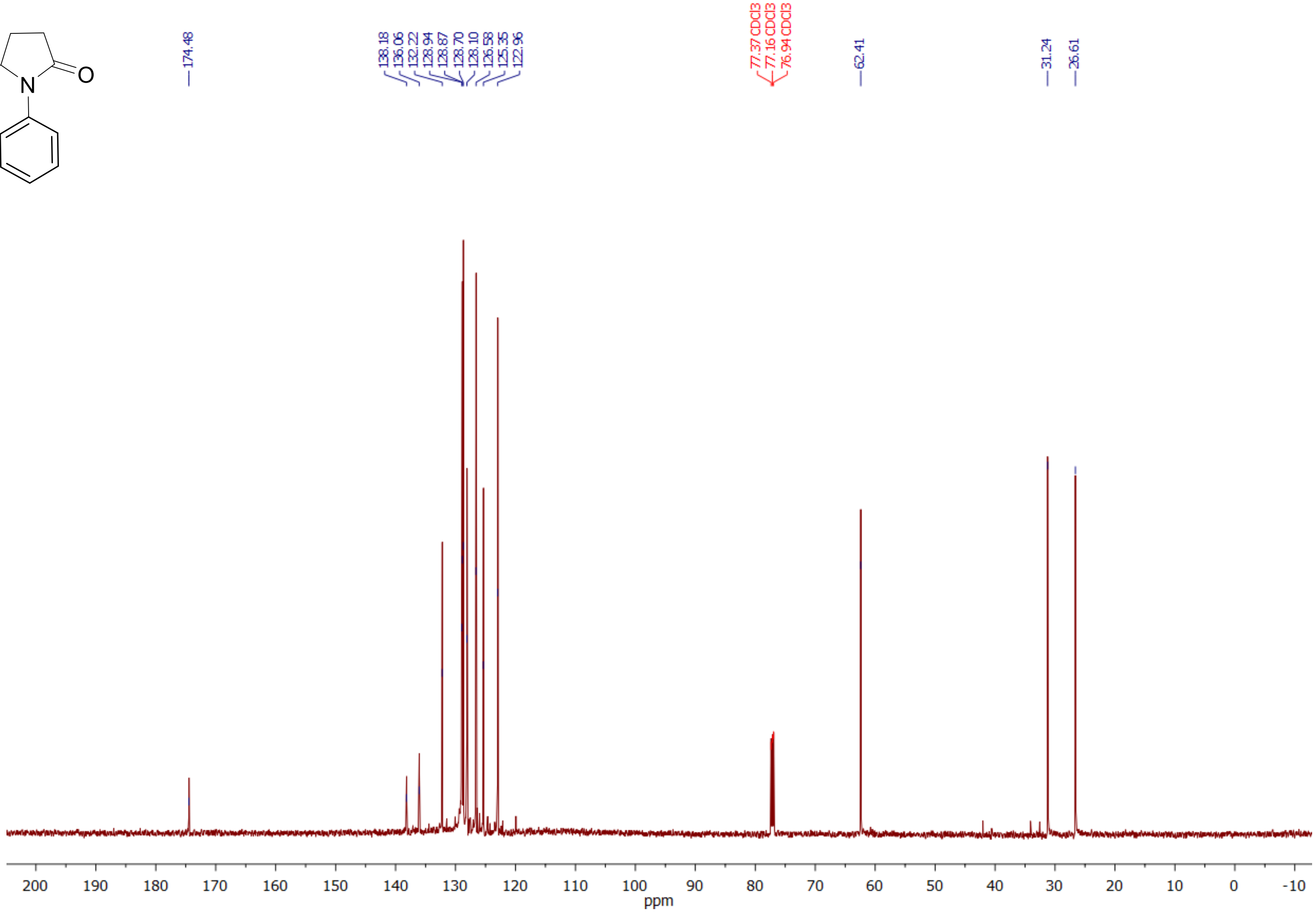

5-(2-Nitrophenyl)-1-phenylpyrrolidin-2-one (2i)

^{1}H NMR ($CDCl_3$, 600 MHz)

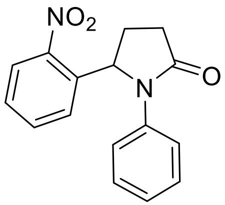

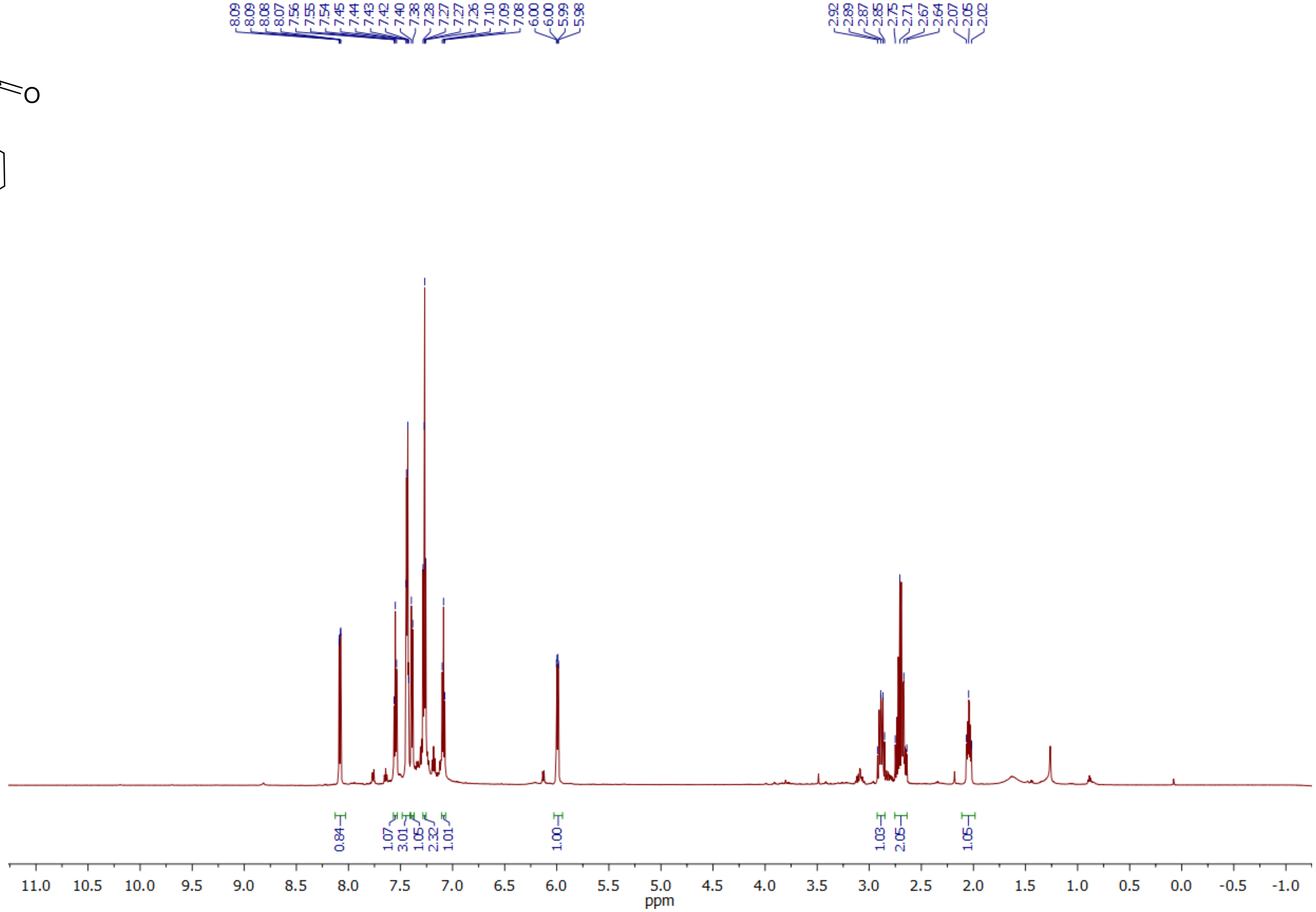

5-(2-Nitrophenyl)-1-phenylpyrrolidin-2-one (2i)

^{13}C NMR ($CDCl_3$, 150 MHz)

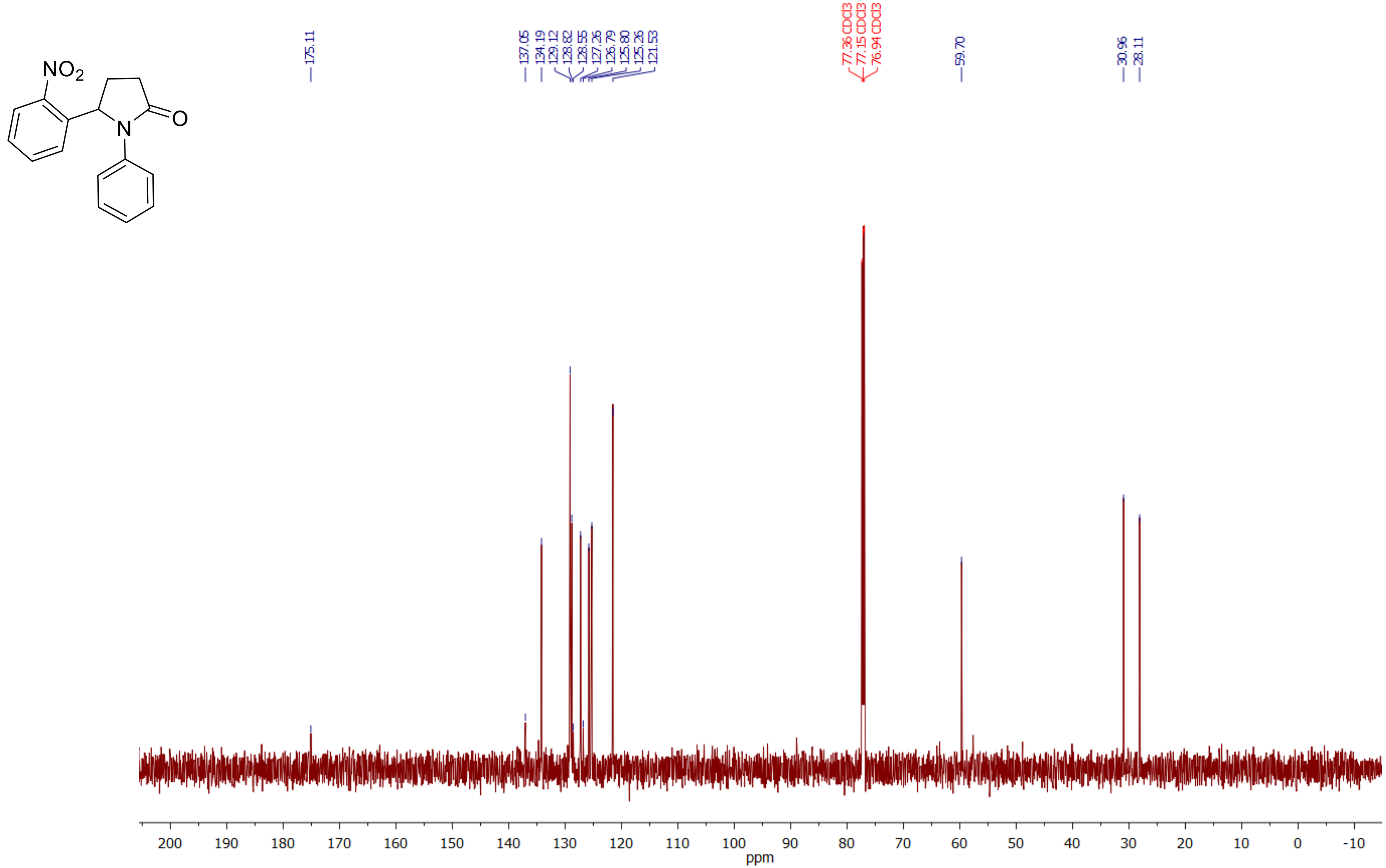

1-Benzyl-5-(3,4,5-trimethoxyphenyl)pyrrolidin-2-one (3a)

^{1}H NMR ($CDCl_3$, 600 MHz)

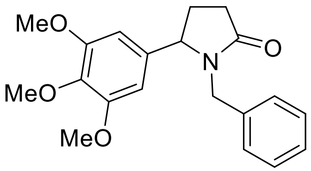

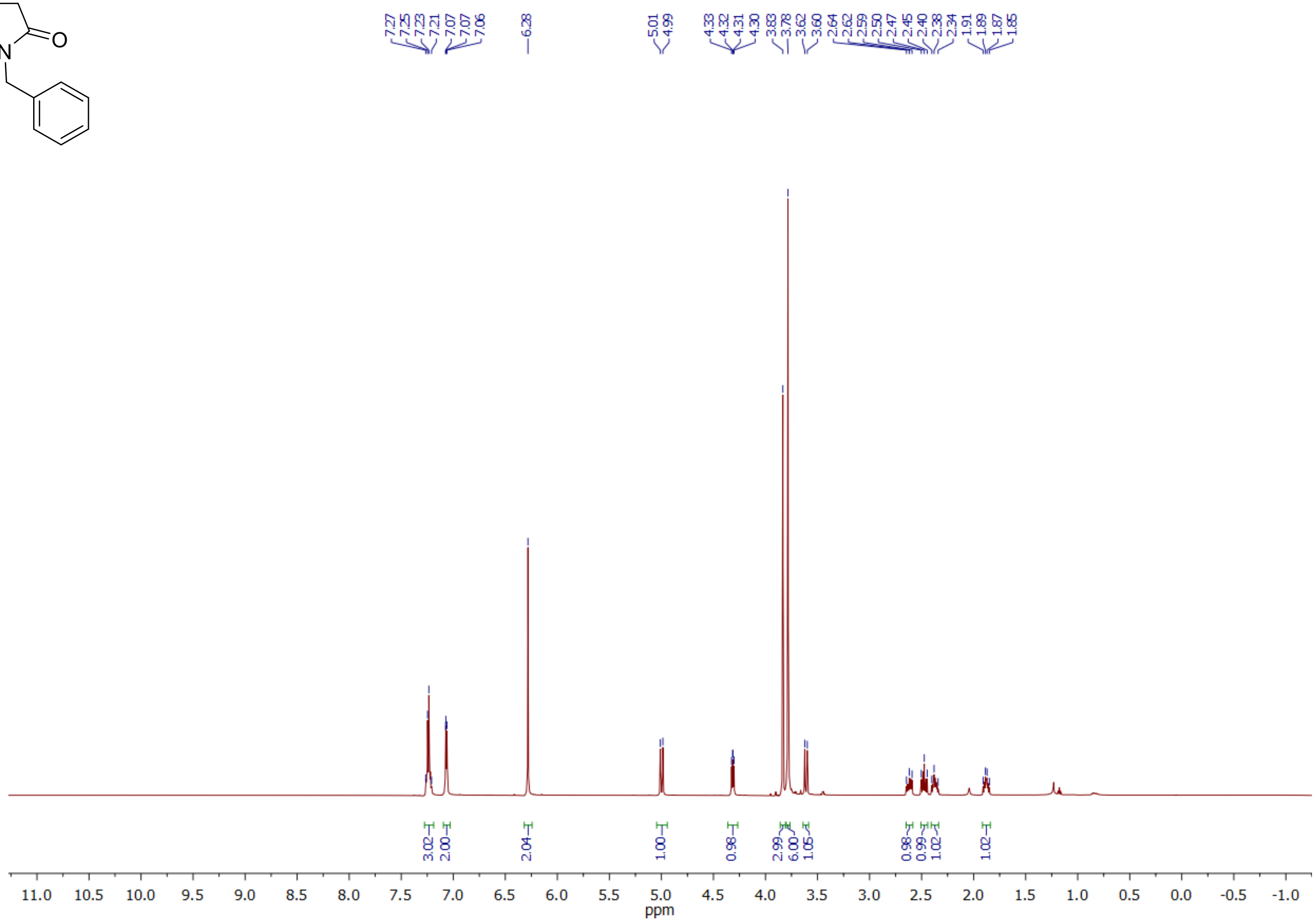

1-Benzyl-5-(3,4,5-trimethoxyphenyl)pyrrolidin-2-one (3a)

^{13}C NMR ($CDCl_3$, 150 MHz)

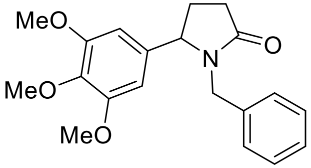

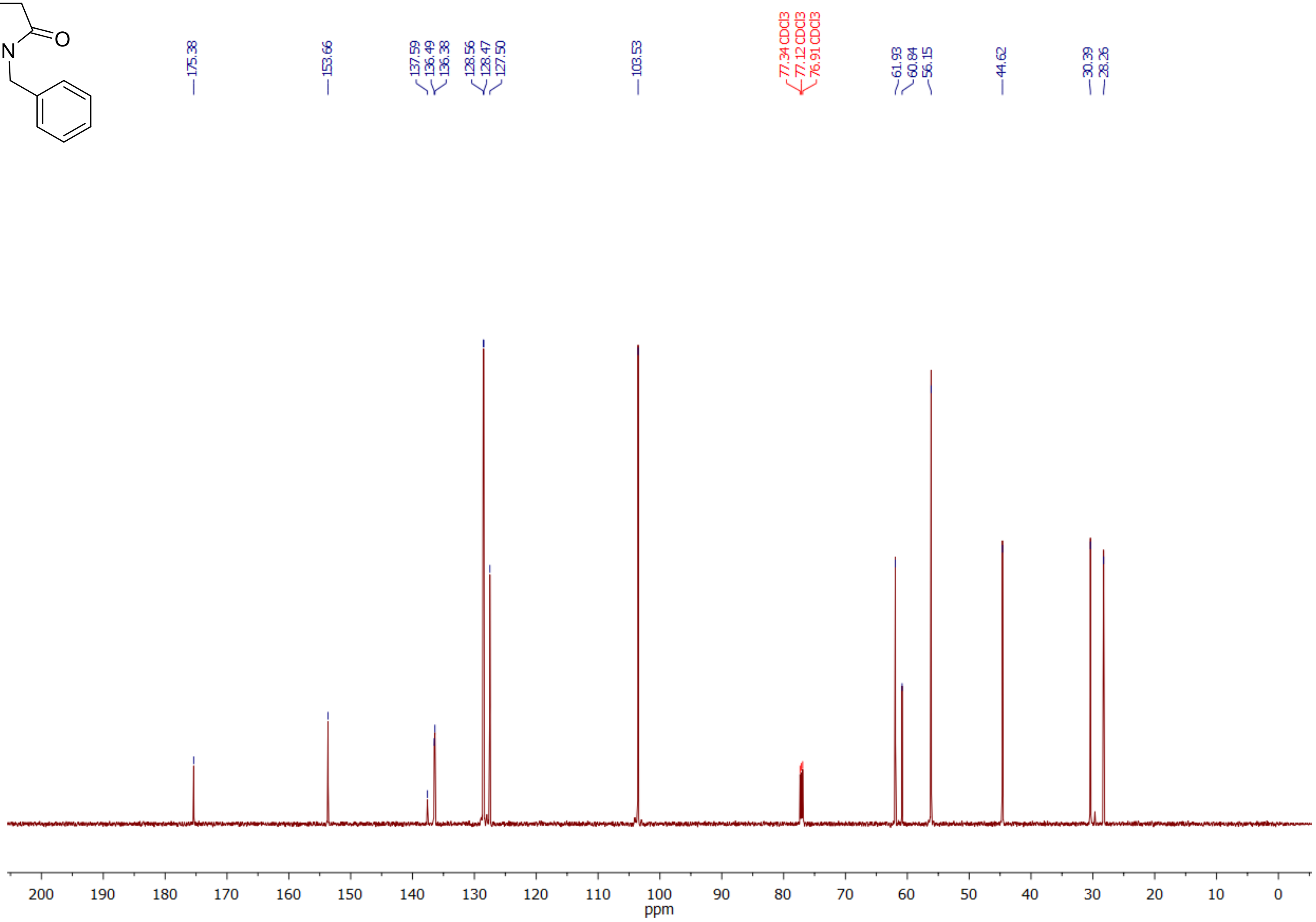

1-Benzyl-5-(3,4,5-trimethoxyphenyl)pyrrolidin-2-one (3a)

HSQC ^{1}H-^{13}C ($CDCl_3$)

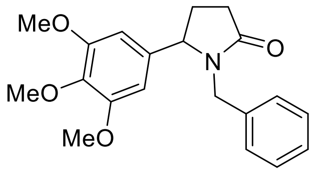

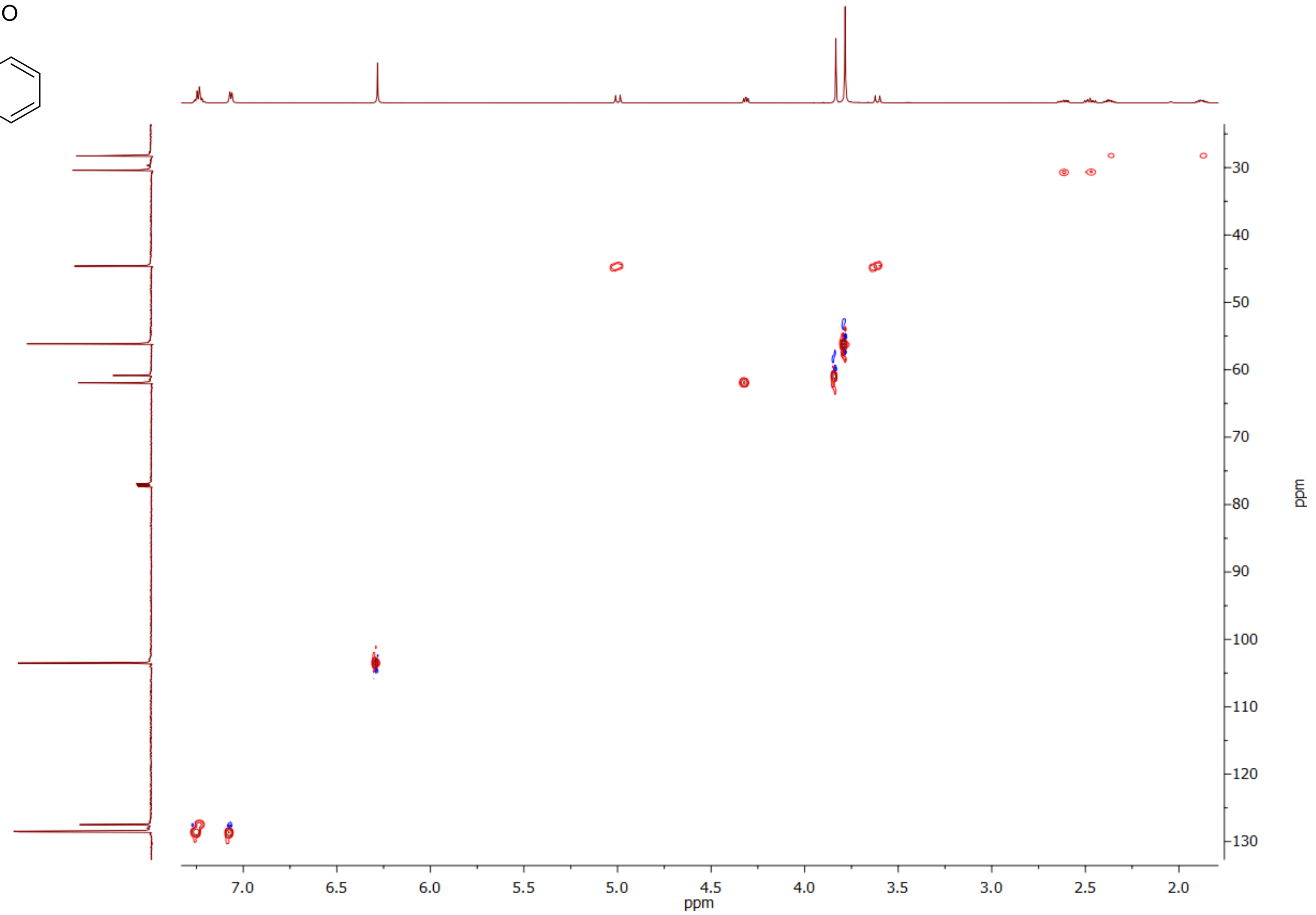

1-(2,5-Dimethoxybenzyl)-5-(3,4-dimethoxyphenyl)pyrrolidin-2-on (3d)

^{1}H NMR ($CDCl_3$, 500 MHz)

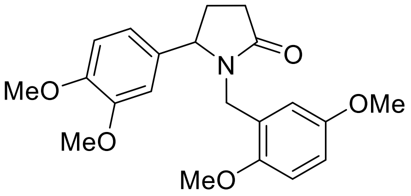

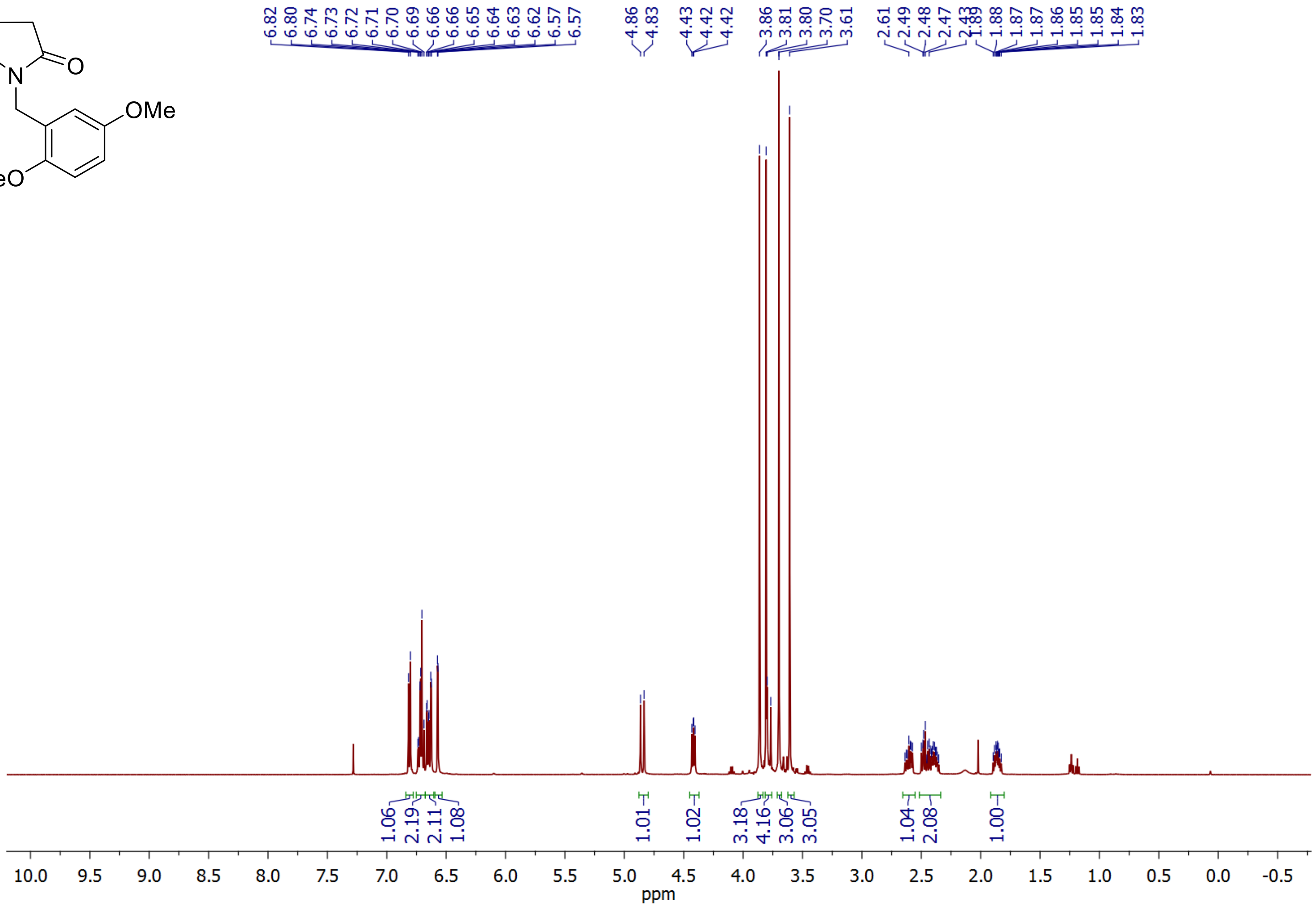

1-(2,5-Dimethoxybenzyl)-5-(3,4-dimethoxyphenyl)pyrrolidin-2-on (3d)

^{13}C NMR ($CDCl_3$, 125 MHz)

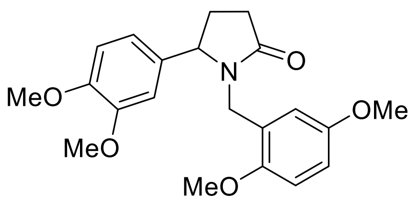

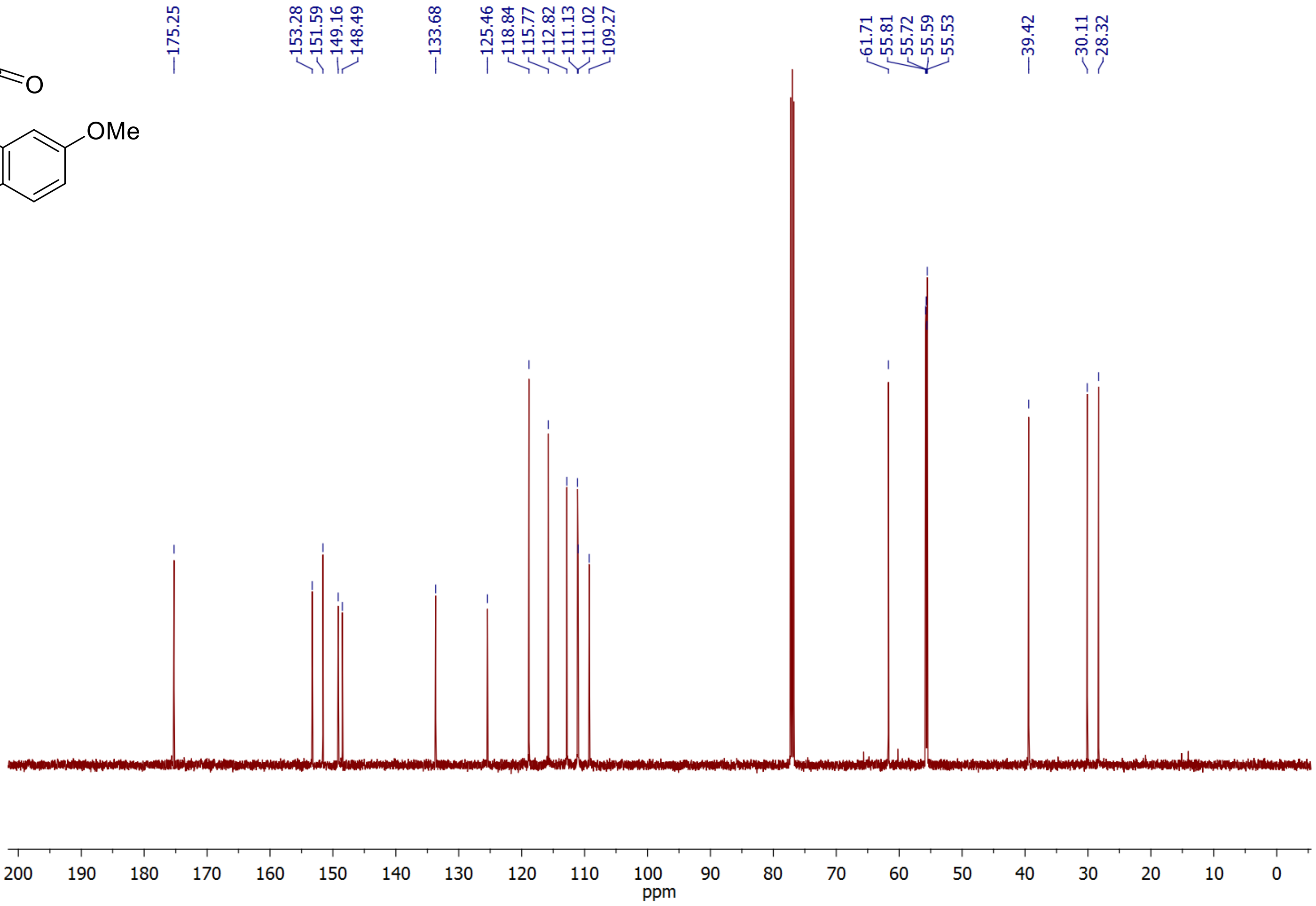

1-(2,5-Dimethoxybenzyl)-5-(3,4-dimethoxyphenyl)pyrrolidin-2-on (3d)

HSQC ^1H-^{13}C ($CDCl_3$)

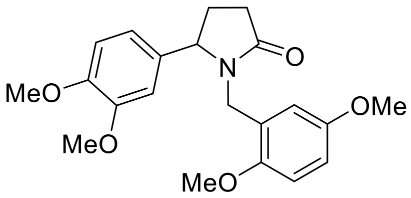

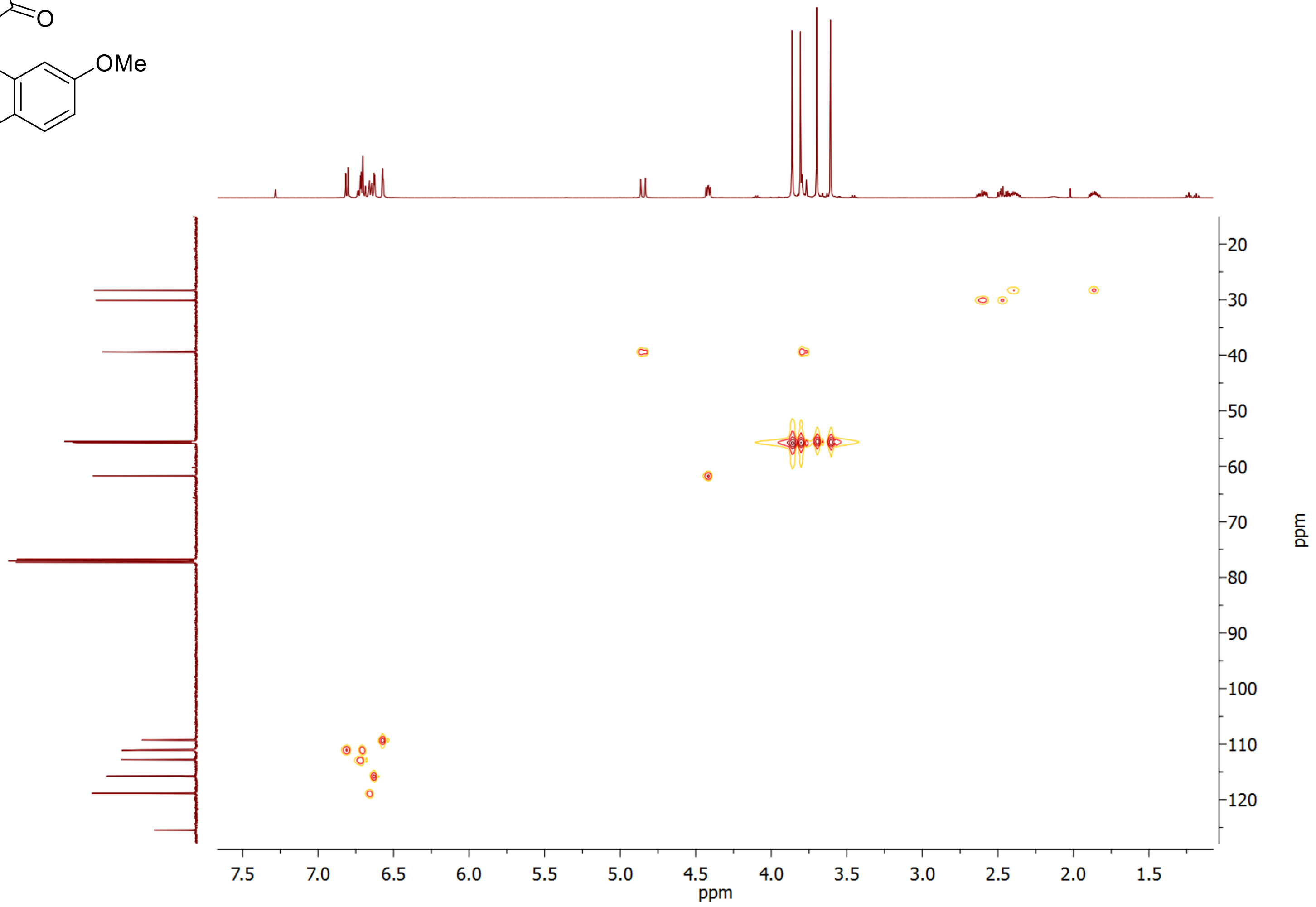

1-Furfuryl-5-(2,4,5-trimethoxyphenyl)pyrrolidin-2-one (**3e**)

^{1}H NMR ($CDCl_3$, 600 MHz)

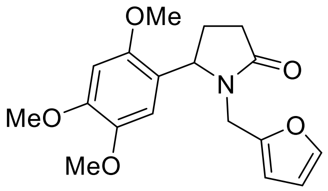

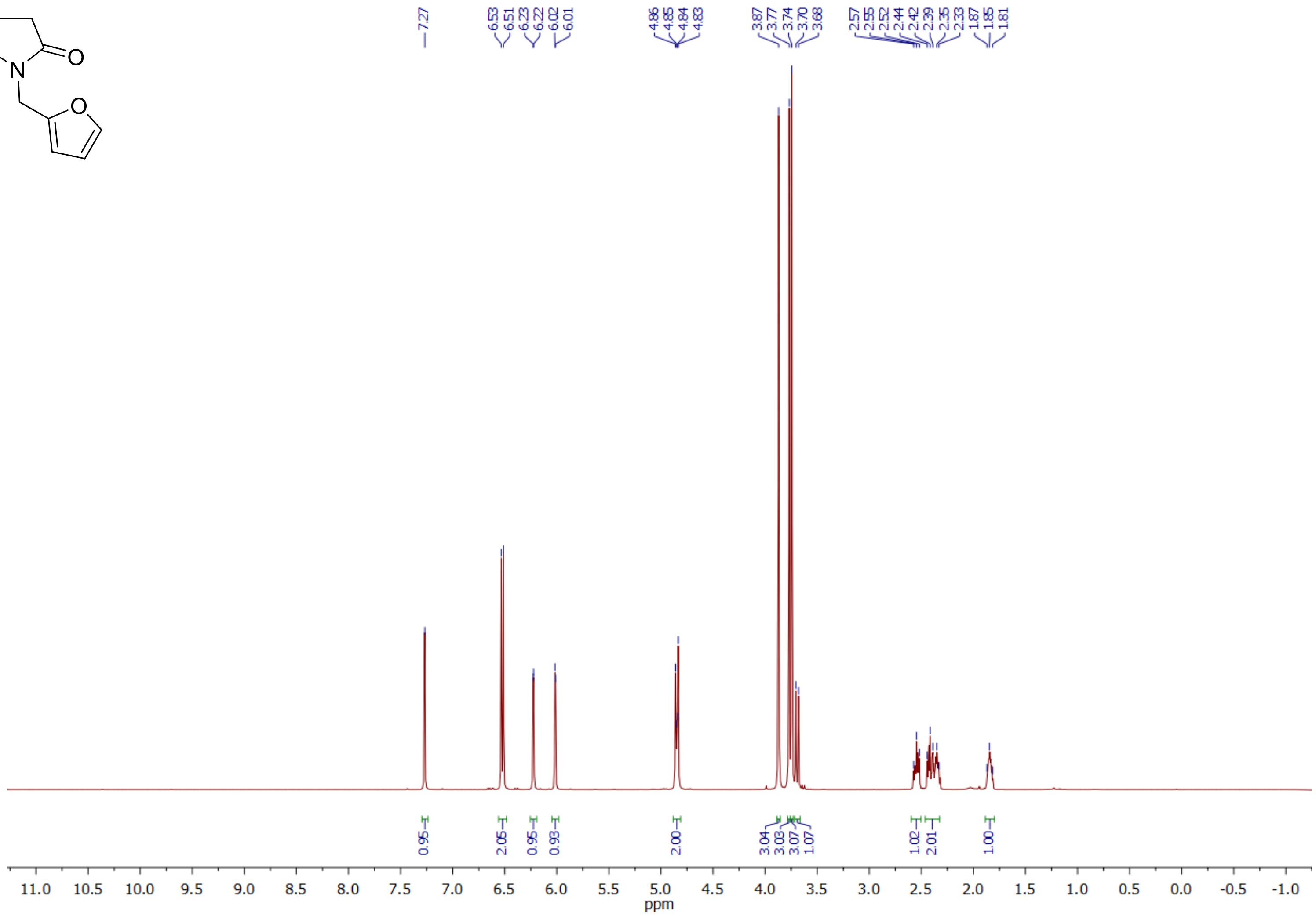

1-Furfuryl-5-(2,4,5-trimethoxyphenyl)pyrrolidin-2-one (**3e**)

^{13}C NMR ($CDCl_3$, 150 MHz)

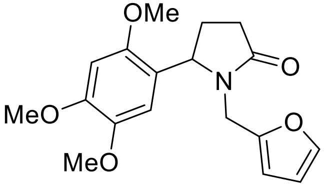

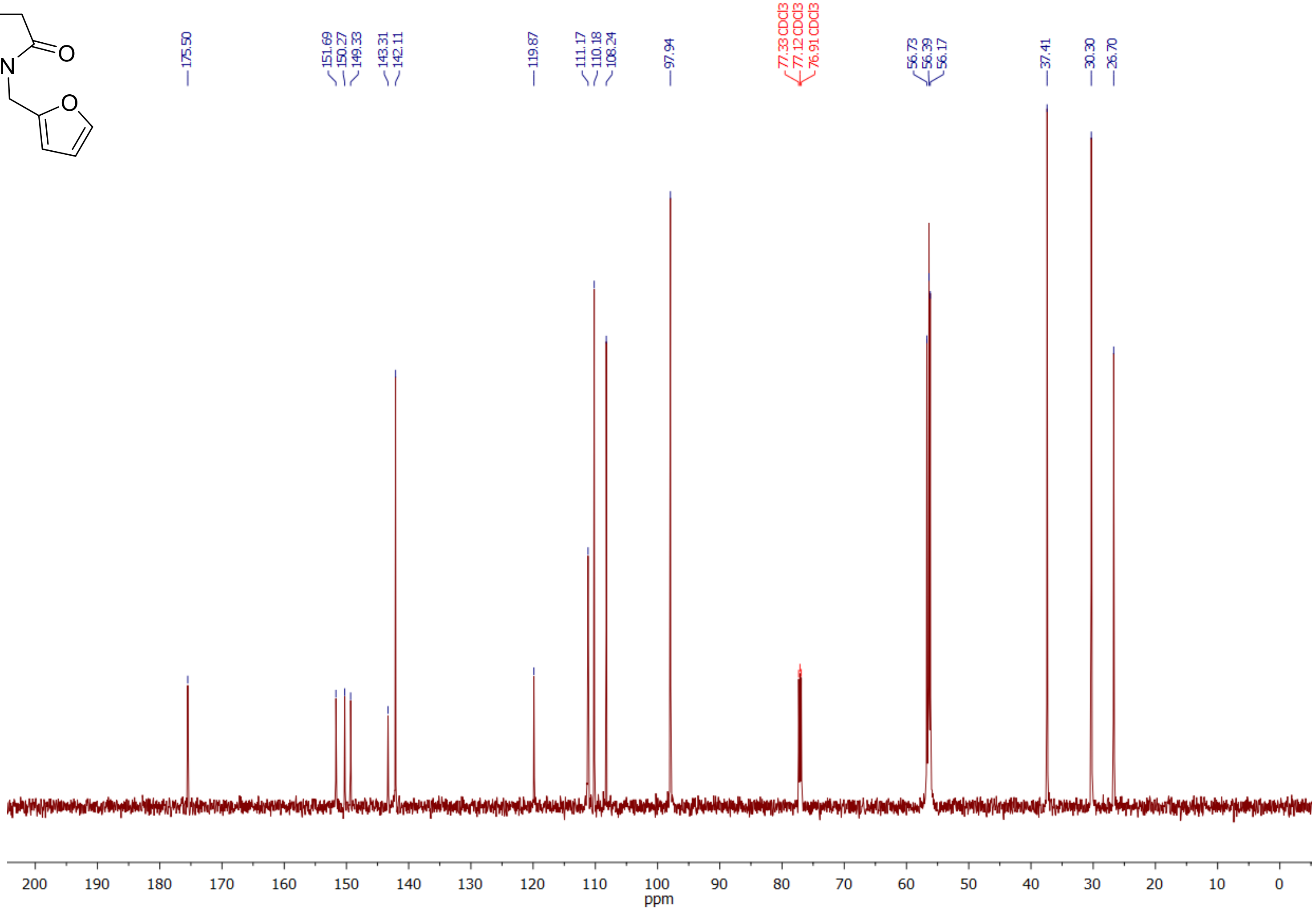

1-Furfuryl-5-(2,4,5-trimethoxyphenyl)pyrrolidin-2-one (3e)

HSQC ^{1}H-^{13}C ($CDCl_3$)

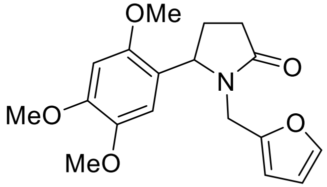

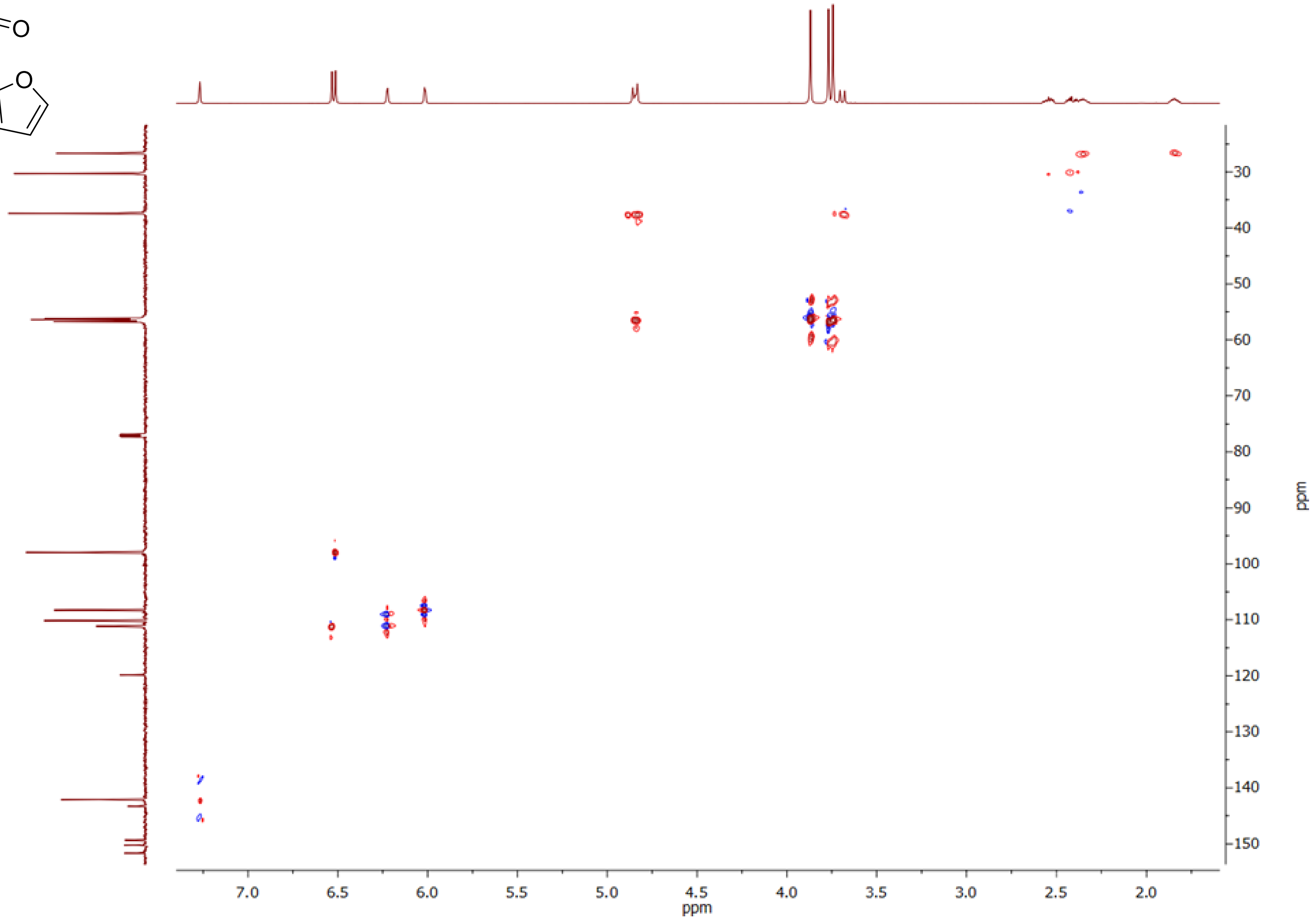

1-Furfuryl-5-(2,4,5-trimethoxyphenyl)pyrrolidin-2-one (3e)

HMBC ^{1}H-^{13}C ($CDCl_3$)

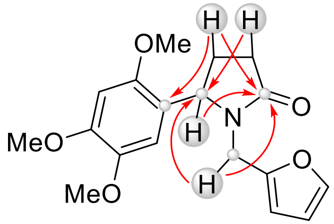

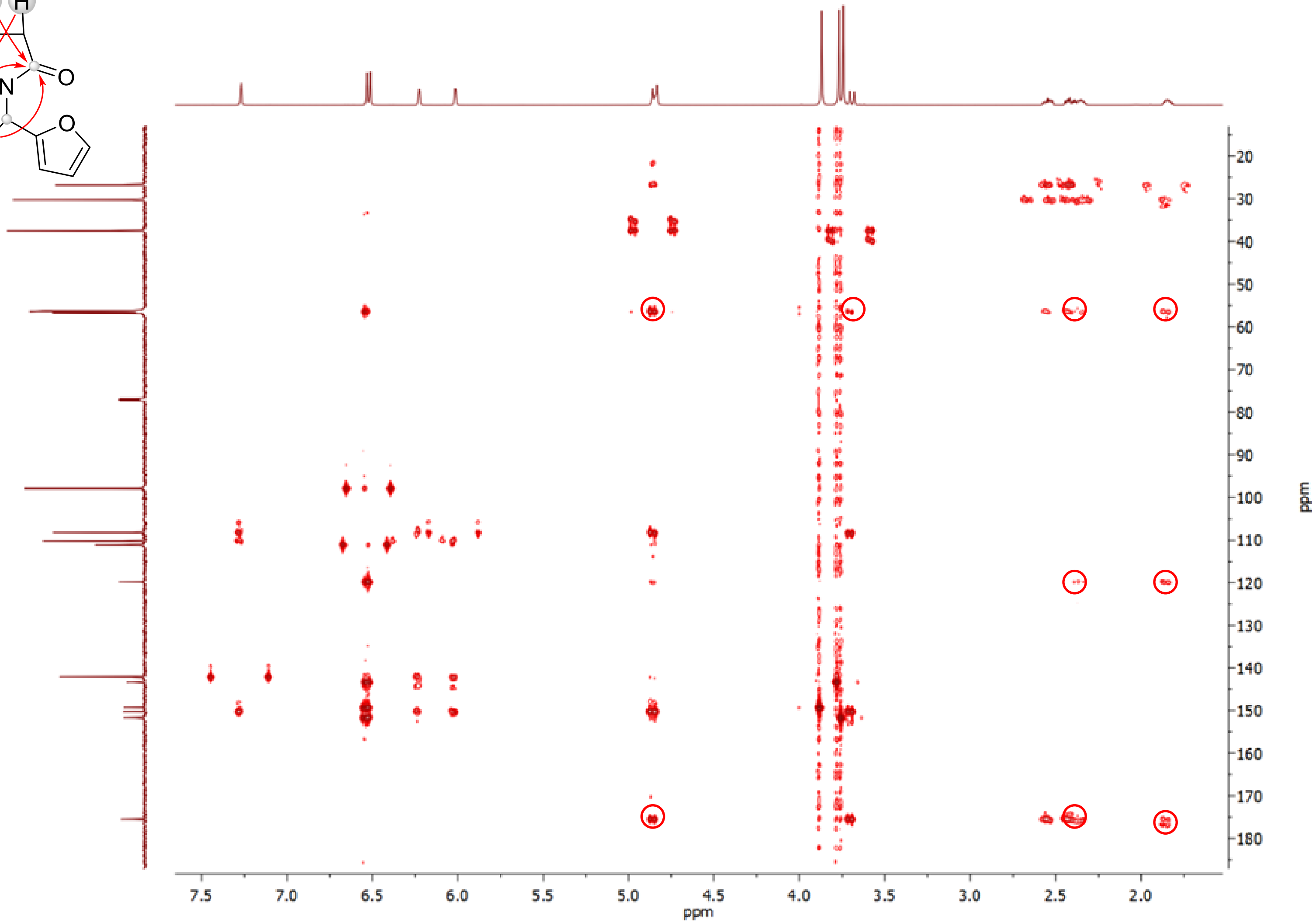

1-[(1*H*-Indol-4-yl)methyl]-5-(3,4-dimethoxyphenyl)pyrrolidin-2-one (3f)

^{1}H NMR ($CDCl_3$, 500 MHz)

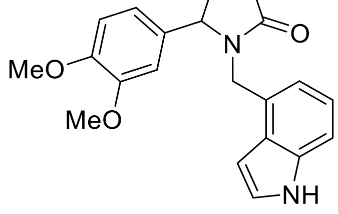

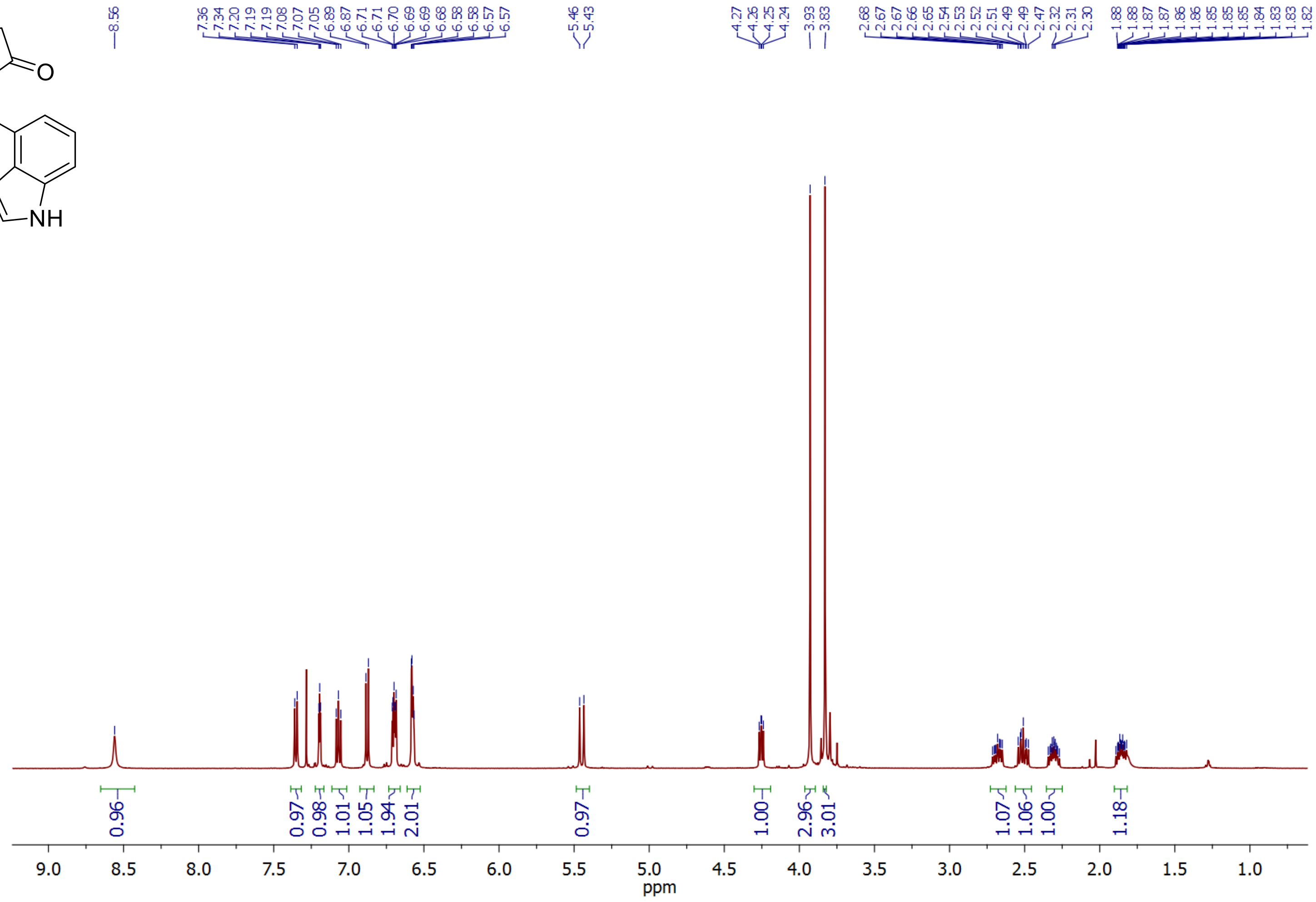

1-[(1*H*-Indol-4-yl)methyl]-5-(3,4-dimethoxyphenyl)pyrrolidin-2-one (3f)

^{13}C NMR ($CDCl_3$, 125 MHz)

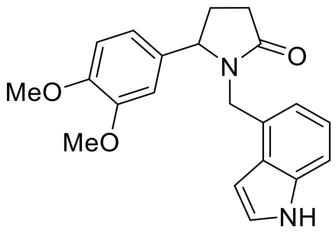

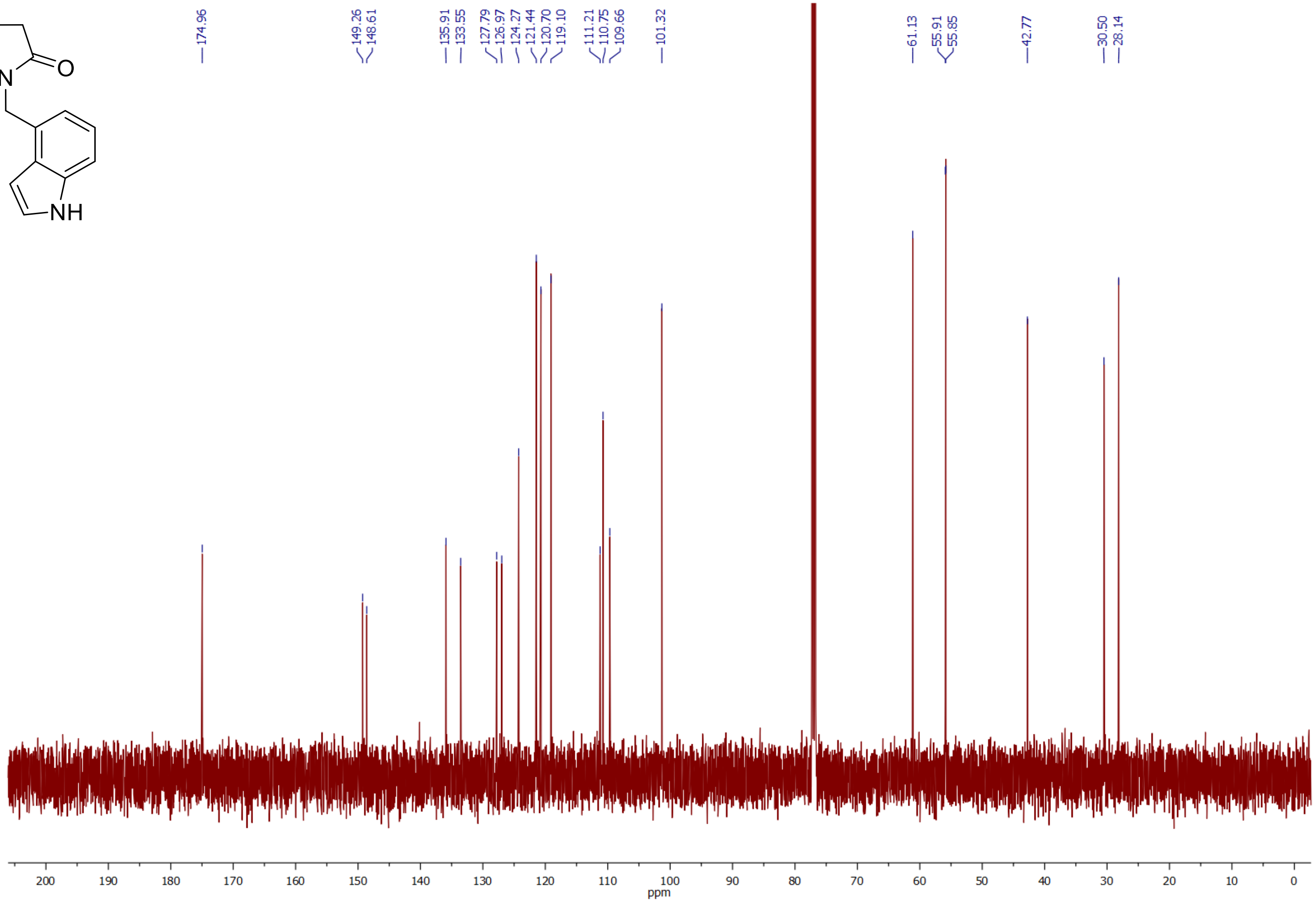

(*E*)-1-[2-(1*H*-Indol-3-yl)ethyl]-5-styrylpyrrolidin-2-one (**3g**)

^{1}H NMR ($CDCl_3$, 600 MHz)

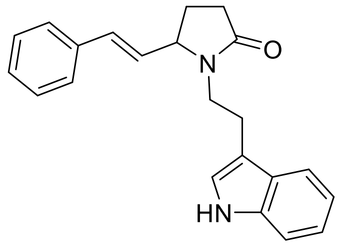

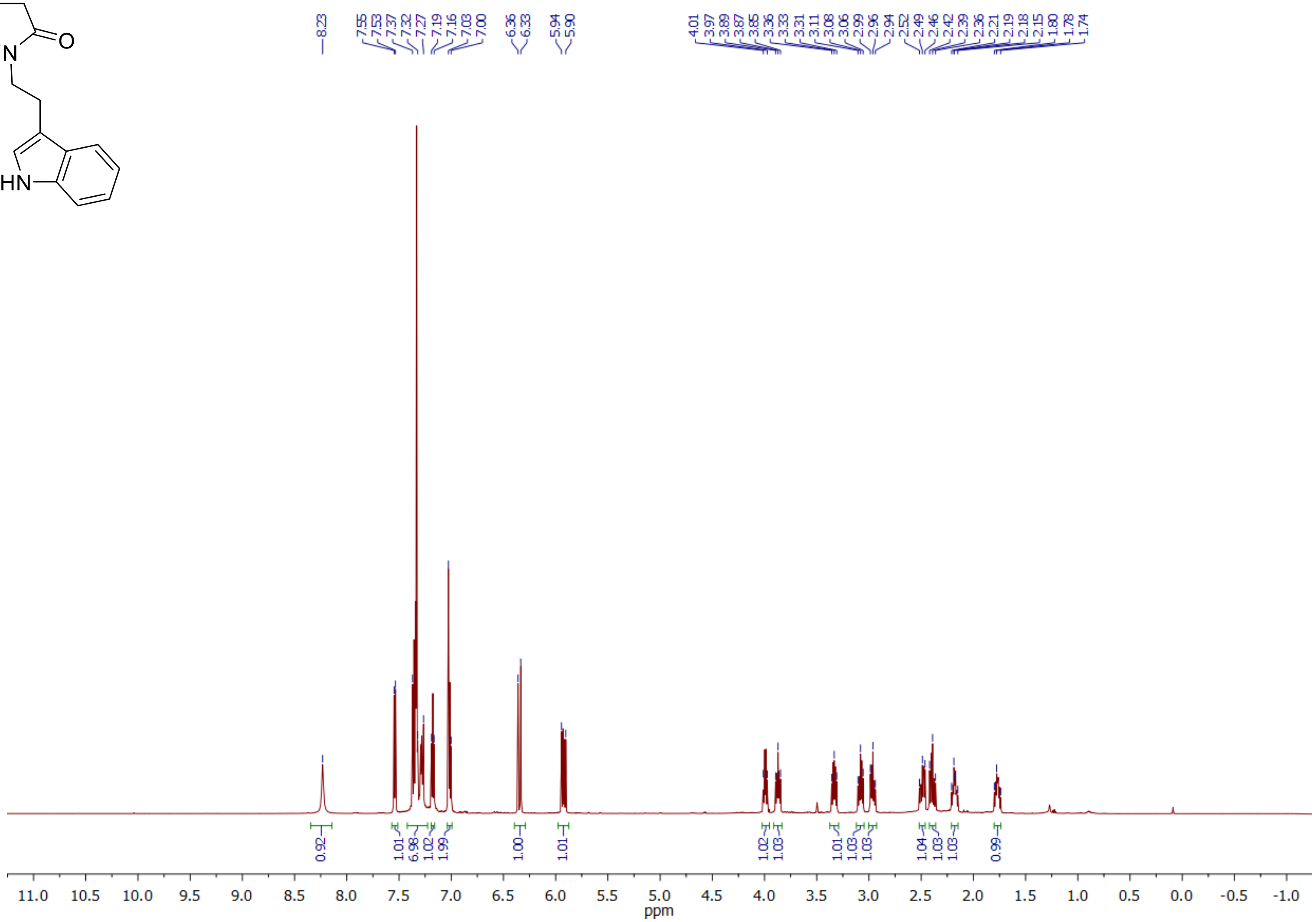

(*E*)-1-[2-(1*H*-Indol-3-yl)ethyl]-5-styrylpyrrolidin-2-one (**3g**)

^{13}C NMR ($CDCl_3$, 150 MHz)

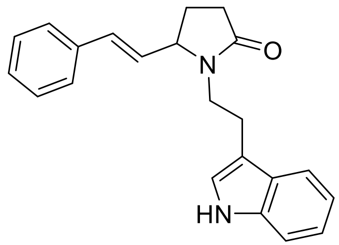

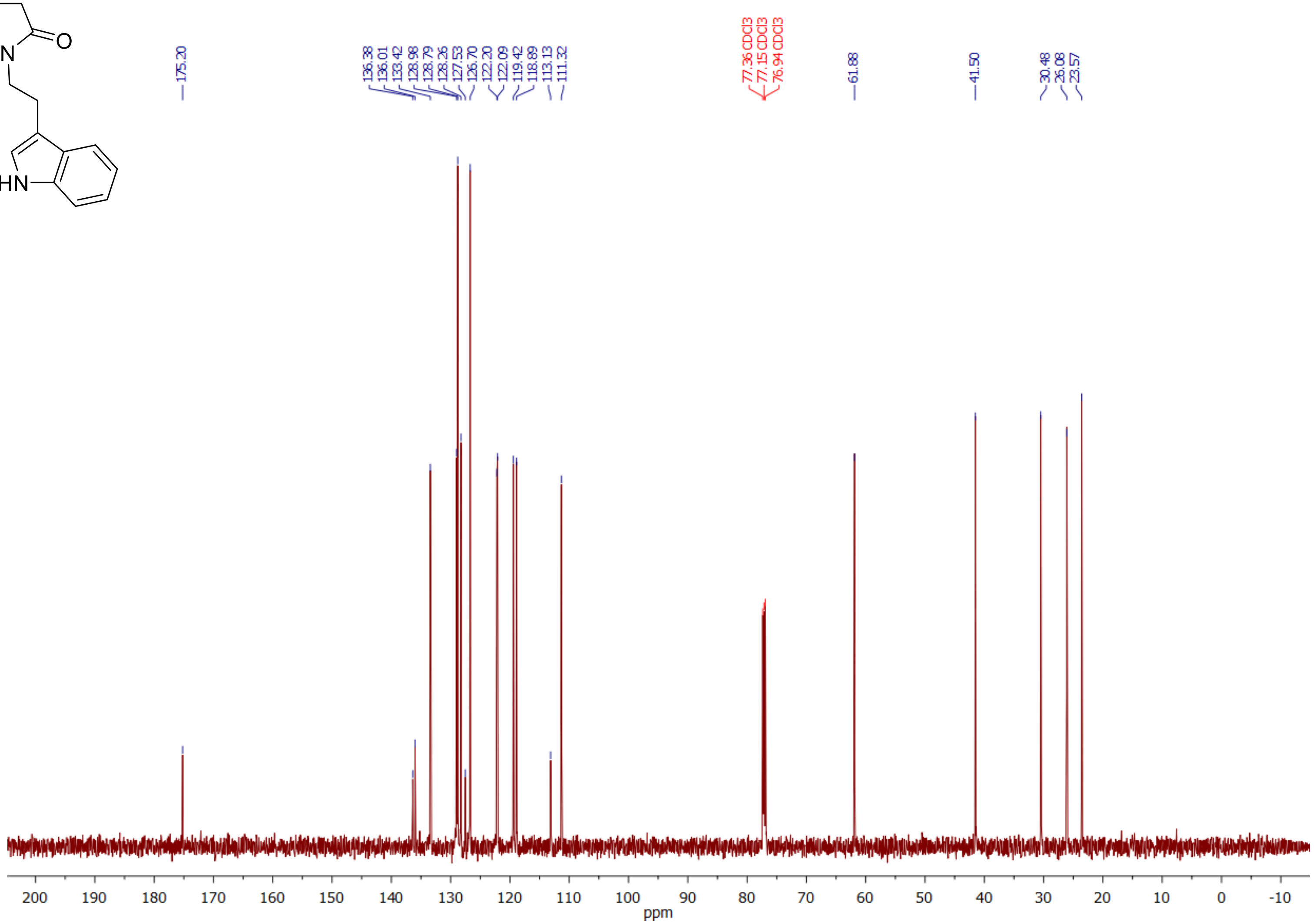

(*E*)-1-[2-(1*H*-Indol-3-yl)ethyl]-5-styrylpyrrolidin-2-one (**3g**)

HSQC ^{1}H-^{13}C ($CDCl_3$)

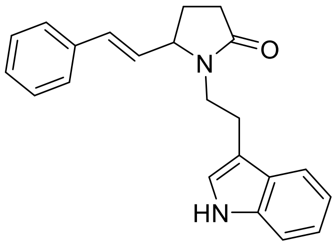

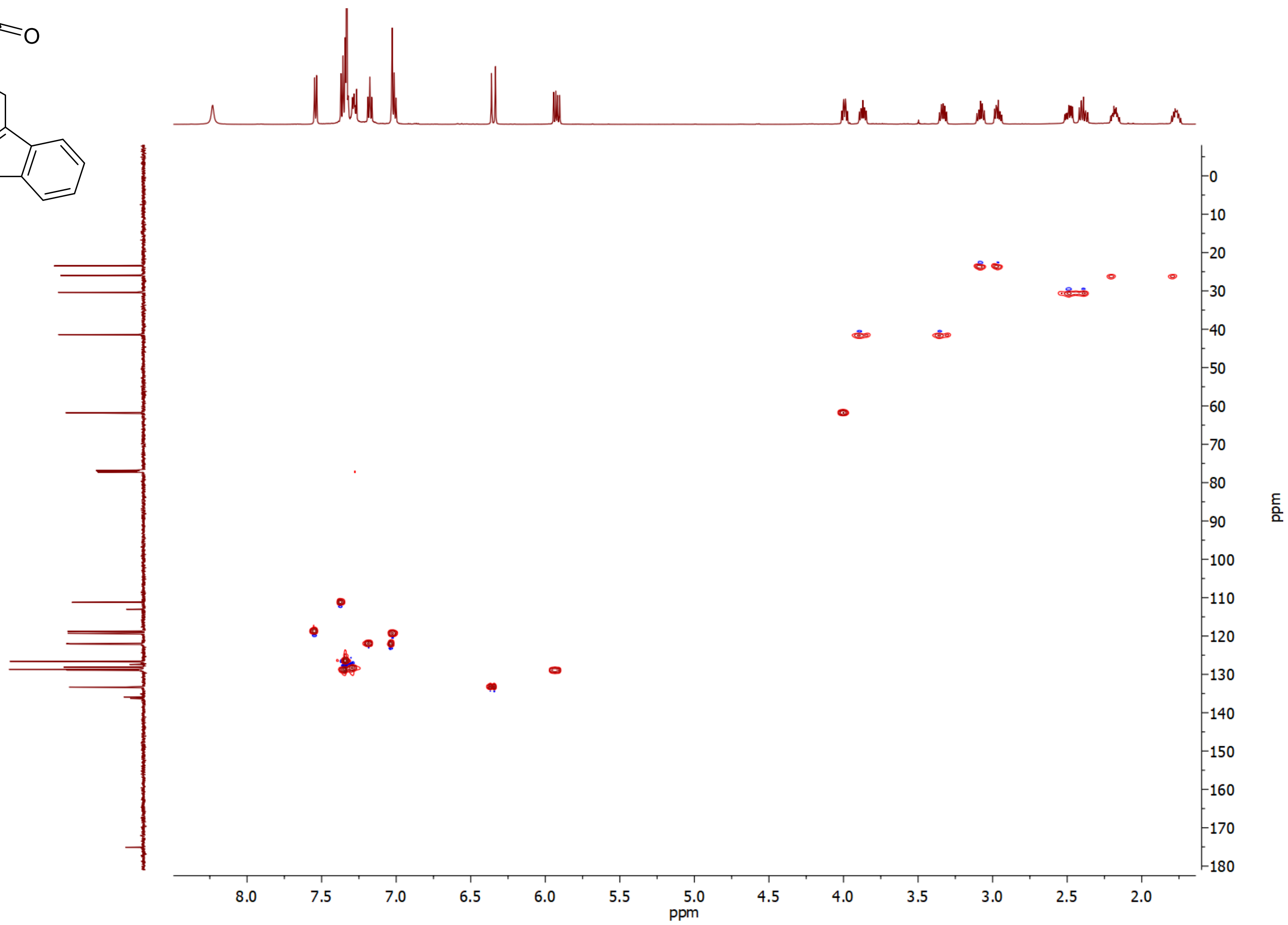

(*E*)-1-[2-(1*H*-Indol-3-yl)ethyl]-5-styrylpyrrolidin-2-one (3g)

HMBC ^{1}H-^{13}C ($CDCl_3$)

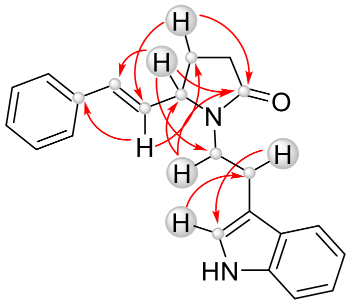

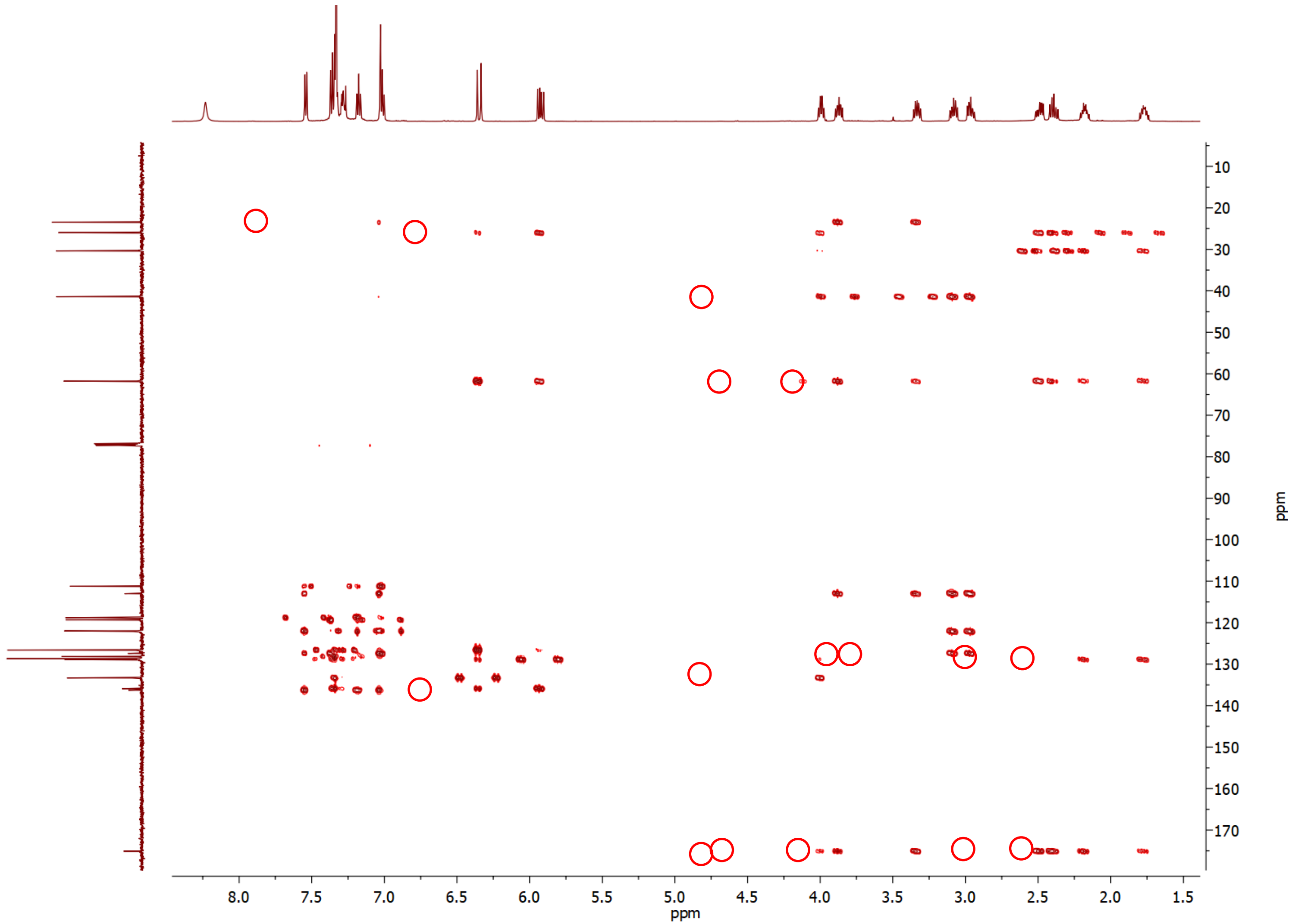

1-Cyclobutyl-5-(4-morpholinophenyl)pyrrolidin-2-one (**3h**)

^{1}H NMR ($CDCl_3$, 500 MHz)

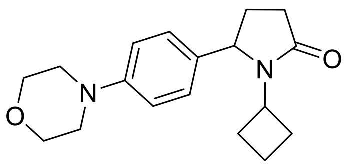

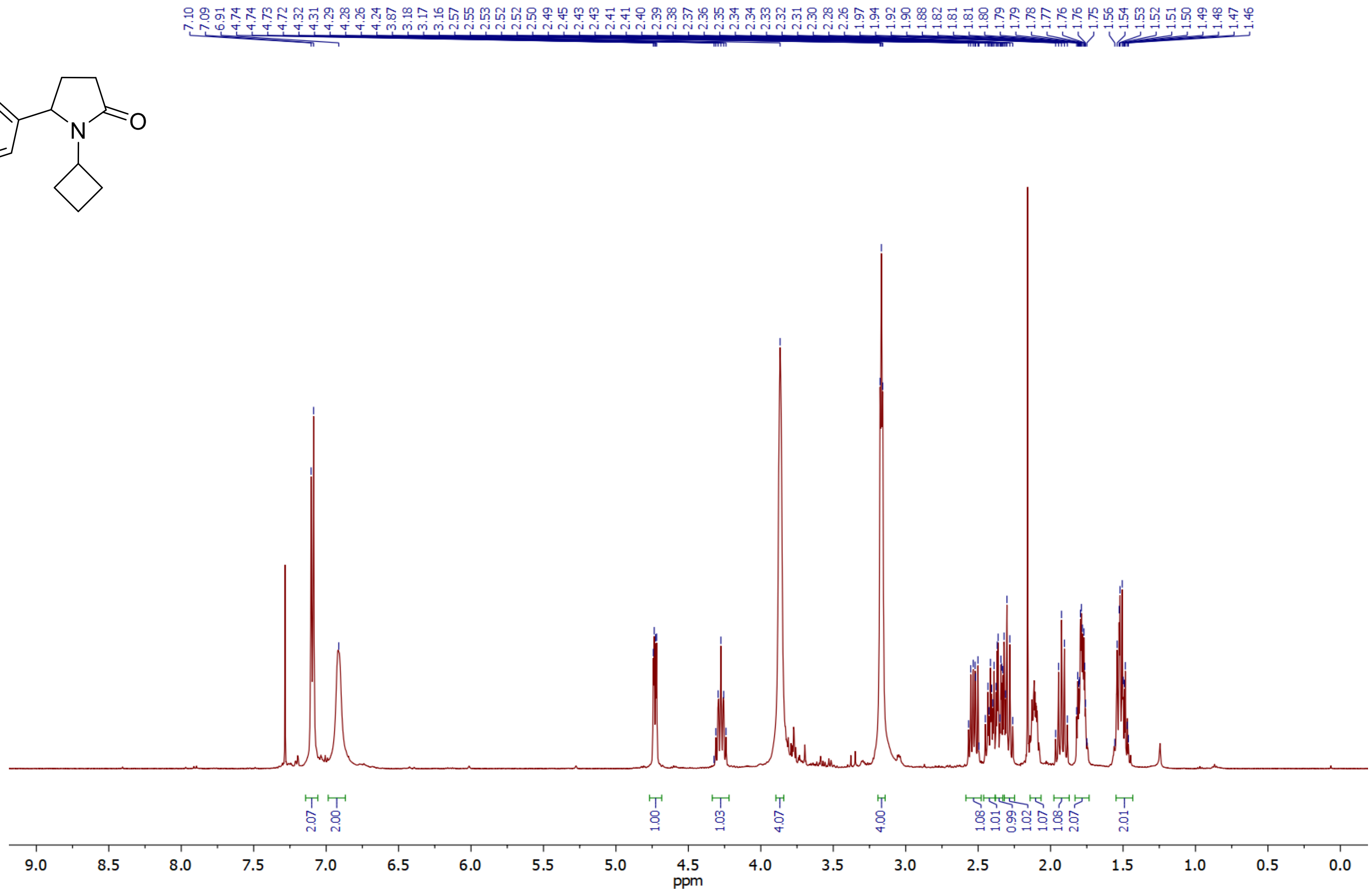

1-Cyclobutyl-5-(4-morpholinophenyl)pyrrolidin-2-one (**3h**)

^{13}C NMR ($CDCl_3$, 125 MHz)

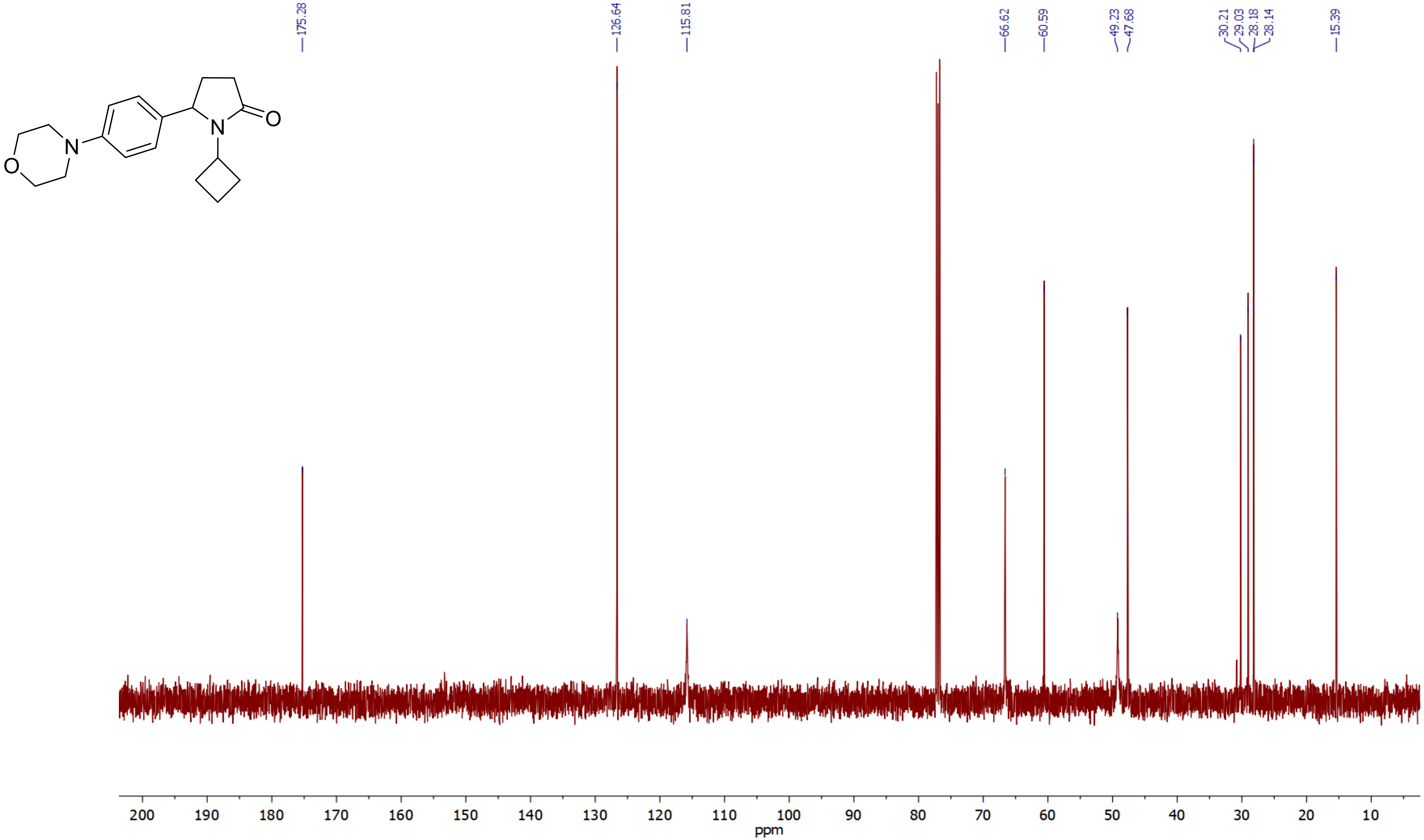

1-Cyclobutyl-5-(4-morpholinophenyl)pyrrolidin-2-one (**3h**)

HSQC ^{1}H-^{13}C ($CDCl_3$)

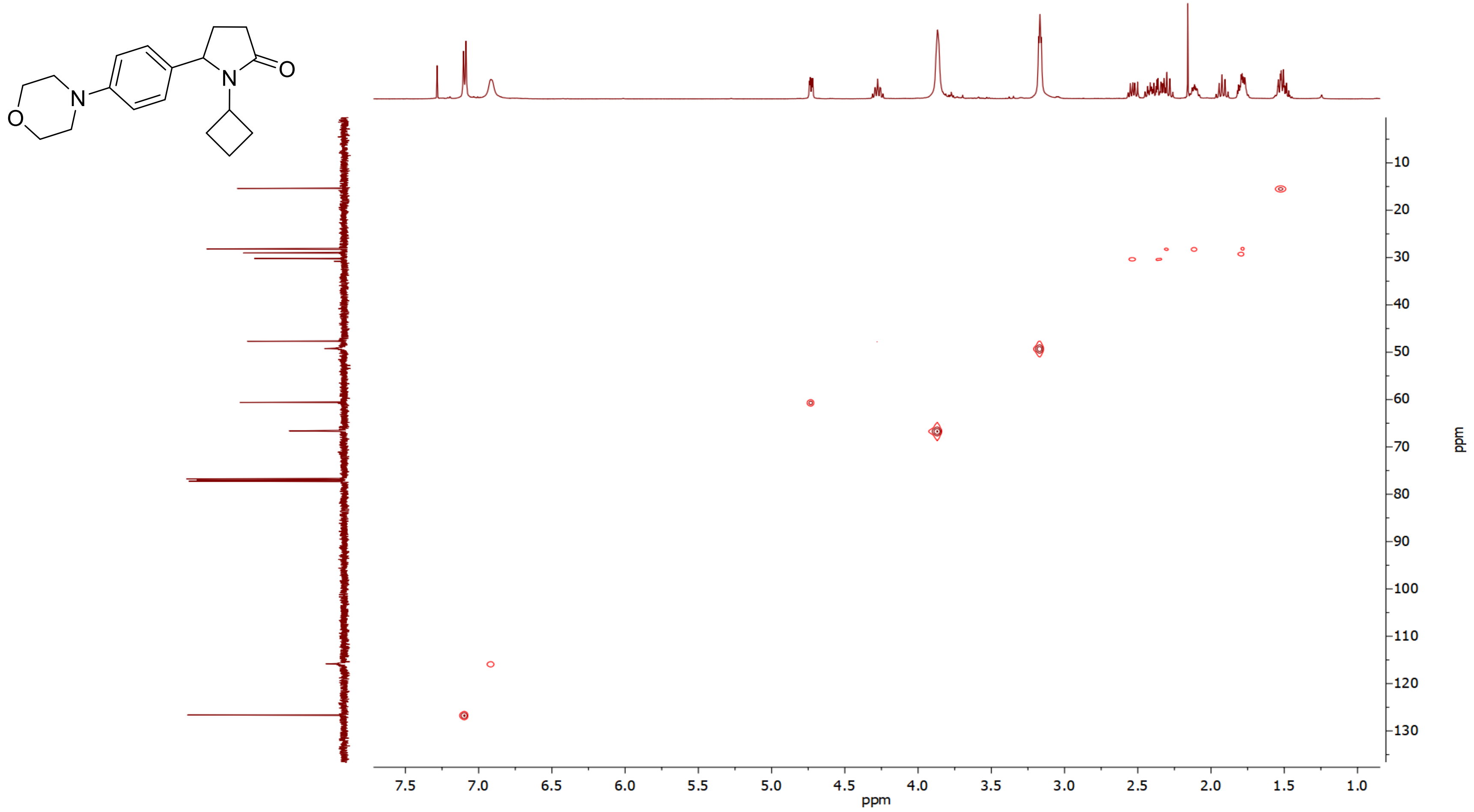

5-(3,4-Dimethoxyphenyl)-1-(prop-2-yn-1-yl)pyrrolidin-2-one (**3i**)

^{1}H NMR ($CDCl_3$, 400 MHz)

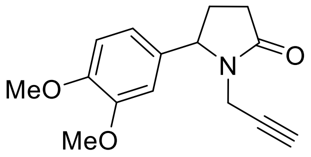

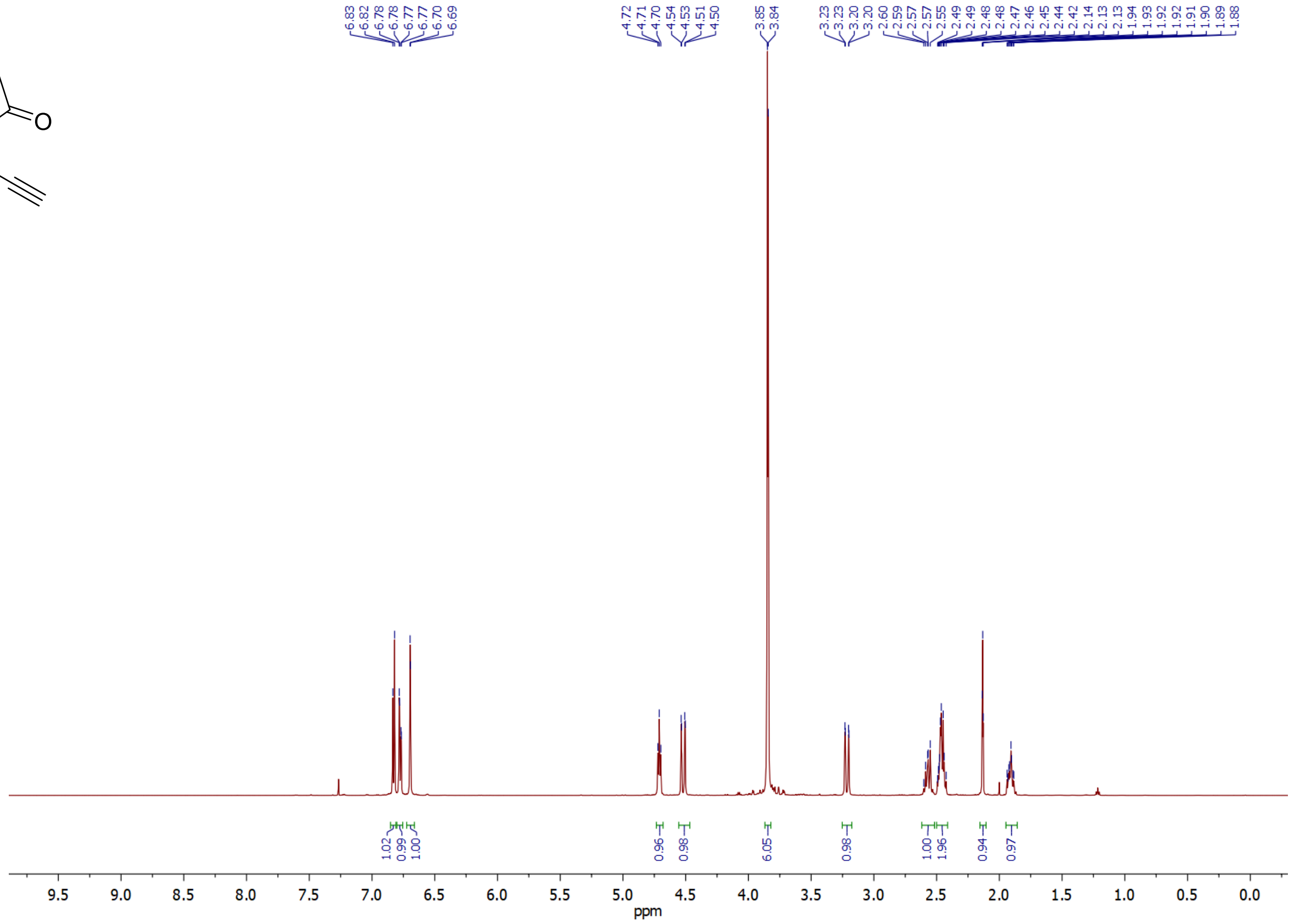

5-(3,4-Dimethoxyphenyl)-1-(prop-2-yn-1-yl)pyrrolidin-2-one (**3i**)

^{13}C NMR ($CDCl_3$, 100 MHz)

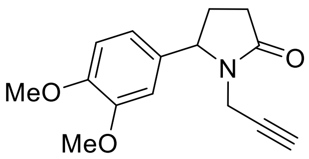

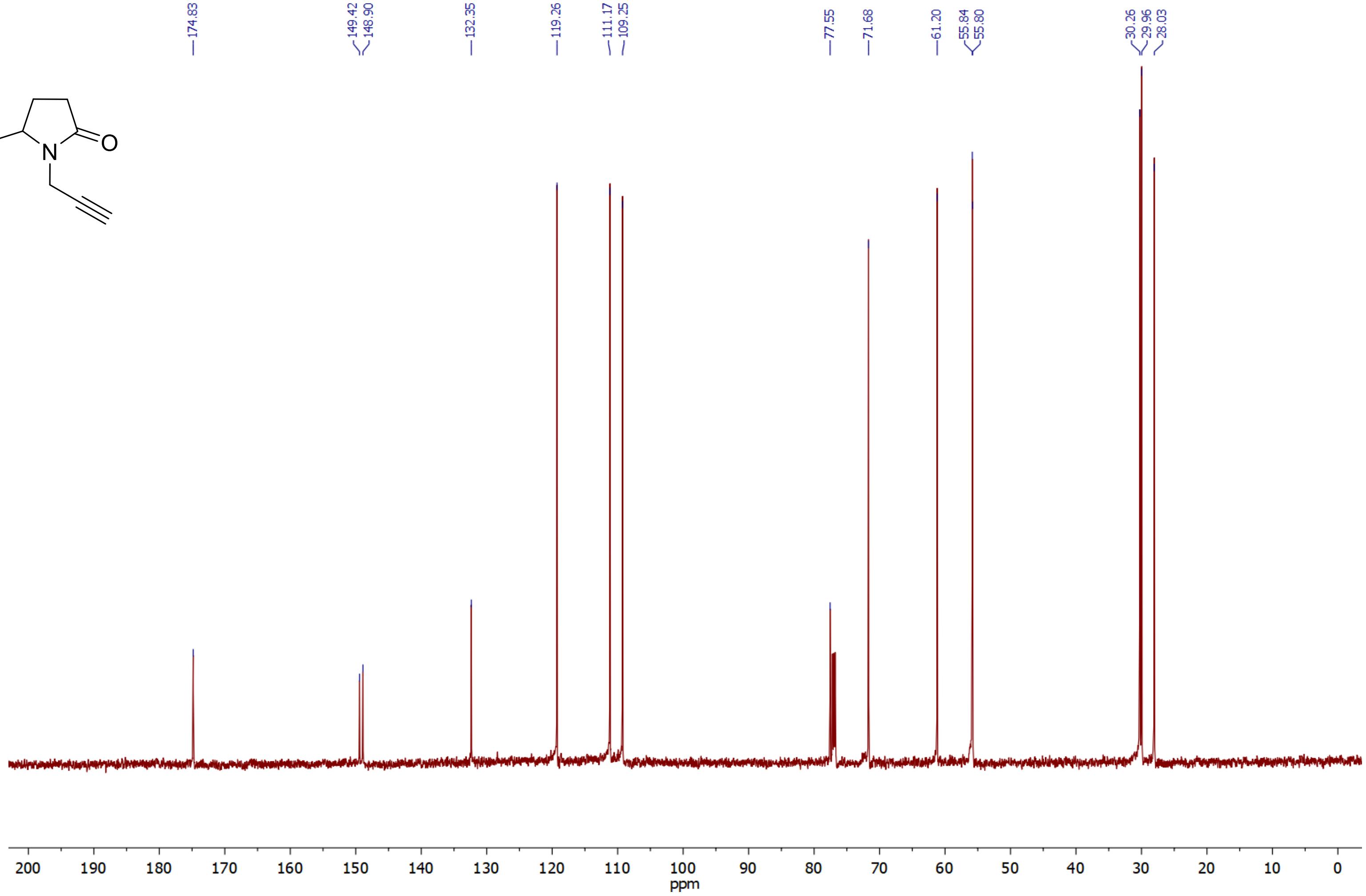

Dimethyl 2-[2-(3,5-dimethoxyphenyl)-2-(phenylamino)ethyl]malonate (**4a**)

^{1}H NMR ($CDCl_3$, 600 MHz)

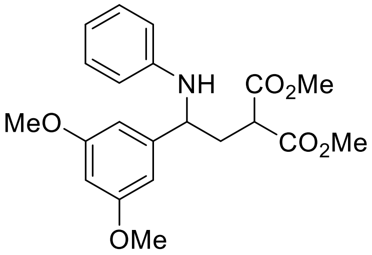

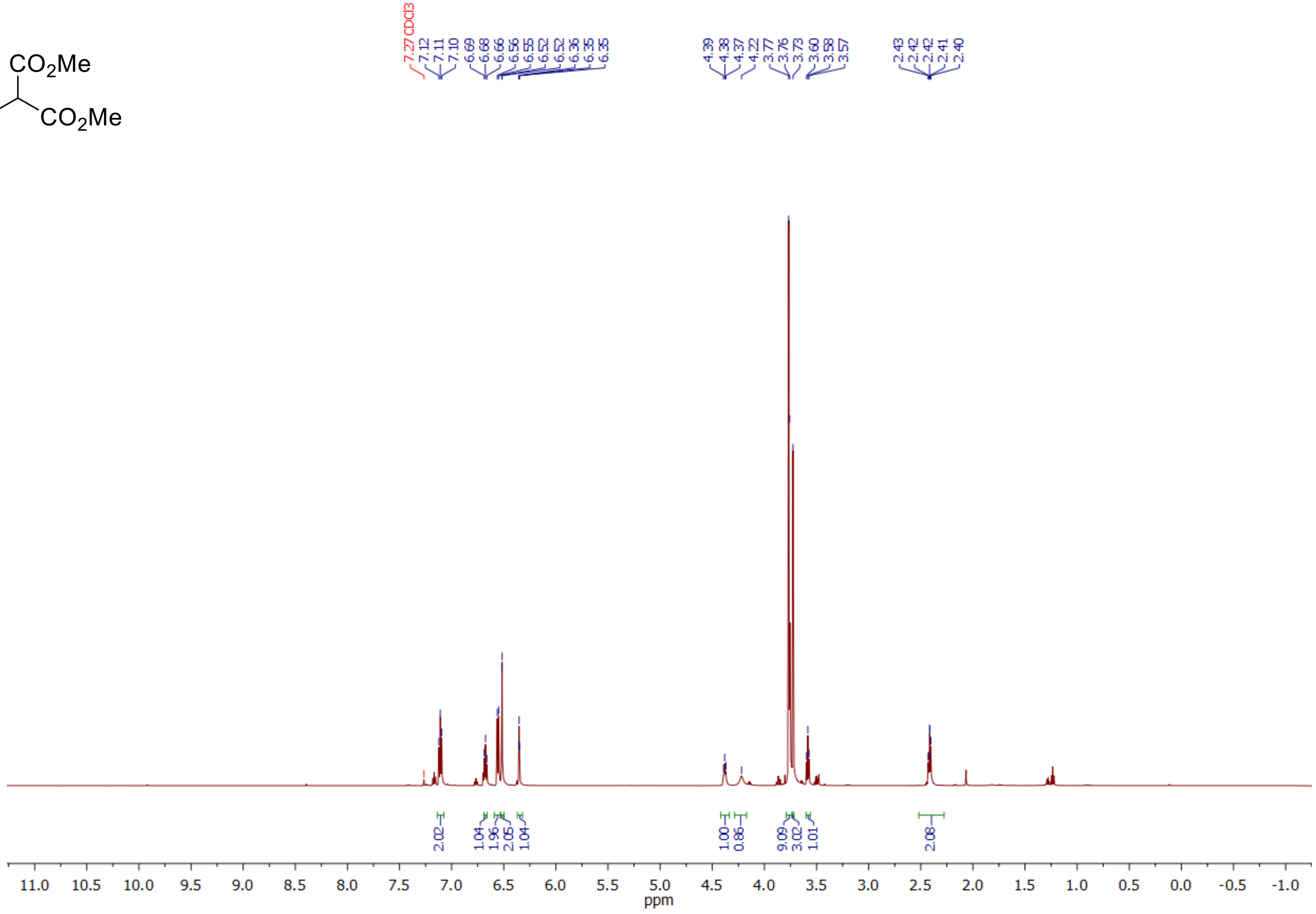

Dimethyl 2-[2-(3,5-dimethoxyphenyl)-2-(phenylamino)ethyl]malonate (**4a**)

^{13}C NMR ($CDCl_3$, 150 MHz)

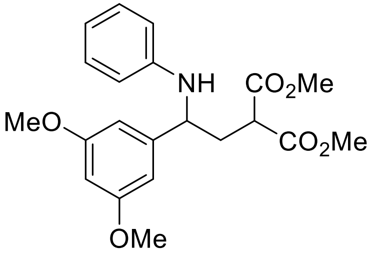

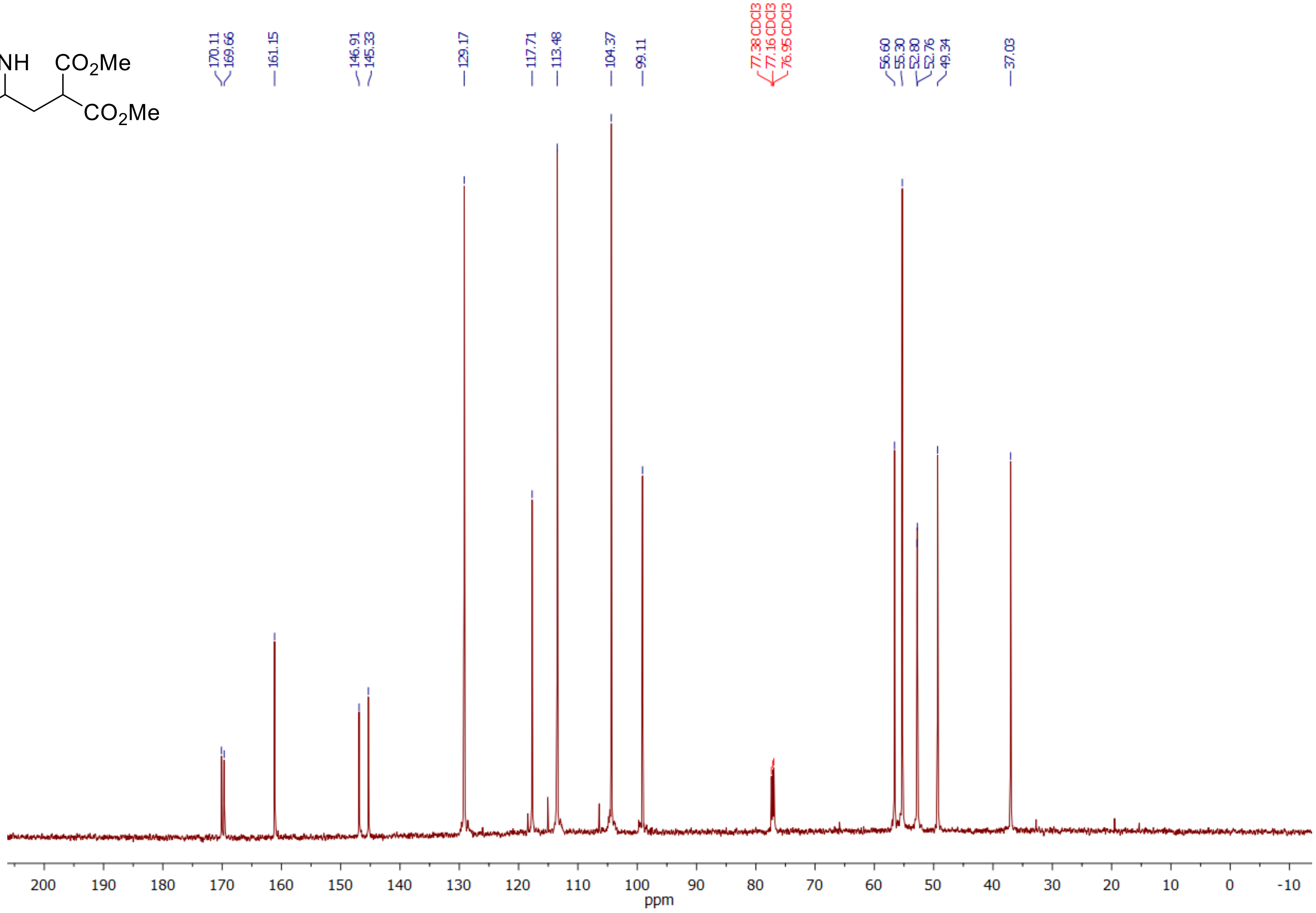

Dimethyl 2-{2-[(2-nitrophenyl)amino]-2-phenylethyl}malonate (4b)

^{1}H NMR ($CDCl_3$, 600 MHz)

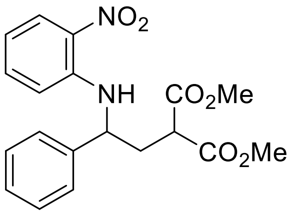

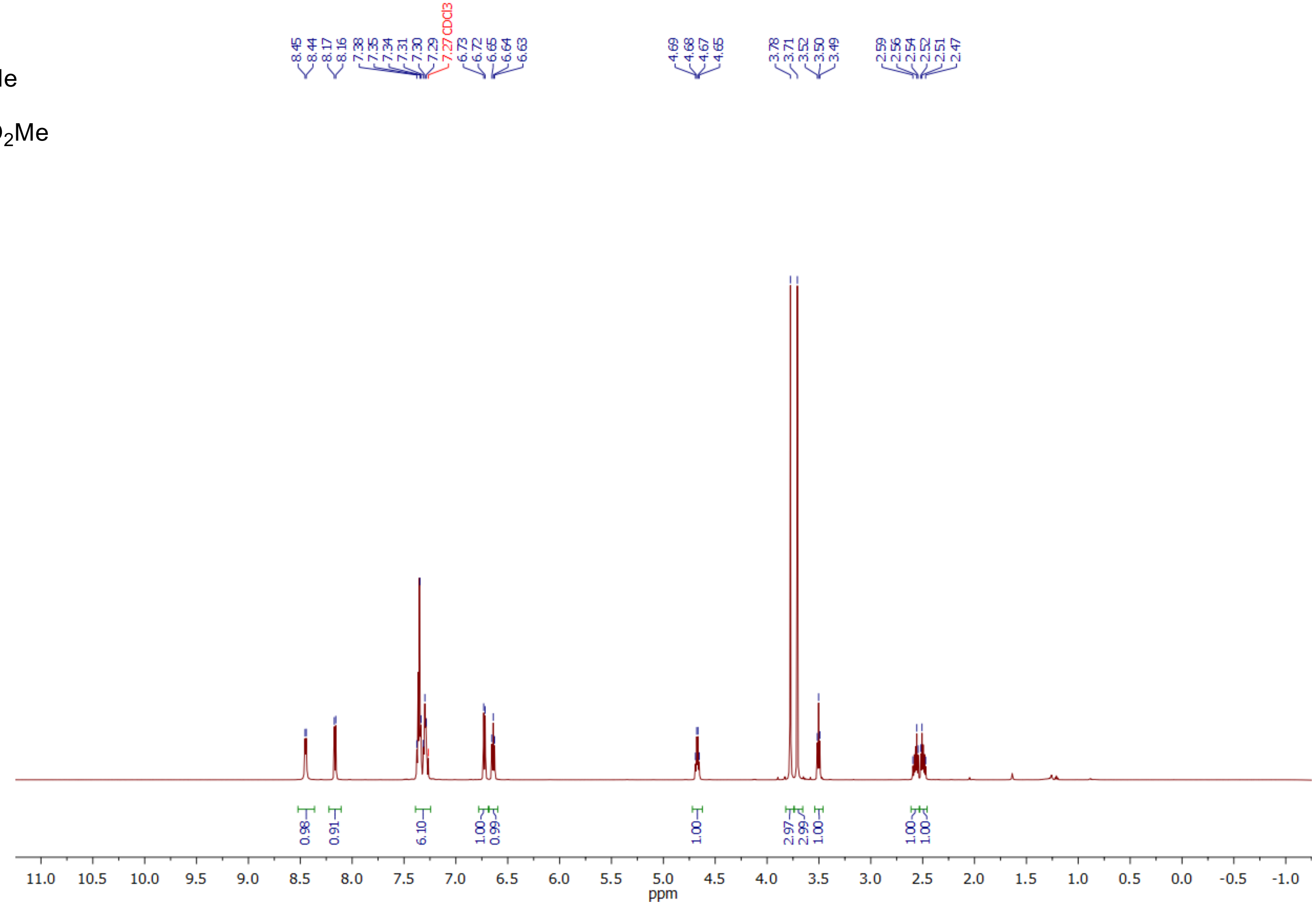

Dimethyl 2-{2-[(2-nitrophenyl)amino]-2-phenylethyl}malonate (4b)

^{13}C NMR ($CDCl_3$, 150 MHz)

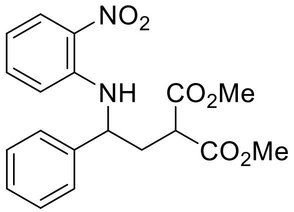

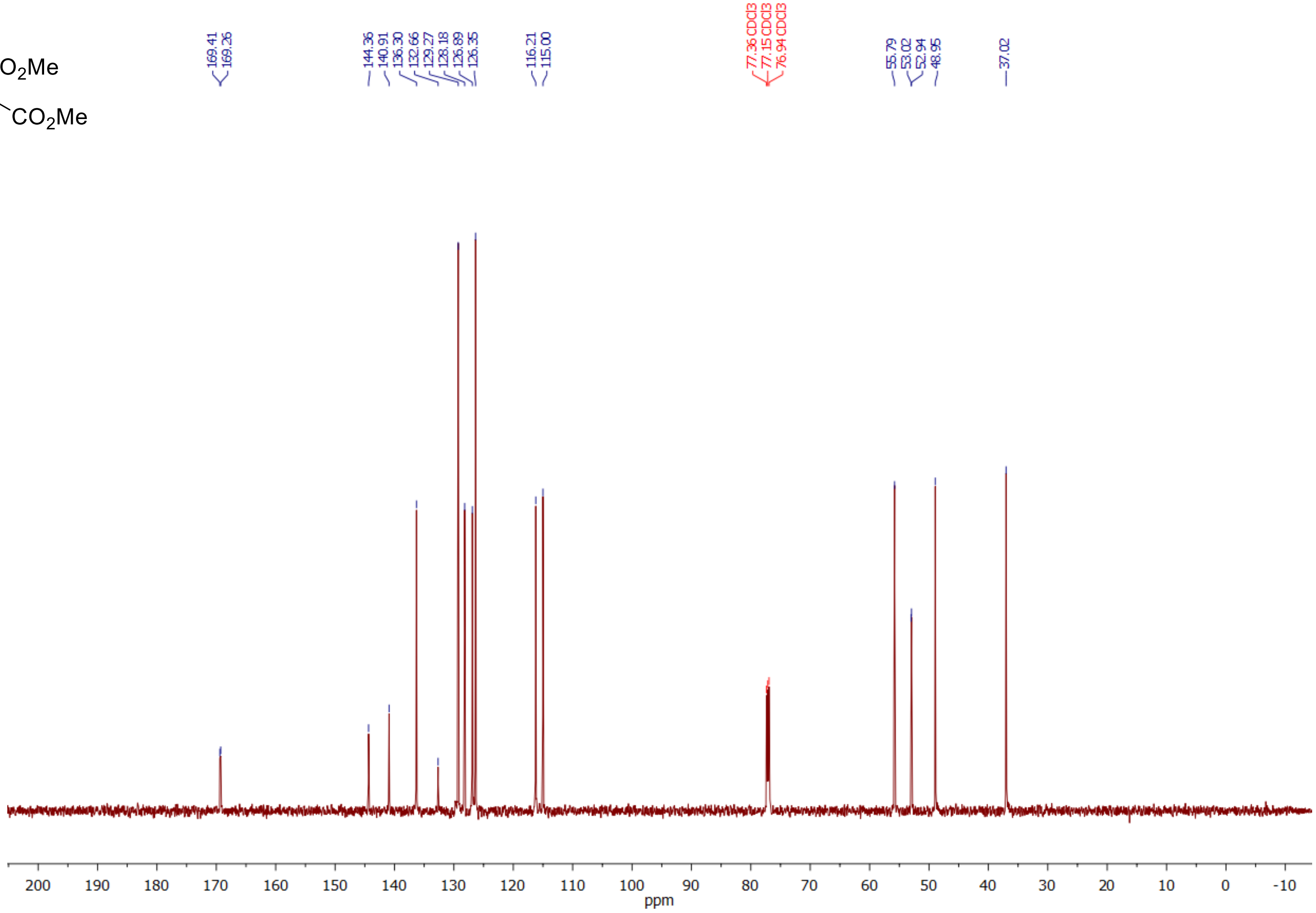

Dimethyl 2-{2-[(2-aminophenyl)amino]-2-(3,4-dimethoxyphenyl)ethyl}malonate (4c)

^{1}H NMR ($CDCl_3$, 400 MHz)

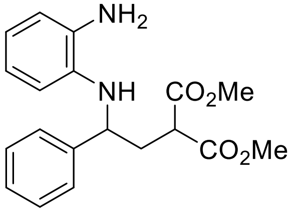

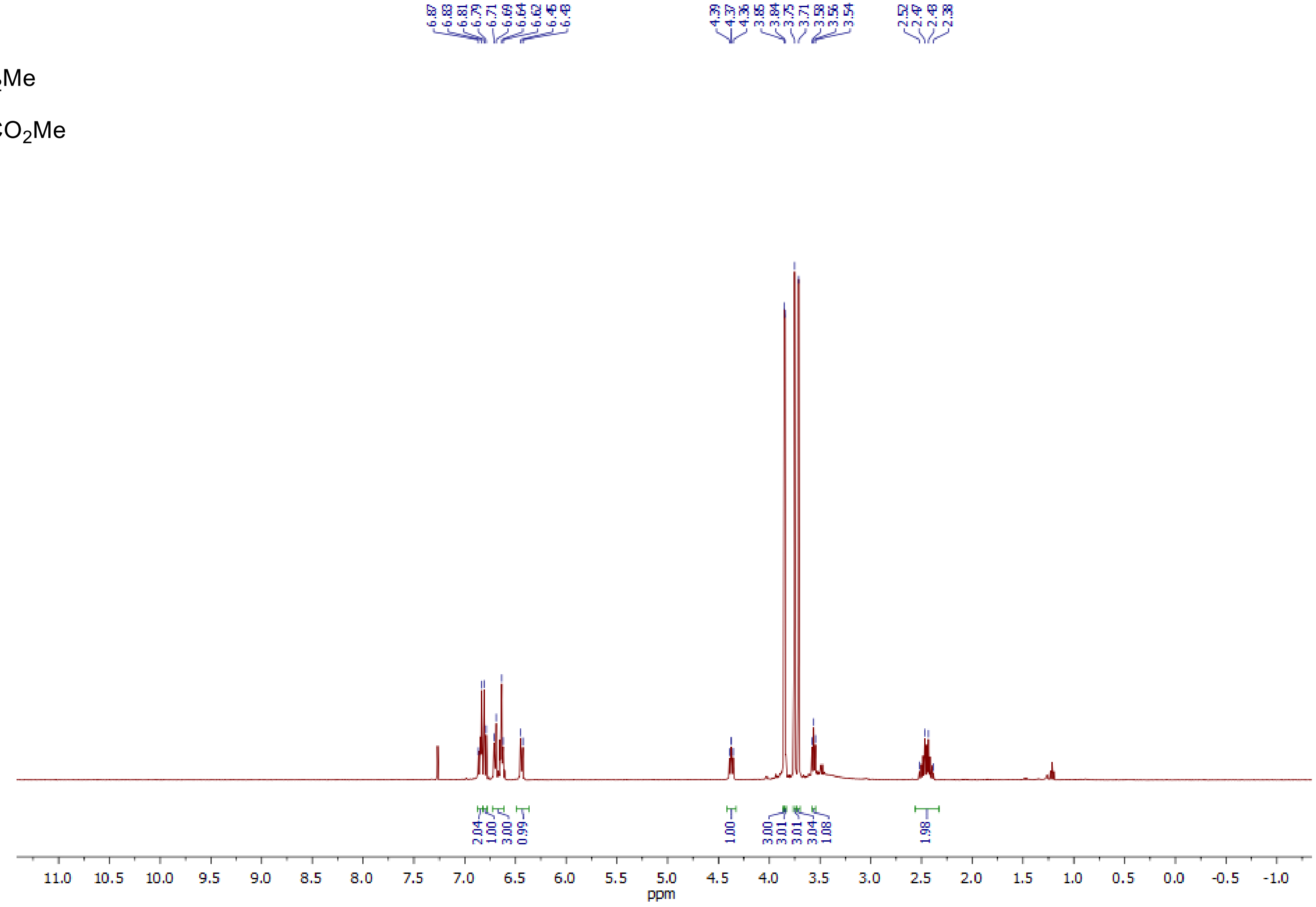

Dimethyl 2-{2-[(2-aminophenyl)amino]-2-(3,4-dimethoxyphenyl)ethyl}malonate (4c)

^{13}C NMR ($CDCl_3$, 100 MHz)

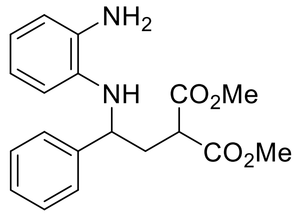

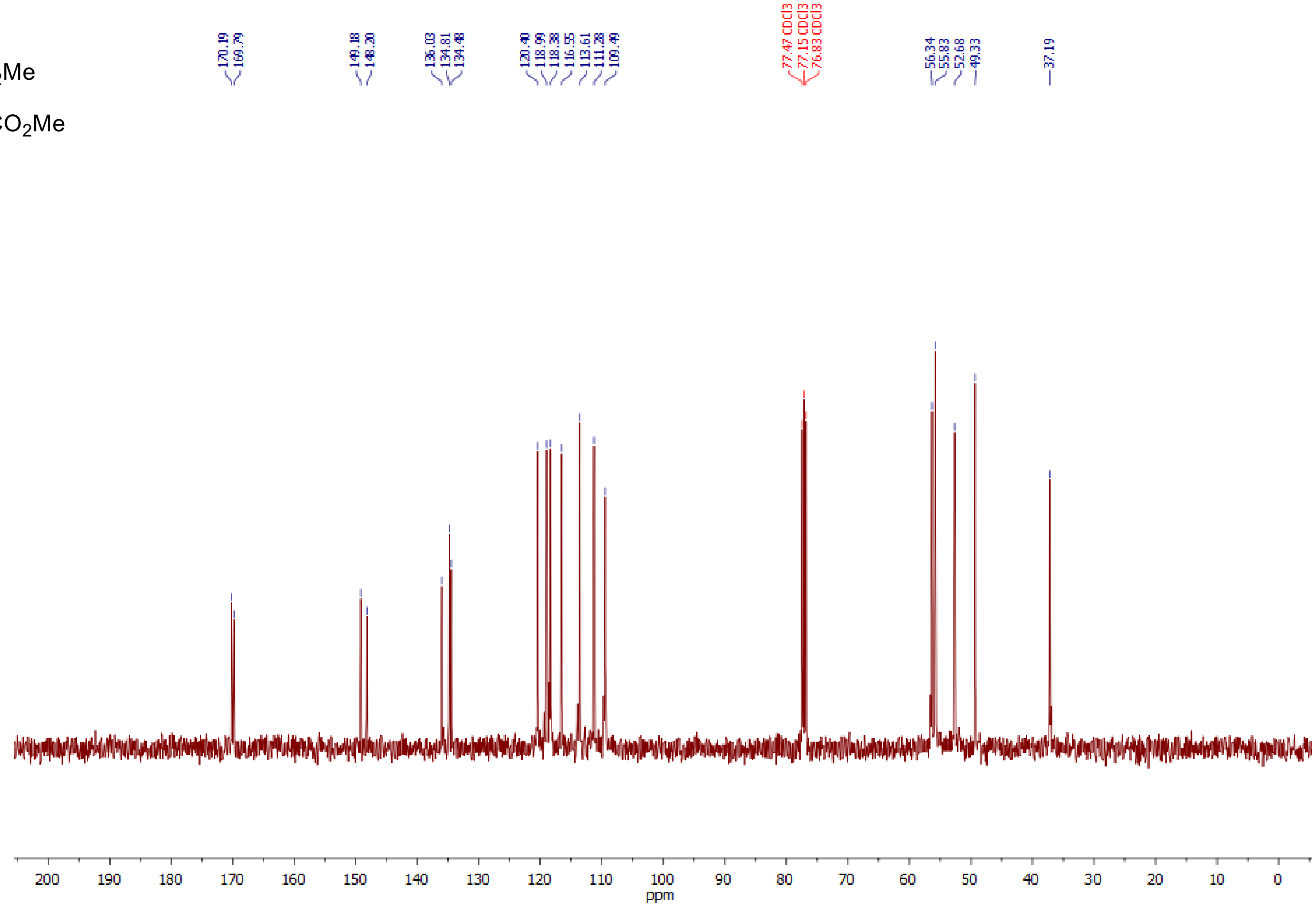

2-[2-Phenyl-2-(phenylamino)ethyl]malononitrile (4d)

^{1}H NMR ($CDCl_3$, 600 MHz)

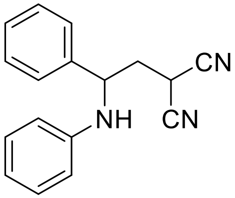

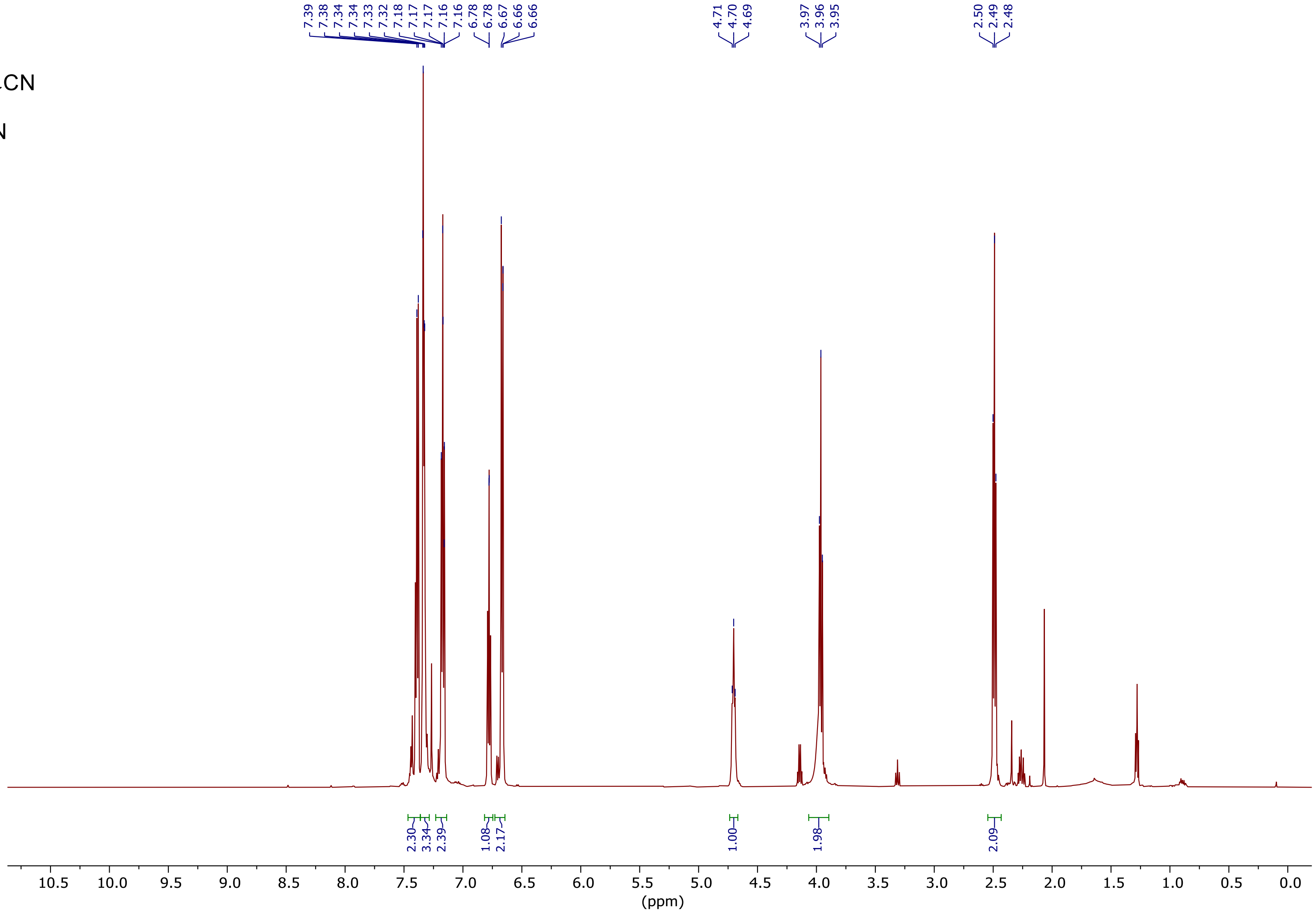

2-[2-Phenyl-2-(phenylamino)ethyl]malononitrile (4d)

^{13}C NMR ($CDCl_3$, 150 MHz)

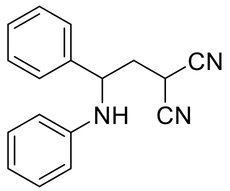

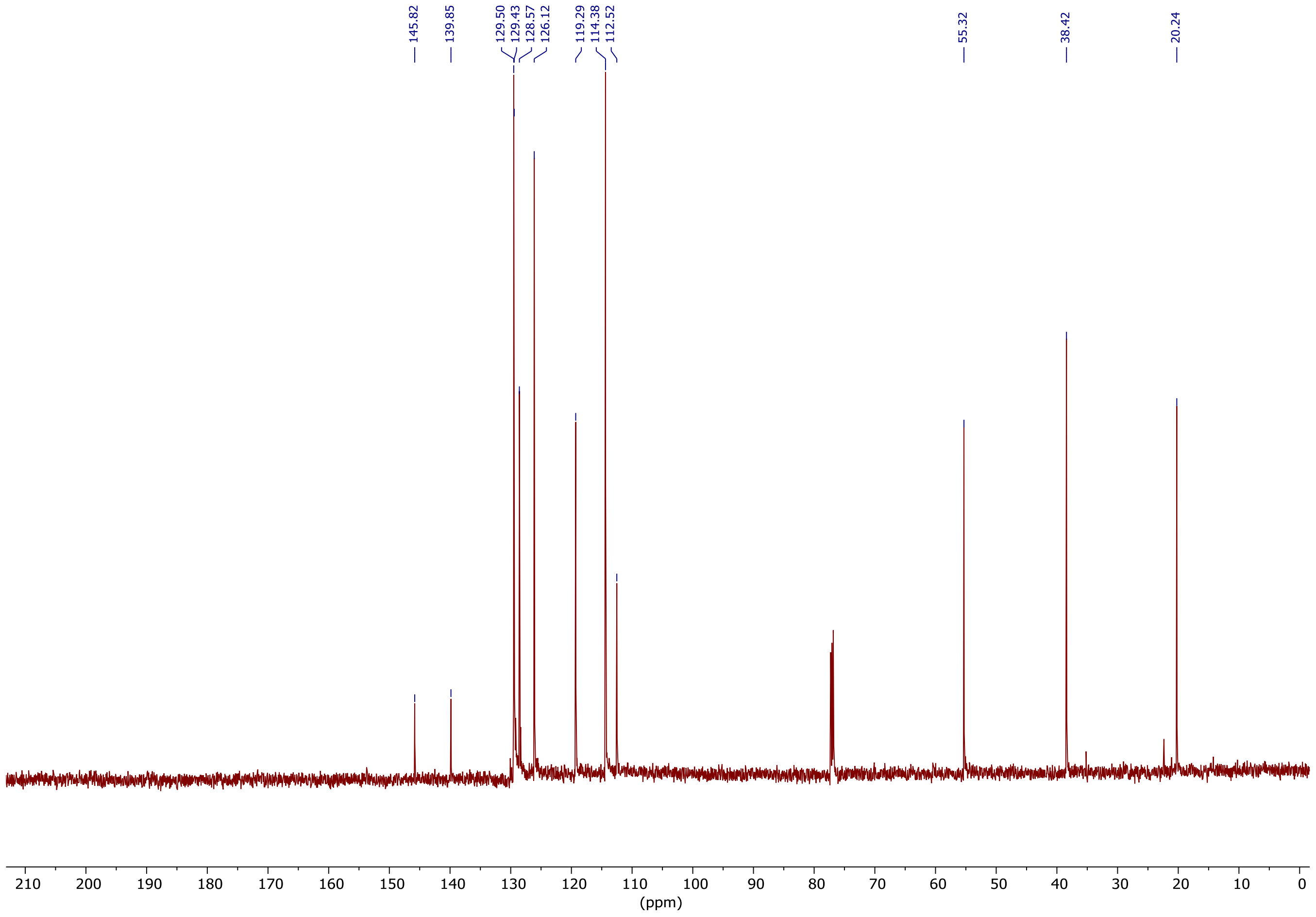

2-[2-Phenyl-2-(phenylamino)ethyl]malononitrile (4d)

HSQC ^{1}H-^{13}C ($CDCl_3$)

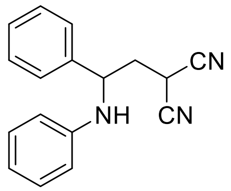

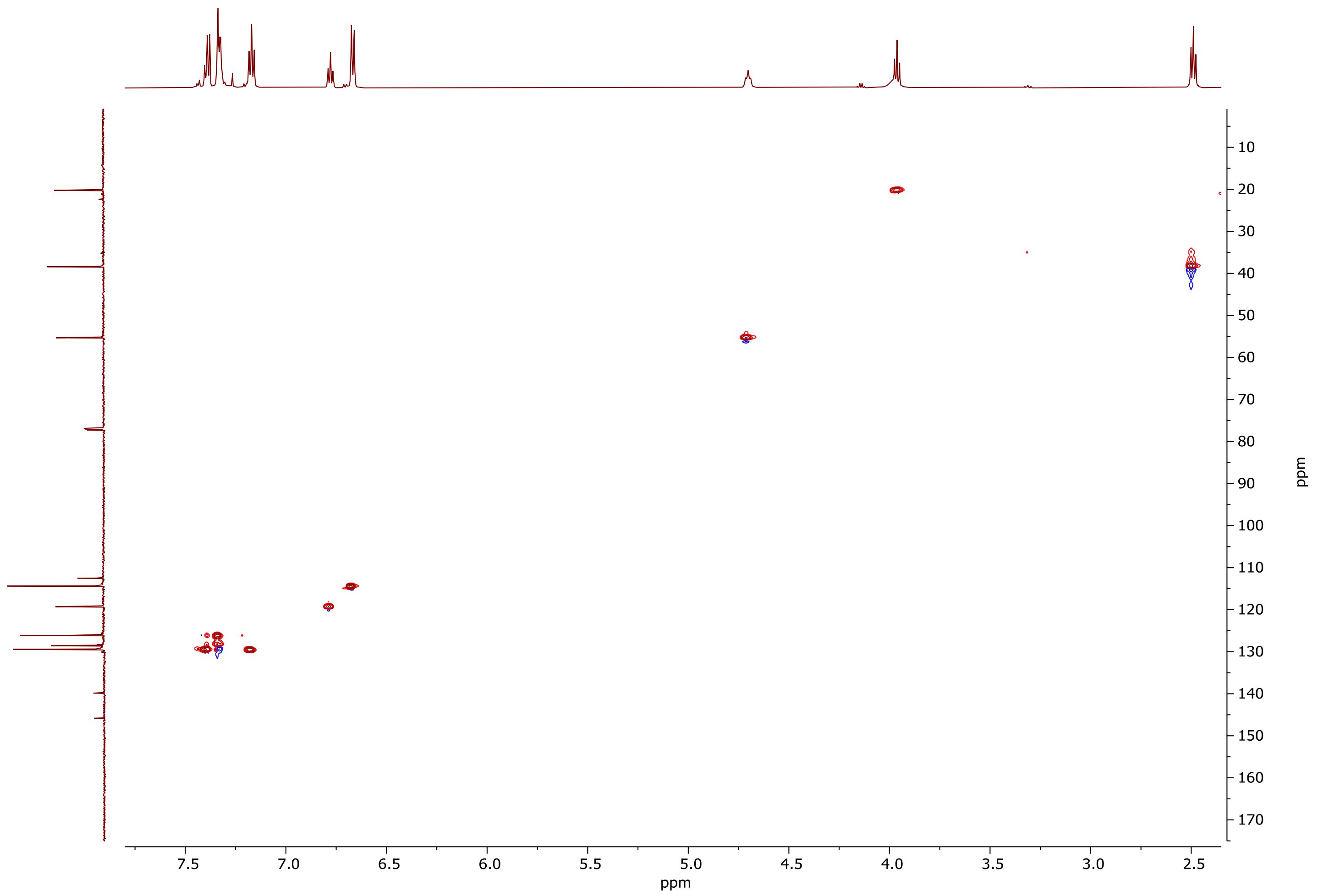

2-[2-Phenyl-2-(phenylamino)ethyl]malononitrile (4d)

HMBC ^{1}H-^{13}C ($CDCl_3$)

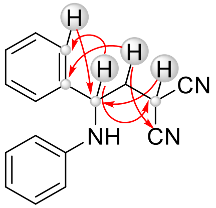

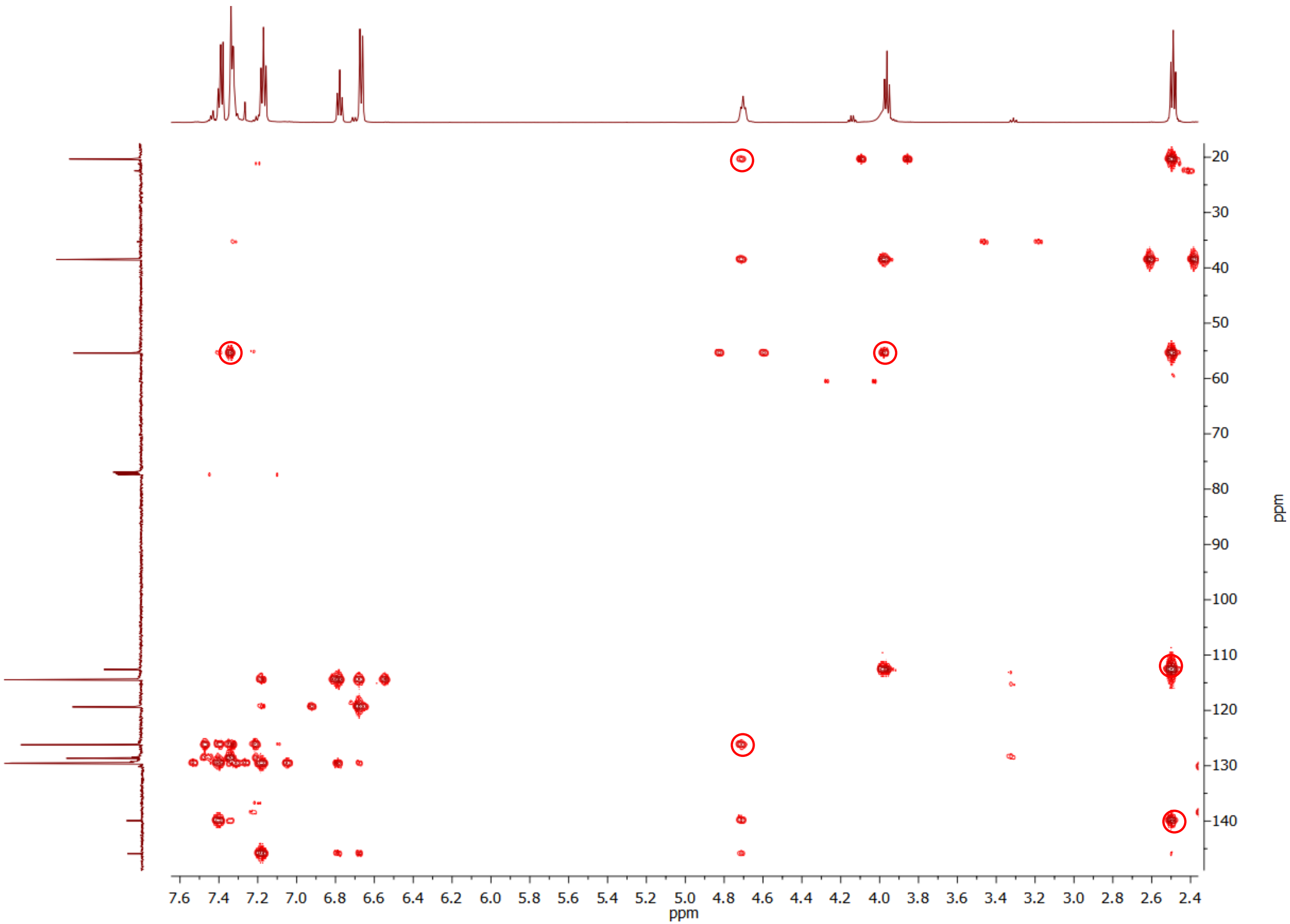

2-{2-[(2-Bromo-4-methylphenyl)amino]-2-phenylethyl}malononitrile (4e)

^{1}H NMR (CD_3OD, 600 MHz)

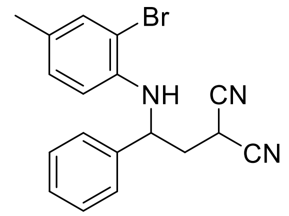

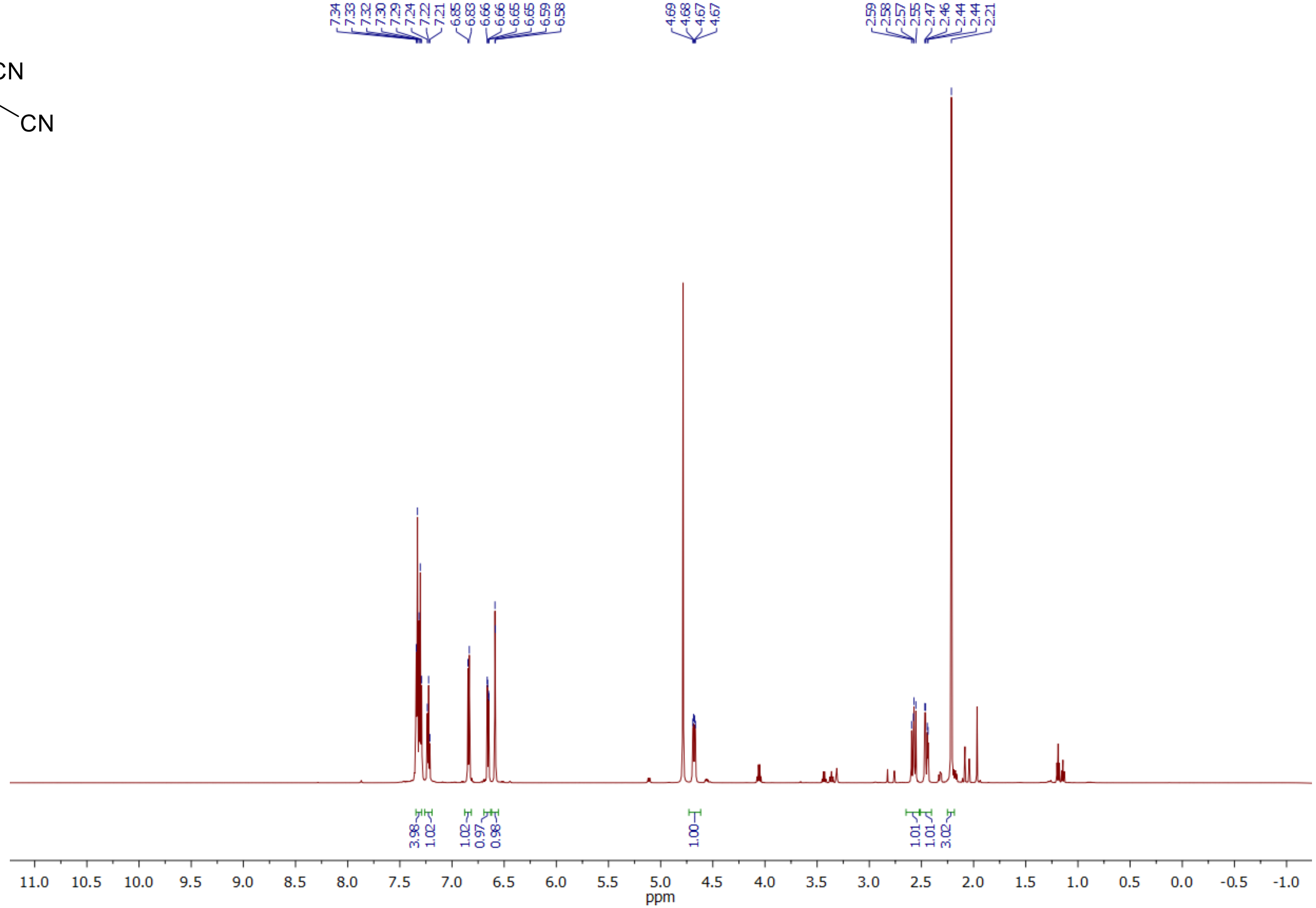

2-{2-[(2-Bromo-4-methylphenyl)amino]-2-phenylethyl}malononitrile (**4e**)

^{13}C NMR (CD_3OD, 150 MHz)

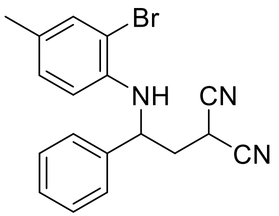

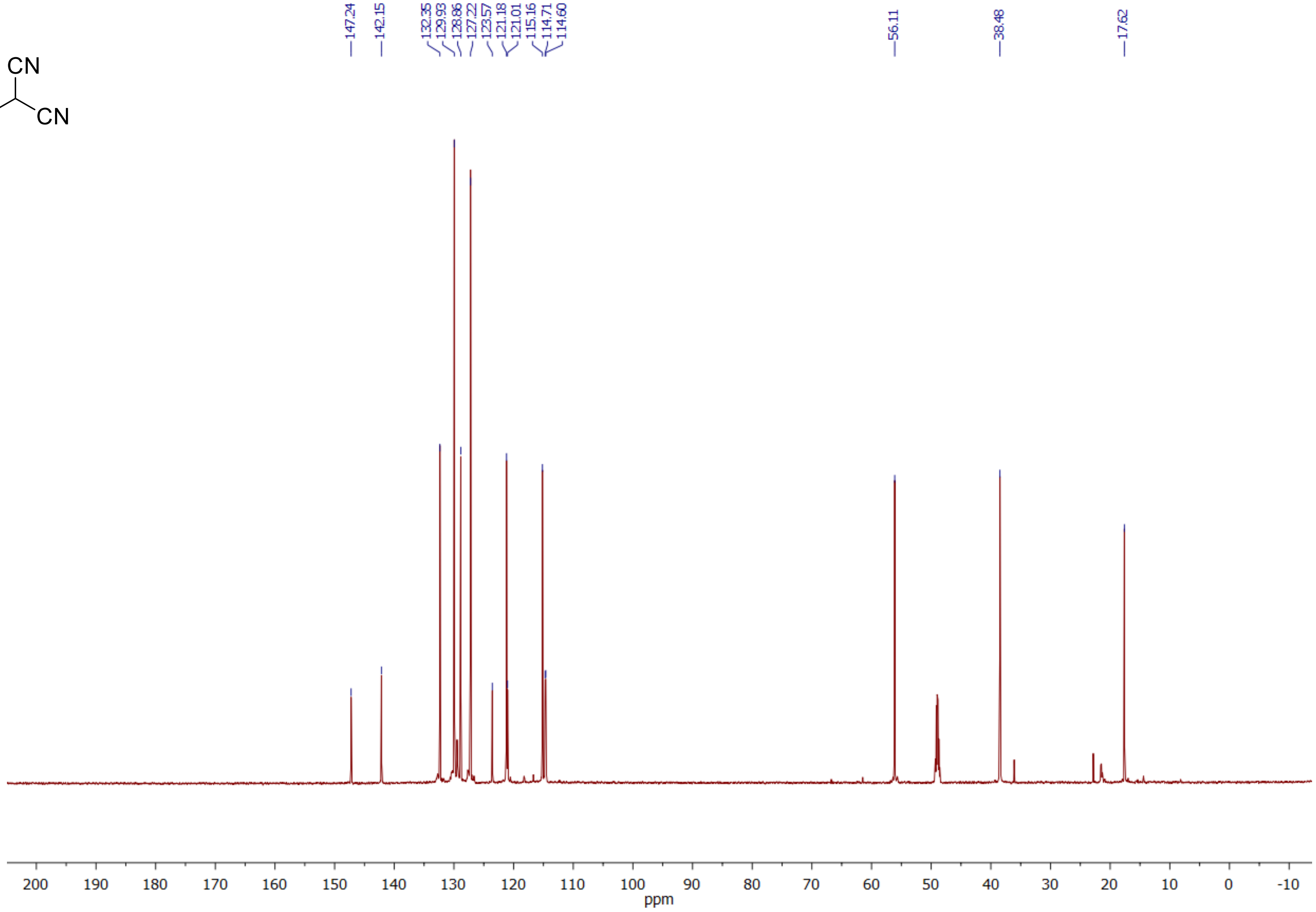

2-**{2-[(2-Bromo-4-methylphenyl)amino]-2-phenylethyl}malononitrile** (**4e**)

HSQC ^1H-^{13}C (CD_3OD)

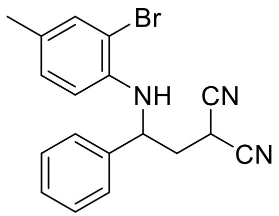

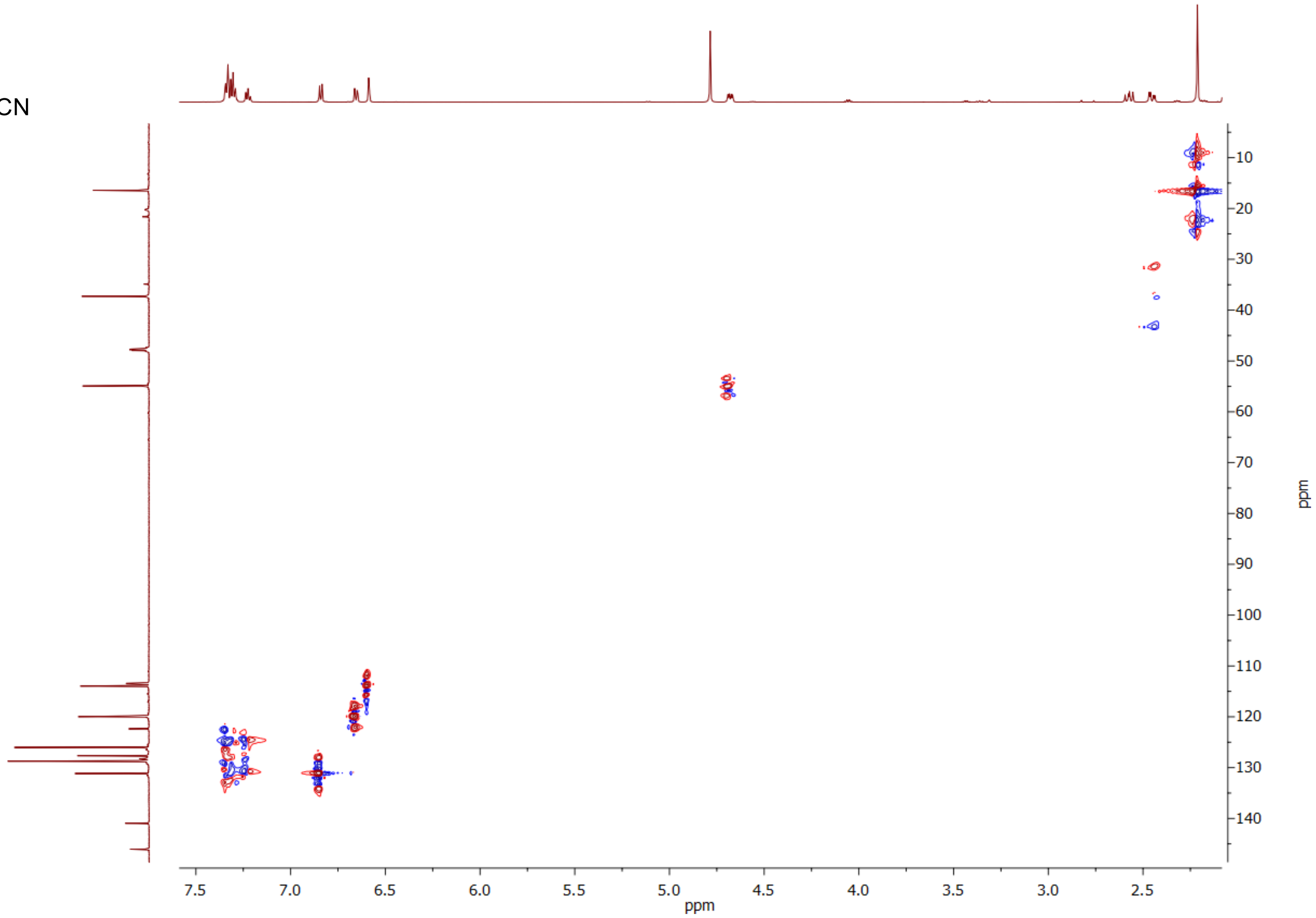

2-{2-[(2-Bromo-4-methylphenyl)amino]-2-phenylethyl}malononitrile (4e)

HMBC ^{1}H-^{13}C (CD_3OD)

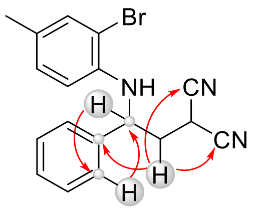

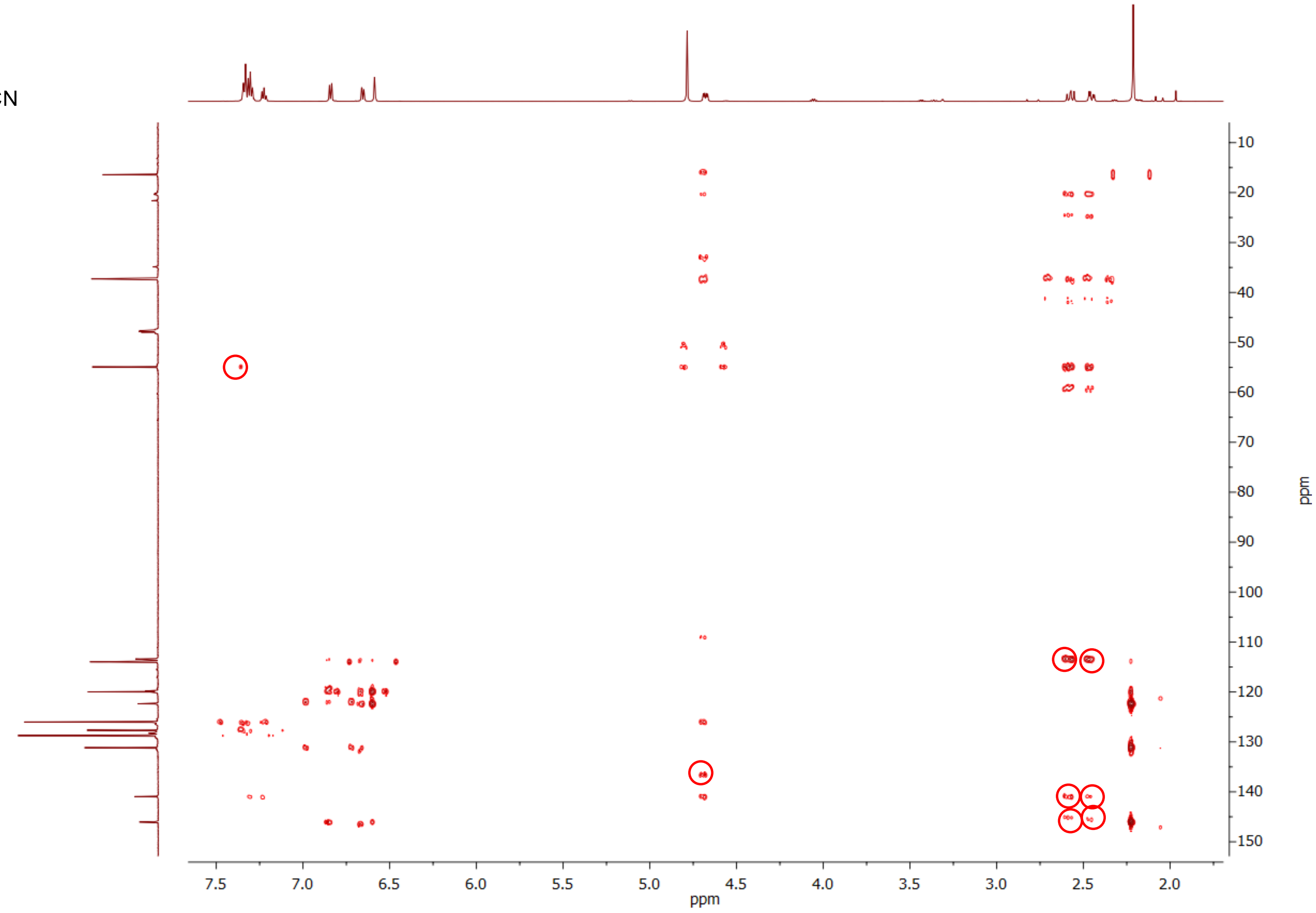

(*3aRS,5RS*)-5-Phenyl-3,3a,4,5-tetrahydropyrrolo[1,2-a]quinoline-1(2*H*)-one (5)

^{1}H NMR ($CDCl_3$, 600 MHz)

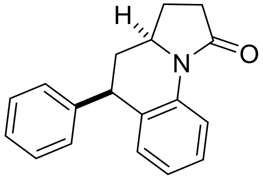

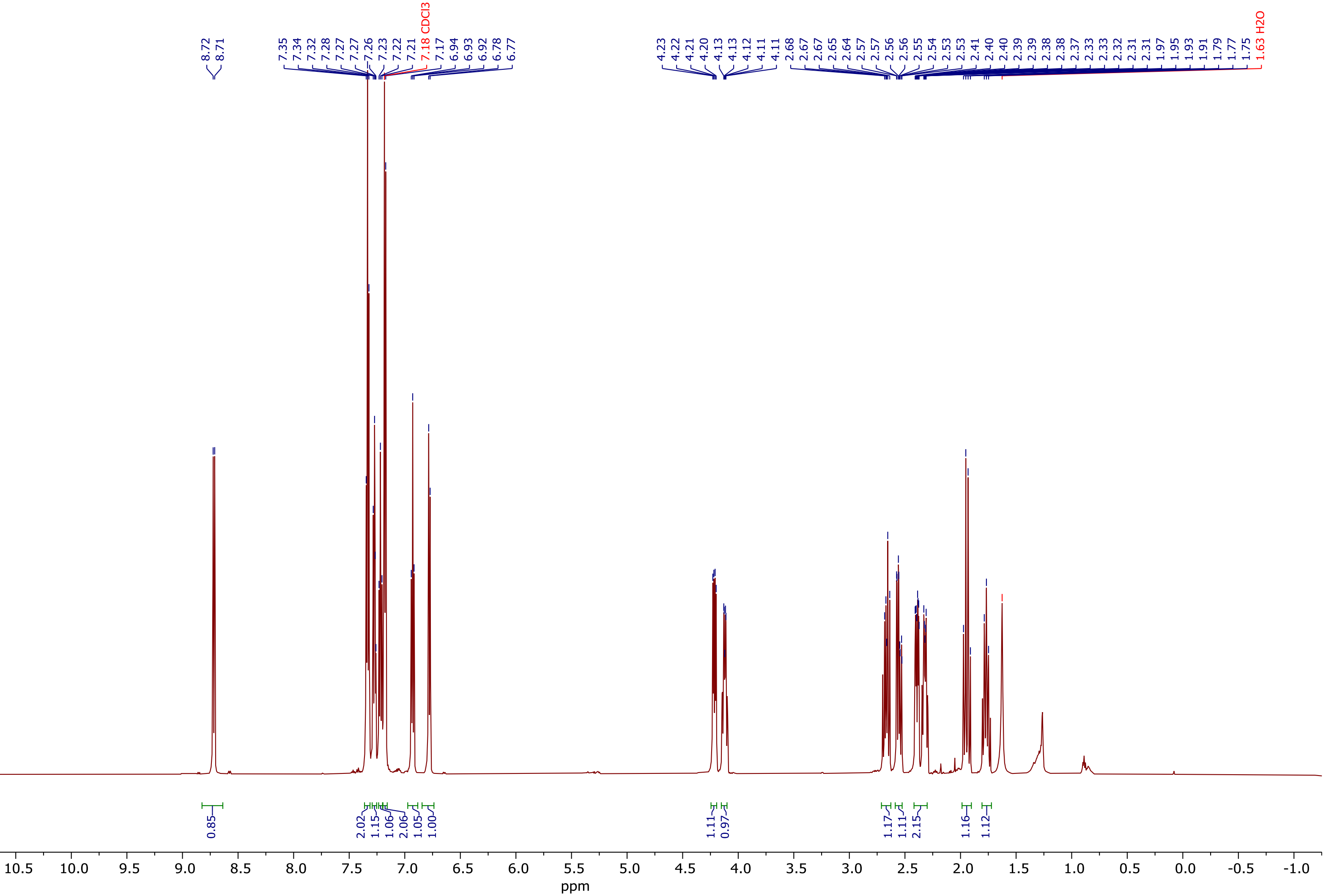

(*3aRS,5RS*)-5-Phenyl-3,3a,4,5-tetrahydropyrrolo[1,2-a]quinoline-1(2*H*)-one (5)

^{13}C NMR ($CDCl_3$, 150 MHz)

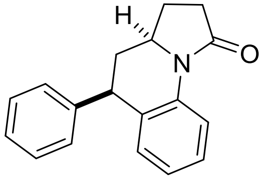

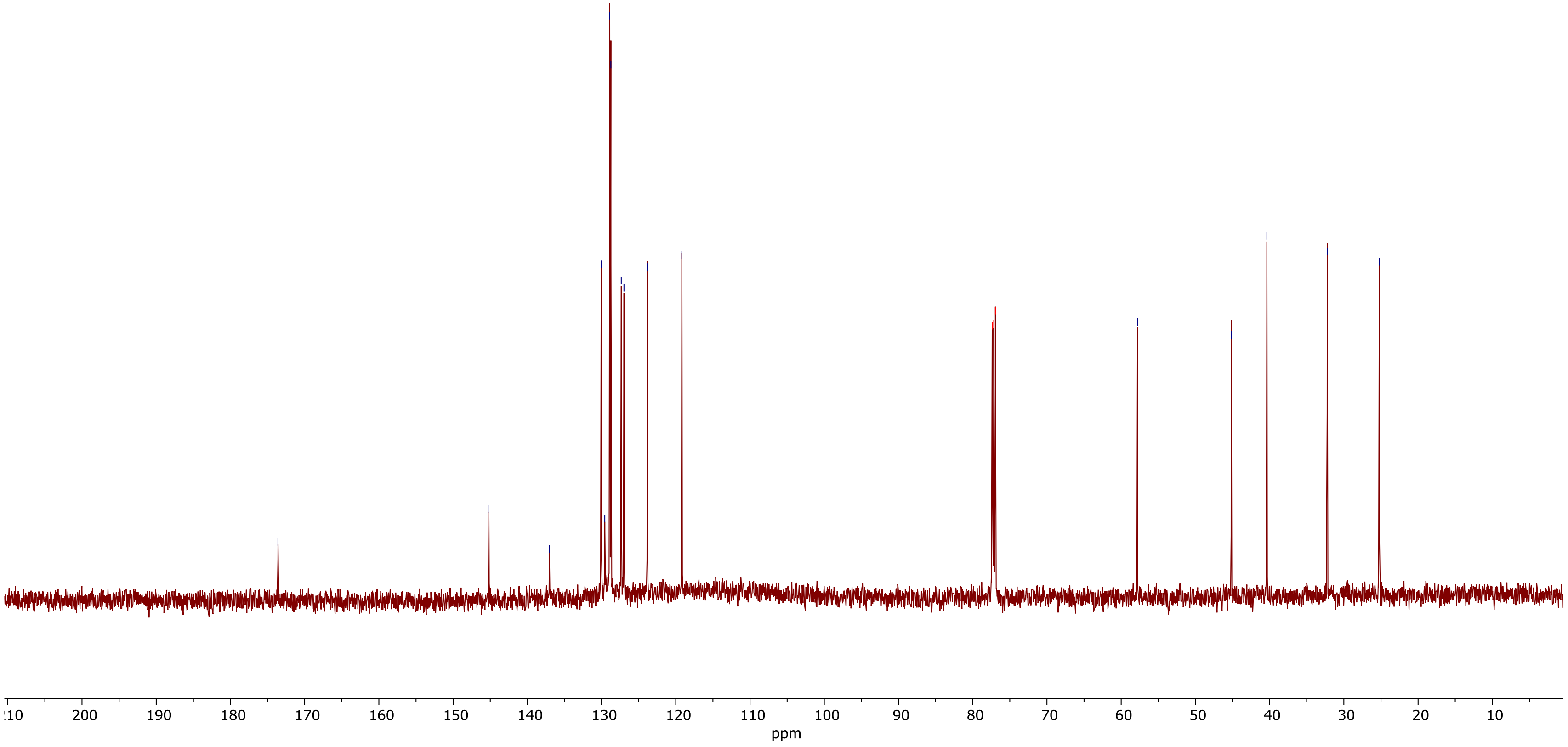

(*3aRS,5RS*)-5-Phenyl-3,3a,4,5-tetrahydropyrrolo[1,2-a]quinoline-1(2*H*)-one (5)

HSQC ^1H-^{13}C ($CDCl_3$)

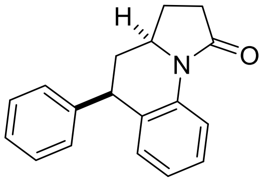

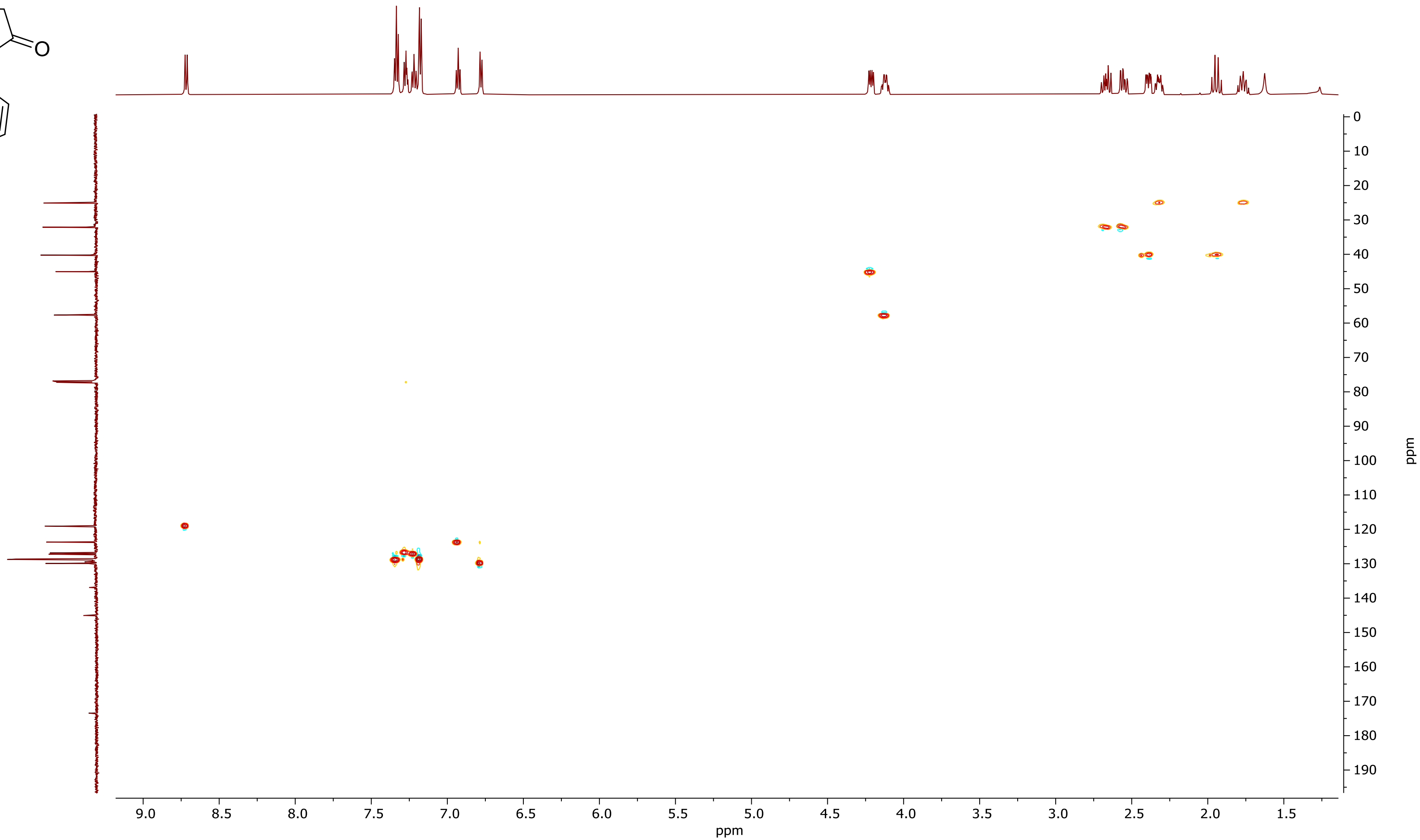

(*3aRS,5RS*)-5-Phenyl-3,3a,4,5-tetrahydropyrrolo[1,2-a]quinoline-1(2*H*)-one (5)

HMBC ^{1}H-^{13}C ($CDCl_3$)

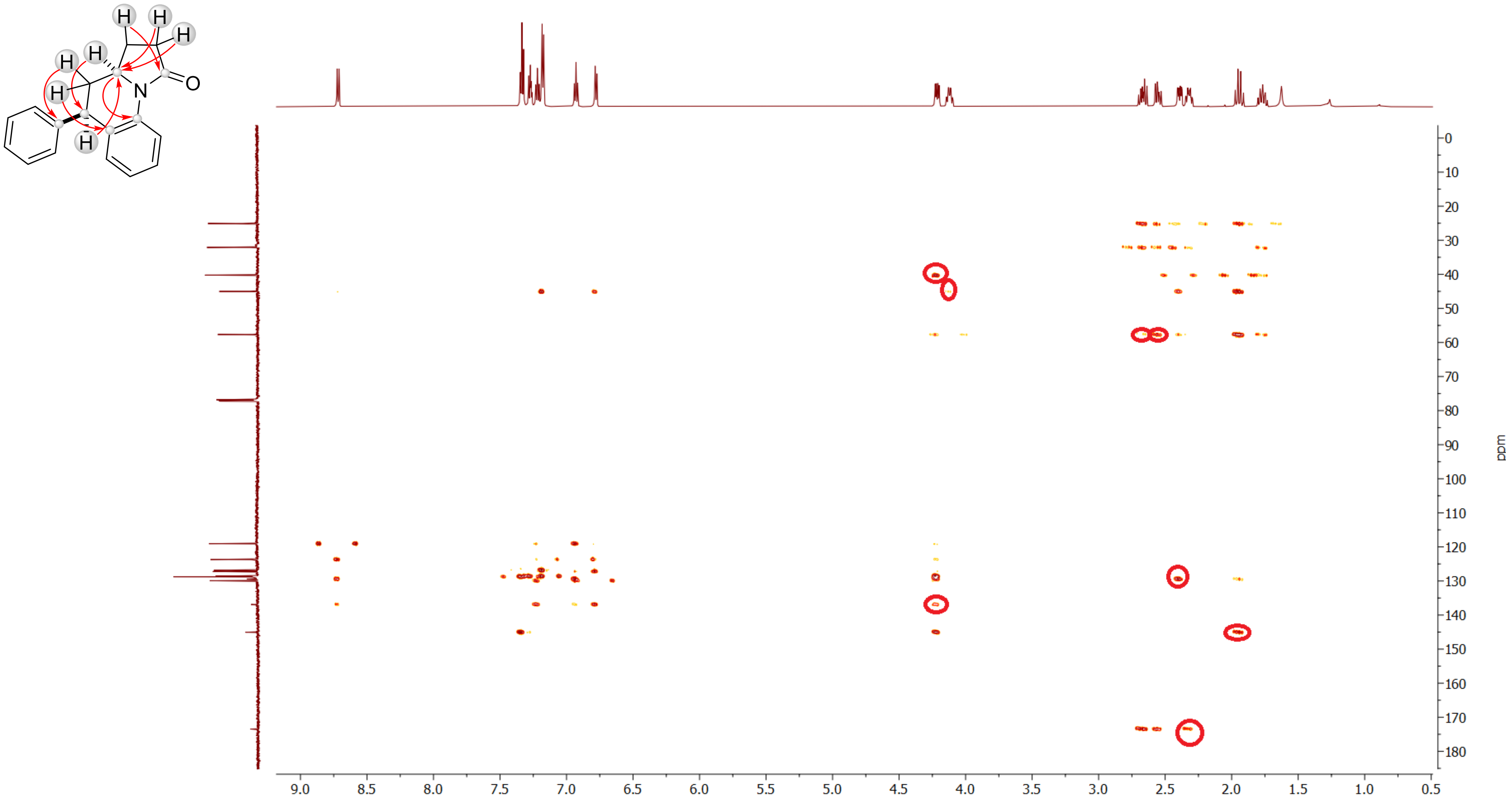

(*3aRS,5RS*)-5-Phenyl-3,3a,4,5-tetrahydropyrrolo[1,2-a]quinoline-1(2*H*)-one (5)

NOESY ($CDCl_3$) ($CDCl_3$)

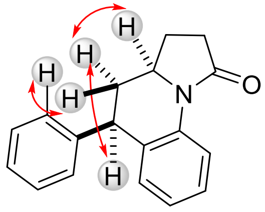

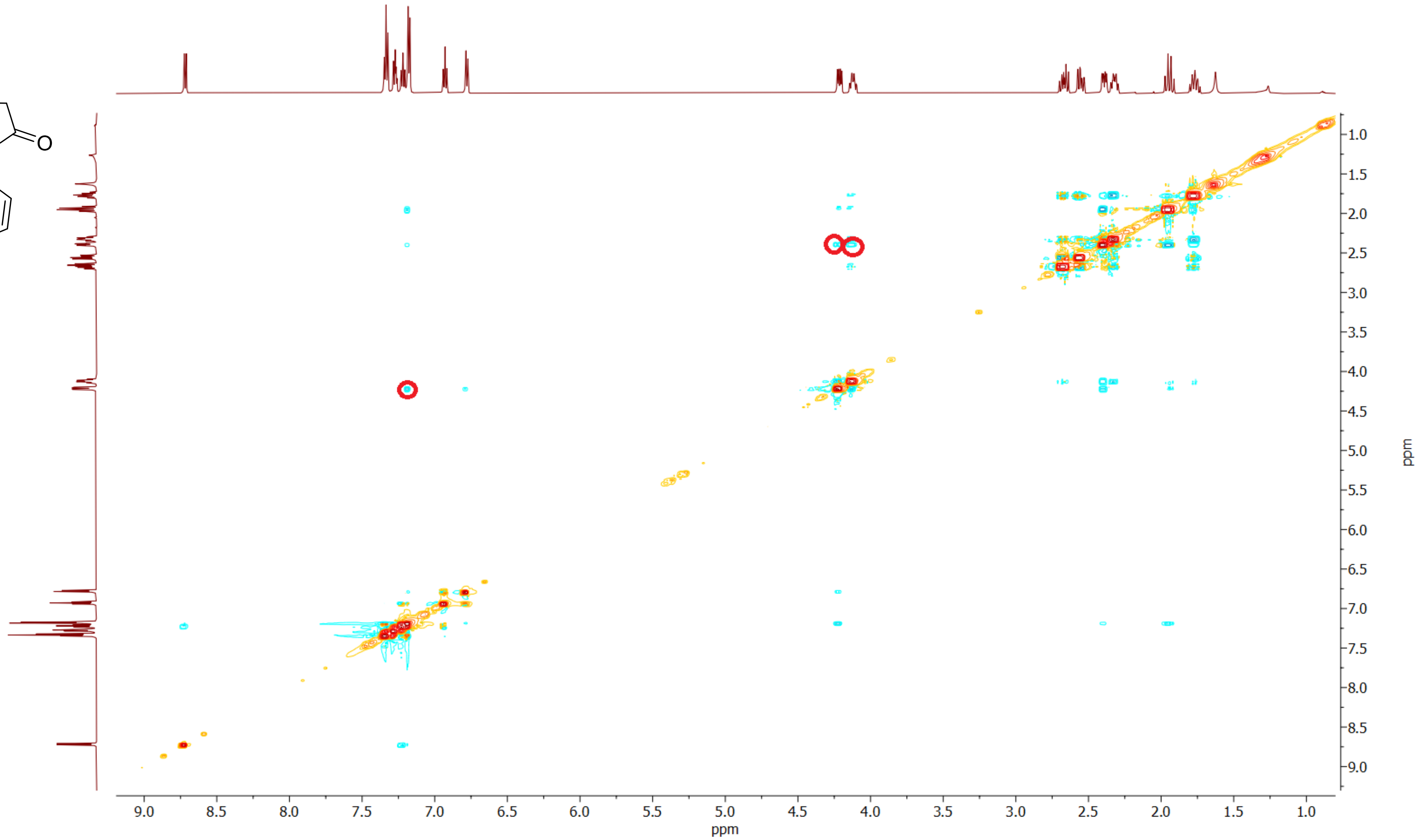

HPLC report for racemic 1-(4-methoxyphenyl)-5-(*p*-tolyl)pyrrolidin-2-one (2d)

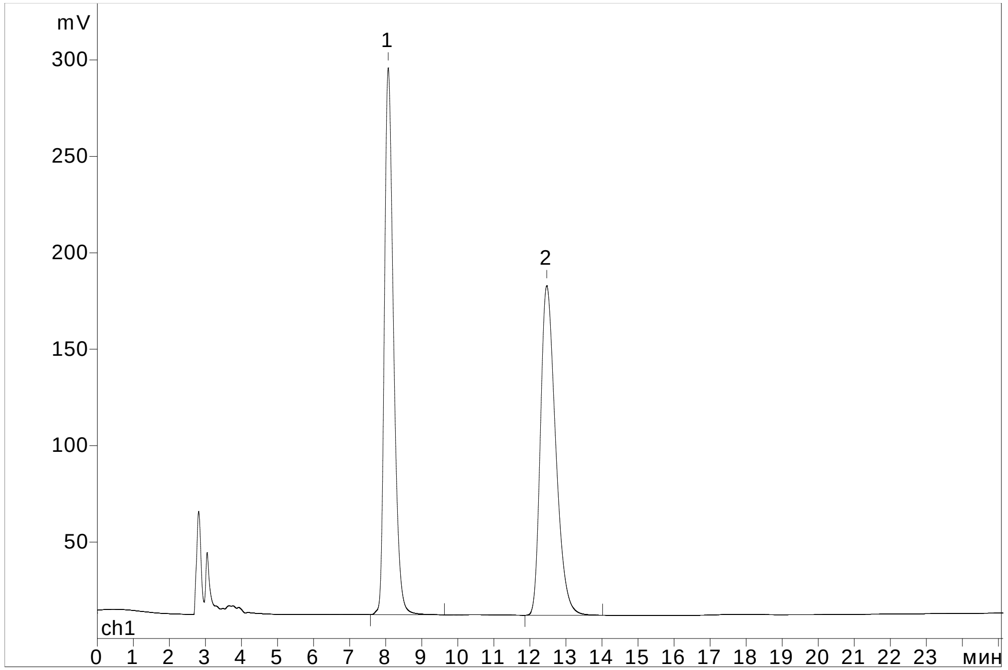

No	Retention	Area	Area	Name
	мин	mV*сек	%	
1	8.07	4999.208	50.04	
2	12.47	4990.657	49.96	
2	25.15	9989.865	100.00	

HPLC report for enantioenriched 1-(4-methoxyphenyl)-5-(*p*-tolyl)pyrrolidin-2-one (*R*)-2d

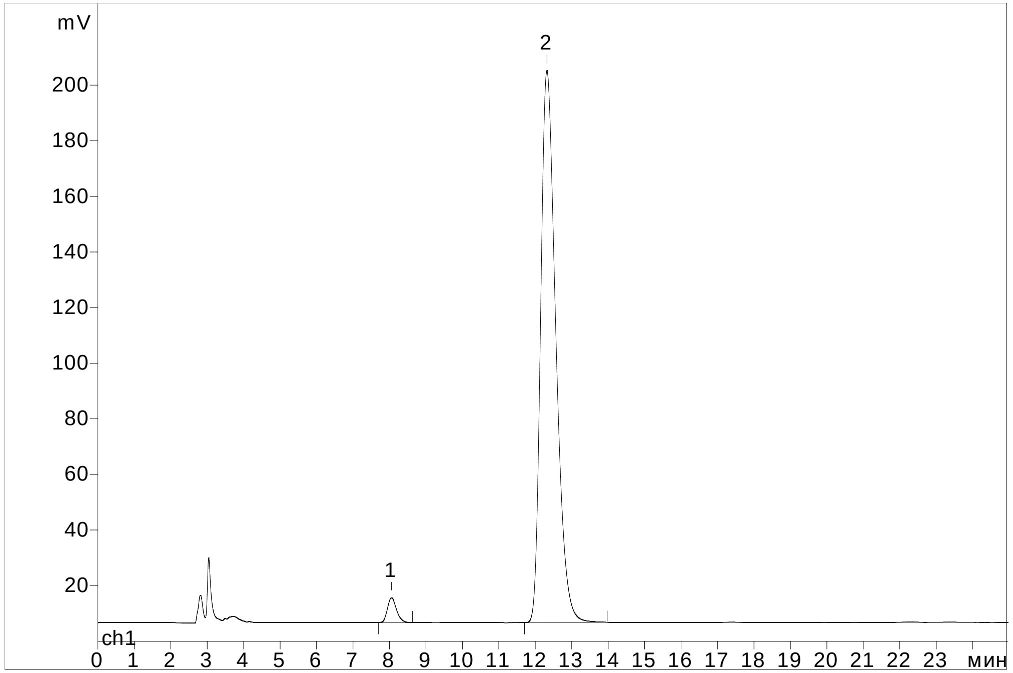

No	Retention<br>мин	Area<br>mV*сек	Area<br>%	Name
1	8.05	154.491	2.63	
2	12.32	5720.528	97.37	
2	25.00	5875.020	100.00	
